# Supplementary material for: Companionship for women/birthing people using antenatal and intrapartum care in England during COVID-19: a mixed-methods analysis of national and organisational responses and perspectives
Source: BMJ Open. 2022 Jan 11;12(1):e051965. doi: 10.1136/bmjopen-2021-051965 (PMC8753093; doi:10.1136/bmjopen-2021-051965)
Supplement: Supplementary data [file bmjopen-2021-051965supp002.pdf]

| Organisation/Code | Name of Document/Link | Date of document |
|---|---|---|
| **AIMS** | | |
| AIMS_1 | Coronavirus and the Maternity Services on AIMS website https://www.aims.org.uk/campaigning/item/coronaviru | 25/03/2020 |
| AIMS _2 | AIMS Response to the Clinical guide for the temporary reorganisation of intrapartum maternity care during the coronavirus pandemic, version 1, published 9/4/20 https://www.aims.org.uk/assets/media/444/aimscomments2020-04-11.pdf | 09/04/2020 |
| AIMS_5 | Submission from AIMS to the Health and Social Care Committee Inquiry: Delivering Core NHS and Care Services during the Pandemic and Beyond https://www.aims.org.uk/assets/media/447/aimssubmissionhealthandsocialcarecommittee2020-05.pdf | 07/05/2020 |
| AIMS_7 | AIMS comments on latest guidance on birth partner restrictions https://www.aims.org.uk/campaigning/item/latest-guidance-birth-partner | 25/09/2020 |
| AIMS_6 | Press Release: Almost Half of NHS Trusts Force Pregnant Women to Attend Vital Maternity Services Alone https://www.aims.org.uk/campaigning/item/trusts-force-pregnant-women-to-attend-alone | 12/10/2020 |
| AIMS_9 | AIMS calls for clearer guidance from NHS England on lifting partner restrictions in maternity https://www.aims.org.uk/campaigning/item/nhse-letter | 21/11/2020 |
| AIMS_8 | AIMS asks Society and College of Radiographers to review guidance on partners and supporters at scans https://www.aims.org.uk/campaigning/item/scor-letter | 12/11/2020 |
| **Birthrights** | | |
| BR_1 | Human rights charity calls for protection of UK women in childbirth during national emergency https://www.birthrights.org.uk/wp-content/uploads/2020/03/Final-Covid-19-Birthrights-31.3.20.pdf | 31/03/2020 |
| BR_8 | Coronavirus and the impact on people with protected characteristics https://www.birthrights.org.uk/wp-content/uploads/2020/05/Birthrights-Covid-19-Maternity-Briefing-women-and-equalities-committee-final.pdf | 01/04/2020 |
| BR_6 | Final letter SOR-BMUS 22nd April Birthrights joint letter with National Maternity Voices, Sands, the Twins Trust and ARC https://www.birthrights.org.uk/wp-content/uploads/2020/04/Final-letter-SoR-BMUS-22-April.pdf | 22/04/2020 |
| BR_12 | Letter to NHSE regarding ongoing visiting restrictions https://www.birthrights.org.uk/wp-content/uploads/2020/08/Letter-to-NHSE-re-ongoing-visiting-restrictions.pdf | 20/08/2020 |

| | | |
|---|---|---|
| BR_14 | Ongoing visitor restrictions in maternity services https://www.birthrights.org.uk/2020/09/14/ongoing-visitor-restrictions-in-maternity-services/ | 14/09/2020 |
| BR_15 | Template letter for ongoing visiting restrictions in maternity services https://www.birthrights.org.uk/template-letters/ | 14/09/2020 |
| BR_16 | Letter on restricting visitors – Sheffield teaching hospitals https://www.birthrights.org.uk/wp-content/uploads/2020/10/Letter-to-Sheffield-15th-October.pdf | 15/10/2020 |
| BR_17 | "Partners are not visitors to maternity services" says Birthrights and partner organisations [joint letter on visiting restrictions to NHSE&I] https://www.birthrights.org.uk/wp-content/uploads/2020/10/Final-letter-to-NHSE-from-coalition-re-visiting-restrictions.pdf | 20/10/2020 |
| BR_19 | Human rights charity calls for easing of visiting restrictions in maternity services press release https://www.birthrights.org.uk/wp-content/uploads/2020/08/Easing-of-visitor-restrictions-press-release-20-August-2020.pdf | 20/08/202 |
| BR_18 | Human rights implications of changes to maternity services during the Covid-19 pandemic Birthrights briefing, July 2020 https://www.birthrights.org.uk/wp-content/uploads/2020/07/Birthrights-Covid-19-Human-Rights-Committee-Briefing-July-2020.pdf | Jul-20 |
| BR_23 | Long term impact of visiting restrictions "could be catastrophic" MPs, academics and campaigners warn NHS England CEO… https://www.birthrights.org.uk/2020/11/15/long-term-impact-of-visiting-restrictions-could-be-catastrophic-mps-academics-and-campaigners-warn-nhs-england-ceo/ | 15/11/2020 |
| **ISUOG** | | |
| ISUOG_6 | ISUOG Safety Committee Position Statement: safe performance of obstetric and gynaecological scans and equipment cleaning in the context of COVID-19 https://www.isuog.org/resource/isuog-safety-committee-position-statement-safe-performance-of-obstetric-and-gynecological-scans-and-equipment-cleaning-in-the-context-of-covid-19.html | 23/03/2020 |
| ISUOG_5 | ISUOG Consensus Statement on organization of routine and specialist obstetric ultrasound services in the context of COVID-19 https://www.isuog.org/resource/wiley-isuog-consensus-statement-on-organization-of-routine-and-specialist-obstetric-ultrasound-services-in-the-context-of-covid-19.html | 31/03/2020 |

| | | |
|---|---|---|
| ISUOG_3 | ISUOG Interim Guidance (COVID-19) during pregnancy & puerperium: Information for healthcare professionals – an update https://obgyn.onlinelibrary.wiley.com/doi/full/10.1002/uog.22061 | 01/05/2020 |
| **NHS England** | | |
| NHSE_1 | Clinical guide for the temporary reorganisation of intrapartum maternity care during the coronavirus pandemic<br>https://madeinheene.hee.nhs.uk/Portals/0/Clinical%20guide%20for%20the%20temporary%20reorganisation%20of%20intrapartum%20maternity%20care.pdf | 09/04/2020 |
| NHSE_2 | Delivering midwifery intrapartum care where local COVID-19 escalation protocols are required to be enacted<br>https://www.nice.org.uk/media/default/about/covid-19/specialty-guides/delivering-midwifery-intrapartum-care-where-local-covid-19-escalation-protocols-are-required-to-be-enact.pdf | 20/06/2020 |
| NHSE_8 | Framework to assist NHS trusts to reintroduce access for partners, visitors and other supporters of pregnant women in English maternity services http://allcatsrgrey.org.uk/wp/download/midwifery/par001599-framework-for-the-reintroduction-of-visitors-throughout-maternity-services-sep-2020.pdf | 08/09/2020 |
| NHSE_9 | Letter to directors of nursing and heads of midwifery<br>(No longer available on website, available on request) | 19/09/2020 |
| NHSE_10 | Lateral flow antigen test for pregnant women and support people: Frequently asked questions<br>https://www.england.nhs.uk/coronavirus/wp-content/uploads/sites/52/2020/12/C0972-lateral-flow-antigen-tests-for-maternity-faqs-december-2020.pdf | 21/12/2020 |
| NHSE_11 | Supporting pregnant women using maternity services during the coronavirus pandemic: Actions for NHS providers Version 1, 14 December 2020<br>https://www.england.nhs.uk/coronavirus/wp-content/uploads/sites/52/2020/12/C0961-Supporting-pregnant-women-using-maternity-services-during-the-coronavirus-pandemic-actions-for-NHS-provi.pdf?fbclid=IwAR0w9hbJSbH1gYYPVhGtE8zRAvwd8PidPkPsEaDnhxzlSumFl7t6byshst8 | 14/12/2020 |
| **RCOG** | | |
| RCOG/RCM_1c | Coronavirus (COVID-19) Infection in Pregnancy Information for healthcare professionals Version 3<br>https://www.rcm.org.uk/media/3799/coronavirus-covid-19-infection-in-pregnancy-v3-20-03-18.pdf | 18/03/2020 |
| RCOG/RCM_1d | Coronavirus (COVID-19) Infection in Pregnancy Information for healthcare professionals Version 4<br>https://www.rcm.org.uk/media/3800/2020-03-21-covid19-pregnancy-guidance.pdf | 21/03/2020 |

| | | |
|---|---|---|
| RCOG/RCM_1e | Coronavirus (COVID-19) Infection in Pregnancy Information for healthcare professionals Version 5<br>https://www.rcm.org.uk/media/3824/2020-03-28-covid19-pregnancy-guidance.pdf | 28-03-202 |
| RCOG/RCM_1f | Coronavirus (COVID-19) Infection in Pregnancy Information for healthcare professionals Version 6<br>http://www.e-lactancia.org/media/papers/2020-04-03-coronavirus-covid-19-infection-in-pregnancy.pdf | 03/04/2020 |
| RCOG/RCM_1g | Coronavirus (COVID-19) Infection in Pregnancy Information for healthcare professionals Version 7<br>https://www.sbmfc.org.br/wp-content/uploads/2020/04/2020-04-09-coronavirus-covid-19-infection-in-pregnancy.pdf | 09/04/2020 |
| RCOG_9 | Guidance for rationalising early pregnancy services in the evolving coronavirus (COVID-19) pandemic<br>https://www.rcog.org.uk/globalassets/documents/guidelines/2020-05-15-guidance-for-rationalising-early-pregnancy-services-in-the-evolving-coronavirus-covid-19-pandemic.pdf | 15/05/2020 |
| RCOG_11 | Framework for staffing of obstetrics and gynaecology units during the COVID-19 pandemic<br>https://www.rcog.org.uk/globalassets/documents/guidelines/2020-05-22-framework-for-staffing-of-obstetrics-and-gynaecology-units-during-the-covid-19-pandemic.pdf | 22/05/2020 |
| RCOG_6 | Principles for the testing and triage of women seeking maternity care in hospital settings, during the COVID-19 pandemic<br>https://www.rcog.org.uk/globalassets/documents/guidelines/2020-05-29-principles-for-the-testing-and-triage-of-women-seeking-maternity-care-in-hospital-settings-during-the-covid-19-pandemic.pdf | 29/05/2020 |
| RCOG/RCM_1j | Coronavirus (COVID-19) Infection in Pregnancy Information for healthcare professionals Version 10 https://www.rcm.org.uk/media/4113/2020-06-04-coronavirus-covid-19-infection-in-pregnancy.pdf | 04/06/2020 |
| RCOG_7b | Guidance for antenatal and postnatal services in the evolving coronavirus (COVID-19) pandemic<br>https://www.rcog.org.uk/globalassets/documents/guidelines/2020-07-10-guidance-for-antenatal-and-postnatal.pdf | 10/07/2020 |
| RCOG_14 | Restoration and Recovery: Priorities for Obstetrics and Gynaecology Version 2.1<br>https://www.rcog.org.uk/globalassets/documents/guidelines/2020-06-26-restoration-and-recovery---priorities-for-obstetrics-and-gynaecology.pdf | 26/06/2020 |
| RCOG/RCM_1k | Coronavirus (COVID-19) infection in pregnancy Information for healthcare professionals Version 11 https://www.rcog.org.uk/globalassets/documents/guidelines/2020-07-24-coronavirus-covid-19-infection-in-pregnancy.pdf | 24/07/2020 |

| | | |
|---|---|---|
| RCOG_2 | Guidance for provision of midwife-led settings and home birth in the evolving coronavirus (COVID-19) pandemic Version 1.3<br>https://www.rcog.org.uk/globalassets/documents/guidelines/2020-07-10-guidance-for-provision-of-midwife-led.pdf | 10/07/2020 |
| RCOG_4 | Guidance for fetal medicine units (FMUs) in the evolving coronavirus (COVID-19) pandemic<br>https://www.rcog.org.uk/globalassets/documents/guidelines/2020-11-23-guidance-for-fetal-medicine-units.pdf | 10/07/2020 |
| RCOG_5 | Guidance for antenatal screening and ultrasound in pregnancy in the evolving coronavirus (COVID-19) pandemic<br>https://www.rcog.org.uk/globalassets/documents/guidelines/2020-07-10-guidance-for-antenatal-screening.pdf | 10/07/2020 |
| RCOG_15 | Joint RCOG & RCM Statement - Planning for Winter 2020/21: reducing the impact of COVID-19 on maternity services in the UK<br>https://www.rcog.org.uk/globalassets/documents/guidelines/2020-10-08-rcog_rcm_winter_secondwave_statement.pdf | 08/10/2020 |
| RCOG_7 | Guidance for antenatal and postnatal services in the evolving coronavirus (COVID-19) pandemic<br>Version 3<br>https://www.rcog.org.uk/globalassets/documents/guidelines/2020-10-21-guidance-for-antenatal-and-postnatal-services-in-the-evolving-coronavirus-covid-19-pandemic-v3.pdf | 21/10/2020 |
| RCOG/RCM_1 | Coronavirus (COVID-19) infection in pregnancy Information for healthcare professionals Version 12 https://www.rcm.org.uk/media/4383/2020-10-14-coronavirus-covid-19-infection-in-pregnancy-v12.pdf | 14/10/2020 |
| RCOG_17 | RCOG responds to revised NHS coronavirus guidelines https://www.rcog.org.uk/en/news/pregnant-women-allowed-partner-support-at-all-times-in-updated-nhs-guidelines/ | 16/12/2020 |
| **RCM** | | |
| RCM_8 | Induction of labour in a pandemic: A rapid analytic scoping review https://www.rcm.org.uk/media/3924/professional-clinical-briefing-no-7-intrapartum-care-with-symptomsmrd010520.pdf | 04/04/2020 |
| RCM_2 | Optimising maternity services and maternal and newborn outcomes in a pandemic. A rapid analytic scoping review https://www.rcm.org.uk/media/3869/rapid-review-optimising-maternity-services-for-rcm-v4-8-april.pdf | 08/04/2020 |

| | | |
|---|---|---|
| RCM_15 | Guidance for midwives, student midwives and maternity support workers providing community-based care during the Covid-19 pandemic https://www.rcm.org.uk/media/3900/home-visit-guidance-for-midwives.pdf | 09/04/2020 |
| RCM_11 | Rapid Analytic Review: Labour and Birth Companionship in a pandemic https://www.rcm.org.uk/media/3951/birth-companionship-in-a-pandemic-master-27-04-2020-002.pdf | 27/04/2020 |
| RCM_12 | RCM Clinical Briefing Sheet: 'freebirth' or 'unassisted childbirth' during the COVID-19 pandemic https://www.rcm.org.uk/media/3923/freebirth_draft_30-april-v2.pdf | 30/04/2020 |
| RCM_16 | Optimising mother-baby contact and infant feeding in a pandemic. Rapid analytic review https://www.rcm.org.uk/media/4096/optimising-infant-feeding-and-contact-rapid-review-19th-may-2020-submitted.pdf | 19/05/2020 |
| RCM_9 | Face-coverings and care in labour for all women https://www.rcm.org.uk/media/4151/clinical-briefing-face-mask.pdf | 01/07/2020 |
| RCM_5 | Antenatal Care for women without suspected or confirmed COVID-19 and living in a symptom free household https://www.rcm.org.uk/media/4180/clinical-guidance-briefing-one-antenatal-care-for-women-without-symptomsfinalv4-1.pdf | 17/07/2020 |
| RCM_4 | RCM Briefing on Re-introduction of visitors to Maternity Units across the UK during the COVID-19 pandemic https://www.rcm.org.uk/media/4161/rcm-briefing-on-reintroduction-of-visitors-to-maternity-units-in-the-covid-pandemic-003.pdf | 15/07/2020 |
| RCM_3 | Virtual consultations https://www.rcm.org.uk/media/4192/virtual-consultations-v20-24-july-2020-review-24-august-2020-1.pdf | 24/07/2020 |
| RCM_7 | Guidance for intrapartum care for women with COVID-19 https://www.rcm.org.uk/media/4193/intrapartum-care_with-covid-19_20200724-v9-1.pdf | 24/07/2020 |
| RCM_10 | RCM Clinical Briefing Sheet - Waterbirth during the COVID-19 Pandemic https://www.rcm.org.uk/media/4188/waterbirth-during-covid-19-july-20.pdf | 19/07/2020 |
| RCM_31 | RCM & RCOG urge NHS England to publish guidance on partners during pregnancy https://www.rcm.org.uk/media-releases/2020/september/rcm-and-rcog-urge-nhs-england-to-publish-guidance-on-partners-during-pregnancy | 08/09/2020 |

| | | |
|---|---|---|
| RCM_27 | Birth partners https://www.rcm.org.uk/media/4339/birth-partners-v2-220920.pdf | 20/09/2020 |
| RCM_30 | Leading Royal Colleges urge the NHS to learn lessons and avoid redeploying maternity staff ahead of second wave https://www.rcog.org.uk/en/news/leading-royal-colleges-urge-the-nhs-to-learn-lessons-and-avoid-redeploying-maternity-staff-ahead-of-second-wave/ | 30/09/2020 |
| RCM_28 | RCM letter to The Times responding to Times column on COVID rules and pregnancy https://www.rcm.org.uk/media-releases/2020/september/rcm-letter-to-the-times-responding-to-times-column-on-covid-rules-and-pregnancy/ | 02/10/2020 |
| RCM_38 | Partners and visitors<br>https://www.rcm.org.uk/partners-and-visitors/ | Oct-20 |
| RCM_39 | Reduce infection risks to keep pregnant women and their babies safe, says RCM https://www.rcm.org.uk/media-releases/2020/october/reduce-infection-risks-to-keep- | 02/11/2020 |
| RCM_41 | Midwives call for common sense on maternity visiting guidance<br>https://www.rcm.org.uk/media-releases/2020/december/midwives-call-for-common-sense-on-maternity-visiting-guidance/ | 15/12/2020 |
| **Sands** | | |
| Sands_1 | Sands feedback: experiences of pregnant women, bereaved parents and families during the COVID-19 pandemic. No longer on website, available on request | 19-05-2020 to<br>27/05/2020 |
| Sands_1 | Written evidence from SANDS to parliamentary committee Written evidence submitted by SANDS (DEL0092) https://committees.parliament.uk/writtenevidence/4073/html/ | May-20 |
| Sands_4 | New NHS guidance on partners of pregnant women in England attending scans https://www.sands.org.uk/about-sands/media-centre/news/2020/09/new-nhs-guidance-partners-pregnant-women-england-attending | 09/09/2020 |
| **SoR** | | |
| SoR_11 | Obstetric ultrasound examinations during the COVID-19 pandemic.<br>Recording of NHS examinations and the use of private ultrasound clinics: A joint statement from the Society and College of Radiographers (SCoR), the Royal College of Midwives (RCM), the Royal College of Obstetricians and Gynaecologists (RCOG) and the British Medical Ultrasound Society (BMUS). https://archive.sor.org/sites/default/files/document-versions/obstetric_ultrasound_examinations_during_the_covid-19_pandemic_v2_0.pdf | 24/04/2020 |

| | | |
|---|---|---|
| SoR_9 | Ultrasound: Frequently asked questions (FAQs) version 1.7 previously on website – superseded by later versions (SoR & BMUS) | 27/04/2020 |
| SoR_10 | Ultrasound: Frequently asked questions (FAQs) version 1.8 previously on website – superseded by later versions (SoR & BMUS) | 21/05/2020 |
| SoR_12 | Obstetric ultrasound examinations during the COVID-19 pandemic.<br>Recording of NHS examinations and the use of private ultrasound clinics: A joint statement from the Society and College of Radiographers (SCoR), the Royal College of Midwives (RCM), the Royal College of Obstetricians and Gynaecologists (RCOG) and the British Medical Ultrasound Society (BMUS). https://archive.sor.org/sites/default/files/document-versions/obstetric_ultrasound_examinations_during_the_covid-19_pandemic_v2_0.pdf | 27/05/2020 |
| SoR_6 | SoR demands NHS Trusts defend sonographers from populist attacks<br>https://www.sor.org/news/sor-demands-nhs-trusts-defend-sonographers-populist-attacks | 04/11/2020 |
| SoR_4 | Ultrasound: Frequently asked questions (FAQs) version 1.10 previously on website – superseded by later versions (SoR & BMUS) | 04/11/2020 |
| SoR_7 | Letter from SoR Resisting populist pressure to minimise the risks to ultrasound staff & patients.<br>Document supplied by SoR | 06/11/2020 |
| SoR_8 | Webpage: New national guidance on partners attending antenatal scans<br>https://www.sor.org/news/obstetrics-and-gynaecology/new-national-guidance-on-partners-attending-antena | 16/12/2020 |

**t**
