# Supplementary material for: Companionship for women/birthing people using antenatal and intrapartum care in England during COVID-19: a mixed-methods analysis of national and organisational responses and perspectives
Source: BMJ Open. 2022 Jan 11;12(1):e051965. doi: 10.1136/bmjopen-2021-051965 (PMC8753093; doi:10.1136/bmjopen-2021-051965)
Supplement: Supplementary data [file bmjopen-2021-051965supp003.pdf]

Supplementary File 3: Documentary and interview data sources for the six key themes

Themes	*Documents*	*Stakeholders*
Postcode lottery of care	BR_8, BR_12, BR_17, BR_19, BR_18; RCOG/RCM_1e, RCOG_9, RCOG_15, RCM/RCOG_1l, RCOG_15, RCOG_17; Sands_1; RCM_5, RCM_7, RCM_31, RCM_38, RCM_39, RCM_41; NHSE_8, NHSE_9, NHSE_11; AIMS_2,AIMS_7, AIMS_8; SoR_2, SoR_4, SoR_5, SoR_6, SoR_7, SoR_8, SoR_9, SoR_10, SoR_11, SoR_12	7, 12, 13, 15, 17, 18, 20
Confusion and stress around 'rules'	BR_8, BR_12, BR_14, BR_16, BR_19; RCM_31; AIMS_7	12, 15, 19 20
Unintended Consequences	BR_1, BR_8, BR_12, BR_16, BR_17, BR_23; RCOG/RCM_1e, RCOG/RCM_1j , RCOG/RCM_1k, RCOG/RCM_1l; Sands_1; RCM_2, RCM_7, RCM_8, RCM_11, RCM_16 ; NHSE_1, NHSE_2, , NHSE_11; AIMS_8;	2, 4, 5, 8, 12, 13, 15, 17, 18, 19, 20
Need for flexibility	BR_8, BR_6, BR_15, BR_18; Sands_1; RCM_4, RCM_41; NHSE_8; AIMS_2, AIMS_8; SoR_12	3, 7, 10, 13, 17, 21
Acceptable time for support	BR_1, BR_6, BR_8, BR_16, BR_17, BR_18, BR_23; RCOG_7; Sands_1; RCM_3, RCM_8, RCM_11, RCM_27, RM_28; AIMS_2, AIMS _5, AIMS_8; SoR_2, SoR_4, SoR_5, SoR_9, SoR_10, SoR_11, SoR_12	5, 7, 13, 15, 16, 17, 18, 19, 20, 21, 26
Loss of human rights for gain in infection control	BR_16, BR_17, BR_19, BR_23, BR_8, BR_18; RCOG/RCM_1g , RCOG/RCM_1c, RCOG/RCM_1e, RCOG/RCM_1f , RCOG_9, RCOG_6; RCM_11, RCM_30, RCM_28,RCM_41, RCM_39, RCM-38, RCM_11; NHSE_9, NHSE_10; SoR_2, SoR_6, SoR_5, SoR_8, SoR_7, SoR_4, SoR_9, SoR_10, SoR_11, SoR_12; ISUOG 5, ISUOG_6	3, 5, 11, 14, 17
