# Supplementary material for: Companionship for women/birthing people using antenatal and intrapartum care in England during COVID-19: a mixed-methods analysis of national and organisational responses and perspectives
Source: BMJ Open. 2022 Jan 11;12(1):e051965. doi: 10.1136/bmjopen-2021-051965 (PMC8753093; doi:10.1136/bmjopen-2021-051965)
Supplement: Author's manuscript [file bmjopen-2021-051965.draft_revisions.pdf]

BMJ Open is committed to open peer review. As part of this commitment we make the peer review history of every article we publish publicly available.

When an article is published we post the peer reviewers' comments and the authors' responses online. We also post the versions of the paper that were used during peer review. These are the versions that the peer review comments apply to.

The versions of the paper that follow are the versions that were submitted during the peer review process. They are not the versions of record or the final published versions. They should not be cited or distributed as the published version of this manuscript.

BMJ Open is an open access journal and the full, final, typeset and author-corrected version of record of the manuscript is available on our site with no access controls, subscription charges or pay-per-view fees (http://bmjopen.bmj.com).

If you have any questions on BMJ Open's open peer review process please email info.bmjopen@bmj.com

BMJ Open

Companionship for women using English maternity services during COVID-19: National and organisational perspectives

Journal:	*BMJ Open*
Manuscript ID	bmjopen-2021-051965
Article Type:	Original research
Date Submitted by the Author:	05-Apr-2021
Complete List of Authors:	Thomson, Gillian; University of Central Lancashire, School of Health<br>Balaam, Marie-Claire; University of Central Lancashire,<br>Nowland (Harris), Rebecca; University of Central Lancashire, School of Nursing<br>Crossland, Nicola; University of Central Lancashire, School of Health<br>Moncrieff, Gill; University of Central Lancashire, School of Community Health & Midwifery<br>Heys, Stephanie; North West Ambulance Service NHS Trust<br>Sarian, Arni; University of Central Lancashire, School of Medicine<br>Cull , Joanne; University of Central Lancashire, School of Community Health & Midwifery<br>Downe, Soo; University of Central Lancashire, research in childbirth and health
Keywords:	GYNAECOLOGY, Organisational development < HEALTH SERVICES ADMINISTRATION & MANAGEMENT, Risk management < HEALTH SERVICES ADMINISTRATION & MANAGEMENT, Public health < INFECTIOUS DISEASES, QUALITATIVE RESEARCH

SCHOLARONE™
Manuscripts

I, the Submitting Author has the right to grant and does grant on behalf of all authors of the Work (as defined in the below author licence), an exclusive licence and/or a non-exclusive licence for contributions from authors who are: i) UK Crown employees; ii) where BMJ has agreed a CC-BY licence shall apply, and/or iii) in accordance with the terms applicable for US Federal Government officers or employees acting as part of their official duties; on a worldwide, perpetual, irrevocable, royalty-free basis to BMJ Publishing Group Ltd ("BMJ") its licensees and where the relevant Journal is co-owned by BMJ to the co-owners of the Journal, to publish the Work in this journal and any other BMJ products and to exploit all rights, as set out in our licence.

The Submitting Author accepts and understands that any supply made under these terms is made by BMJ to the Submitting Author unless you are acting as an employee on behalf of your employer or a postgraduate student of an affiliated institution which is paying any applicable article publishing charge ("APC") for Open Access articles. Where the Submitting Author wishes to make the Work available on an Open Access basis (and intends to pay the relevant APC), the terms of reuse of such Open Access shall be governed by a Creative Commons licence – details of these licences and which Creative Commons licence will apply to this Work are set out in our licence referred to above.

Other than as permitted in any relevant BMJ Author's Self Archiving Policies, I confirm this Work has not been accepted for publication elsewhere, is not being considered for publication elsewhere and does not duplicate material already published. I confirm all authors consent to publication of this Work and authorise the granting of this licence.

For peer review only

Title: Companionship for women using English maternity services during COVID-19: National and organisational perspectives

Authorship

*Gill Thomson, Professor in Perinatal Health, School of Community Health & Midwifery, University of Central Lancashire, Preston, UK. PR1 2HE. GThomson@uclan.ac.uk

Marie-Clare Balaam, School of Community Health & Midwifery, University of Central Lancashire, Preston, UK.

Rebecca Nowland, School of Nursing, University of Central Lancashire, Preston, UK.

Nicola Crossland, School of Community Health and Midwifery, University of Central Lancashire, Preston, UK. refle

Gill Moncrieff, School of Community Health & Midwifery, University of Central Lancashire, Preston, UK.

Stephanie Heys, Consultant Midwife, North West Ambulance Service, Bolton, UK.

Arni Sarian, School of Medicine, University of Central Lancashire, Preston, UK.

Joanne Cull, School of Community Health & Midwifery, University of Central Lancashire, Preston, UK.

Soo Downe, School of Community Health & Midwifery, University of Central Lancashire, Preston, UK.

And the ASPIRE-COVID19 Collaborative Group

* Corresponding author

Word count: 5,769

ASPIRE-COVID19 Collaborative Group

Co-investigators
Soo Downe; University of Central Lancashire, George Ellison; University of Central Lancashire, Alan Fenton; Newcastle upon Tyne Hospitals NHS Foundation Trust, Alexander Heazell; University of Manchester, Ank de Jonge; Amsterdam University Medical Center, Carol Kingdon; University of Central Lancashire, Zoe Matthews; University of Southampton, Alexandra Severns; NHS England and NHS Improvement North West, Gill Thomson; University of Central Lancashire, Anastasia Topalidou; University of Central Lancashire, Alison Wright; Royal Free Teaching Hospital in London

Research Staff
Naseerah Akooji; University of Central Lancashire, Marie-Clare Balaam; University of Central Lancashire, Jo Cull; University of Central Lancashire, Lauri van den Berg; Amsterdam University Medical Center, Nicola Crossland; University of Central Lancashire, Claire Feeley;

For peer review only - http://bmjopen.bmj.com/site/about/guidelines.xhtml

University of Central Lancashire, Beata Franso; Amsterdam University Medical Center, Steph Heys; University of Central Lancashire, Gill Moncrieff; University of Central Lancashire, Rebecca Nowland; University of Central Lancashire, Arni Sarian; University of Central Lancashire

Steering committee

Maria Booker; Birthrights, Jane Sandall; Kings College London, Jim Thornton (chair); The University of Nottingham, Tisian Lynskey-Wilkie; University of Central Lancashire, Vanessa Wilson; Lancashire and South Cumbria Local Maternity System

Stakeholder Group

Rebecca Abe and Tinuke Awe; FivexMore, Toyin Adeyinka; MVP BAME group, Ruth Bender-Atik; The Miscarriage Association, Lia Brigante; RCM, Rebecca Brione; Birthrights, Franka Cadée; International Confederation of Midwives, Elizabeth Duff; Expert, postnatal care, Tim Draycott; Royal College of Obstetricians and Gynaecologists, Duncan Fisher; Fathers included/Family included/ the Family Initiative, Annie Francis; Independent Midwifery Advisor, Arie Franx; Erasmus MC, Lucy Frith; University of Liverpool, Louise Griew; National Maternity Voices, Clea Harmer; Sands, Caroline Homer; Burnet Institute, Australia, Marian Knight; National Perinatal Epidemiology Unit, Amali Lokugamage; Whittington Health NHS Trust/ University College London, Amanda Mansfield; London Ambulance Service Trust, Neil Marlow; University College London, Trixie Mcaree; NHS England, David Monteith; Grace in Action, Keith Reed; Twins Trust, Yana Richens; UCL & City University, Lucia Rocca-Ihenacho; Midwifery Unit Network, Mary Ross-Davie; Royal College of Midwives Scotland, Seana Talbot; BirthWise NI, Myles Taylor; British Maternal and Fetal Medicine Society, Maureen Treadwell; Birth Trauma Association

For peer review only

ABSTRACT

Objectives: To explore the impact of COVID-19 on companionship for women using maternity services in England, as part of the Achieving Safe and Personalised maternity care In Response to Epidemics (ASPIRE COVID-19 UK) study.

Setting: Maternity care provision in England.

Participants: Interviews were held with 26 national governmental, professional, and service-user organisation leads including representatives from the Royal College of Midwives, NHS England, Birthrights and AIMS (July-Dec). Other data included public-facing outputs logged from 25 maternity Trusts (Sept/Oct) and data extracted from 78 documents from 8 key governmental, professional and service-user organisations that informed national maternity care guidance and policy (Feb-Dec).

Results: Six themes emerged: '*Postcode lottery of care*' highlights variations in companionship practices, '*Confusion and stress around 'rules''* relates to a lack of and variable information concerning companionship, '*Unintended consequences'* concerns the negative impacts of restricted companionship on service-users and staff, '*Need for flexibility'* highlights concerns about applying companionship policies irrespective of need, ''*Acceptable' time for support'* highlights variations in when and if companionship was 'allowed' antenatally and intrapartum; and '*Loss of human rights for gain in infection control'* emphasizes how a predominant focus on infection control was at a cost to psychological safety and women's human rights.

Conclusions: Policies concerning companionship have been inconsistently applied within English maternity services during the COVID-19 pandemic. In some cases, policies were not justified by the level of risk, and were applied indiscriminately regardless of need. This was associated with psychological harms for some women and staff. There is an urgent need to determine how to balance risks and benefits sensitively and flexibly and to optimise outcomes during the current and future crisis situations.

Strengths and limitations of this study

- This is the first paper to consider links between policy and practice in companionship in maternity care during the COVID-19 pandemic;
- Data triangulation across stakeholders, policy and practice provides nuanced and context related perspectives on why and how companionship was impacted;

For peer review only - http://bmjopen.bmj.com/site/about/guidelines.xhtml

- Stakeholders included representatives from all key agencies involved in maternity care;
- Practice related issues were collected from the maternity Trust website and social media-based public facing information, which may or may not reflect actual care practices;
- The study does not include information directly reported by parents and healthcare professionals.

INTRODUCTION

In many cultures around the world, pregnancy is framed as a social event rather than a clinical condition (1-3). Even where pregnancy and birth are framed as medically risky, social support is expected during pregnancy, birth, and the postnatal period. Such support is usually provided through ongoing family and community relationships, often by female relatives and friends, or community members (4). In the postpartum period most cultures recognise a period of between about 4 and 6 weeks during which the mother is supported to recover from the birth, to establish a relationship with the baby, and to adjust to motherhood (4).

As maternity care has become more hospital based, the power to determine who should accompany women in clinics and facility settings has shifted to the organisation, and its employees(5). In the early decades of mass hospitalisation for antenatal care and birth in the UK, companionship for women was banned or restricted, on the grounds of infection control, overcrowding, privacy for others, and defence from potential litigation if the accompanying partner or partners witness activities they perceive to be negligent or dangerous (6-8). More recently the evidence for the value of companionship in the intrapartum period has increased (1) and it is strongly recommended within global guidelines (9). Though restrictions still persist in some health economies around the world, antenatal, intrapartum and postnatal companionship policies in clinics and hospitals in the UK have become increasingly liberal over the last 40 years(10).

The COVID-19 pandemic brought the issue of companions in health facilities worldwide into sharp focus(11). In terms of maternity care, there have been anecdotal accounts of wide variations concerning if and whether women have been permitted companionship at various points throughout the maternity episode, both between countries, and across different care providers within countries. Concerns about women being alone for antenatal contacts, for

For peer review only - http://bmjopen.bmj.com/site/about/guidelines.xhtml

ultrasound scans (especially when there is bad news), during labour and birth, and in the early postnatal period, have been widespread and global in media reports (for example: (11-13)).

To understand how companionship in maternity care during COVID-19 was operationalised organisationally in England, this paper presents an analysis of relevant national level policy documents; interviews with key stakeholders; and a review of public facing information produced by 25 purposively selected maternity providers in England.

METHODS

The current study is part of a larger mixed-methods, observational, multi-site comparative study - Achieving Safe and Personalised maternity care In Response to Epidemics (ASPIRE COVID-19 UK).

Data relating to companionship in maternity care were extracted from national policy level documents and interviews with national stakeholders and mapped to analysis of public-facing communication channels from 25 Trusts (maternity care organisations). The Trusts were selected using maximum variation sampling, based on macro-level factors impacting upon health inequalities (area level deprivation): meso-level considerations relating to the organisation (Care Quality Commission (CQC) rating and maternal and neonatal mortality figures): and micro-level factors relating to the local population type and local organisational responses to the pandemic.

Data collection

Documentary review

Guidelines, position papers, and reports relating to maternity care were collected prospectively between February and December 2020 from key governmental, professional, and service-user sources that inform national guidance and policy in relation to maternity care.

Trust-level public facing communication about maternity service provision

Data related to companionship in pregnancy, labour and birth, and the postpartum period were extracted from Trust websites and official Twitter, Facebook, and Instagram feeds, between September and October 2020.

For peer review only - http://bmjopen.bmj.com/site/about/guidelines.xhtml

Interviews

Individuals from relevant national governmental, professional, and service-user organisation leads involved in maternity care were approached by email and provided with an information sheet about the study. Semi-structured interviews were held July-December 2020 (using Microsoft Teams). Interviews lasted between 45-60 minutes, were audio recorded and transcribed in full. The interview schedule (see Supplementary File 1) explored stakeholders' perceptions and experiences of what, why and how changes in maternity care delivery had been made during the pandemic, how changes had been monitored and assessed, and their views on facilitators and barriers to those changes. Interviews ceased when representation from all key organisations had been captured.

Ethics approval

Ethics approval to undertake the interviews was obtained from the Health ethics sub-committee from the lead author's institution (project no: 0079). Participants were asked if they would like to check any public facing quotes prior to publications as part of the consent process; quotes were returned to seven participants for feedback, and four required slight changes to be made.

Data analysis

Data analysis was undertaken using a modified framework approach, using inductive and deductive methods (14). All the documents were searched for references to companionship. Relevant data segments were extracted and recorded in excel files. An initial data analysis framework was created by mapping the information from the national level documents into codes, and then collapsing them into key organising themes. This framework was then used to map and organise the interview data, with ongoing refinements to ensure that all the data were represented. The Trust-level data were subjected to descriptive content analysis (15). This involved the data being mapped to different aspects of care (e.g. appointments, ultrasound, induction, labour/birth). The Trust level data were then integrated into the thematic framework for reporting purposes. Four members of the team were involved in this process, and the final framework was agreed by all named authors.

For peer review only - http://bmjopen.bmj.com/site/about/guidelines.xhtml

Public and Patient Involvement

This study was funded under a rapid response call and while no formal PPI involvement in the original design of the study, UK service-user leads (Maternity Voices Partnership) and members of third-sector organisations are involved as co-investigators, steering group and advisory members for the project, to ensure that service-user inputs has been considered at every stage of the study.

Reflexivity

The authors and members of the collaborating group associated with this study are from a range of academic and clinical backgrounds including midwifery, psychology, obstetrics, neonatology, sociology, and social statistics. All the authors are female, and the four interviewers are experienced in undertaking qualitative interviews. One had previously collaborated with some of the interviewees. All authors believe that women and birthing people highly value companionship during key moments in their maternity care, and that, for many fathers/co-parents, being present is more than just being a visitor or a supporter. From her psychological background, RN also believes that birth supporters other than fathers/coparents play a significant role during childbirth in promoting psychological well-being. As midwives, SD, GM, JC, and SH view companionship for women throughout labour as a normative practice.

RESULTS

A total of 50 individuals were approached to participate and interviews were held with twenty-six stakeholders (see Table 1). While some stakeholders did not respond to the requests, others provided names of individuals who they considered would be more suitable.

Table 1. List of stakeholders interviewed

Stakeholder	Organisation/Role
1	Obstetrician with a national role
2	Birth Trauma Association
3	Maternity Voices Partnership (BAME)
4	Royal College of Midwifery Scotland

For peer review only - http://bmjopen.bmj.com/site/about/guidelines.xhtml

5	National Maternity Voices Partnerships
6	BLISS
7	Birthrights
8	Midwife with a national role
9	Consultant midwife with a national role
10	Sands
11	Neonatologist with a national role
12	Maternity Voices Partnership
13	AIMS
14	Senior Midwife Leader
15	Professor of Midwifery/ previous Head of Midwifery
16	Twins Trust
17	NHS England and NHS improvement
18	Independent Midwifery Advisor
19	Maternity and Neonatal Services Manager (N. Ireland)
20	Miscarriage Association
21	Midwife with a national role
22	Owner of a doula training company
23	Consultant anaesthetist
24	Midwife Sonographer
25	Clinical Network Lead (Wales)
26	The Society and College of Radiographers

Documents were collected from nine governmental, professional, and service-user sources (Box 1), with a total of 78 documents providing evidence for the paper (see Supplementary File 2 for full details/references).

For peer review only - http://bmjopen.bmj.com/site/about/guidelines.xhtml

Box 1: Organisations included for documentary analysis

Sands
AIMS
Royal College of Midwives (RCM)
Royal College of Obstetricians and Gynaecologists (RCOG)
Society of Radiographers (SoR)
International Society of Ultrasound in Obstetrics and Gynaecology (ISUOG)
NHS England (NHSE)
Birthrights (BR)

For peer review only

The public facing data logged between September and October 2020 demonstrated a very wide range of policies and practices between the 25 included Trusts for companionship during four specific maternity care episodes (antenatal scanning; antenatal appointments; antenatal ward stays; and intrapartum) (see table two). While this could be explained by different COVID-19 infection exposure rates, this may not explain variation between Trusts in the same region.

Table 2: Public facing information on maternity service companionship policies in 25 English maternity trusts, September to October 2020

	**Antenatal**			**Intrapartum**		
	**Ultrasound**	**Antenatal appointments**	**Antenatal ward**	**Number of birth partners**	**Timing**	**Induction of labour**
Greater London 1	Unaccompanied	Unaccompanied	Booked time slot	One	Throughout labour	Partner allowed if assessed as needing support
Greater London 2	Partner allowed (20 weeks) *	Unaccompanied	2-6pm	Two	Not specified	No information
Greater London 3	Partner allowed (20 weeks)	No details	No visitors	One (two if additional need identified)	Established labour	No information
Greater London 4	Unaccompanied	Unaccompanied	No visitors	One	Established labour	No information
Greater London 5	Partner allowed	Unaccompanied	Daytime	One (two if need identified)	Not specified	No information
Greater London 6	Partner allowed	No details	Daytime	One	Throughout labour	Partner allowed daytime only
South East 1	Unaccompanied	One companion	Booked time slot	One	Not specified	No information

For peer review only - http://bmjopen.bmj.com/site/about/guidelines.xhtml

South East 2	Partner allowed (12 and 20 weeks)	No details	Daytime	Two	Not specified	Partner allowed
South East 3	Unaccompanied	Unaccompanied	Daytime	One	Throughout	No information
South West 1	Partner allowed (12 and 20 weeks)	Unaccompanied	Booked time slot	One	Not specified	Daytime only
South West 2	Partner allowed (20 weeks)	Unaccompanied	No visitors	One	Throughout	Partner not allowed until labour has started
South West 3	Partner allowed (20 weeks)	No details	No information	One	Throughout	Partner allowed daytime or if additional support needed
West Midlands 1	Unaccompanied	Unaccompanied	No visitors	One	Not specified	No information
West Midlands 2	Unaccompanied	Unaccompanied	No information	One	Not specified	Partner allowed if language/communicatio n needs
East Midlands 1	Partner allowed (12 and 20 weeks)	Unaccompanied	No information	One	Throughout	No information
East of England 1	Partners allowed	No information	Booked time slot	One	Throughout	No information
East of England 2	Partner allowed (20 weeks)	Unaccompanied	No information	One	Established labour	Partner not allowed until labour has started
East of England 3	Partner allowed (12 and 20 weeks)¶	Unaccompanied	2-6 pm	One	Not specified	No information
Yorkshire and Humber 1	Partners allowed	No details	No visitors	One	Not specified	Partner not allowed until labour has started
Yorkshire and Humber 2	Partner allowed (12 weeks)	No details	Booked time slot between 1-5pm	One	Throughout	No information
North West 1	Partner allowed (12 and 20 weeks)	Unaccompanied	Booked time slot	One	Throughout	One partner allowed
North West 2	Partners allowed	Unaccompanied	No information	Two	Not specified	No information
North West 3	Partner allowed (20 weeks)	Unaccompanied	No information	Two	Not specified	No information
North East 1	Partner allowed (12 and 20 weeks)†	Unaccompanied	No information	One	Not specified	No information
North East 2	Partners allowed	No details	No information	One	Not specified	No information

For peer review only - http://bmjopen.bmj.com/site/about/guidelines.xhtml

* Phone call offered if clinical concerns

¶ Video offered of a small section at the end of scan

† Phone call offered (end of scan)

Details of themes

Overall, six themes emerged from the final synthesis of the data sets. An overview of the documents and interviews that generated data for each theme is presented in Table 3.

Table 3: Documentary and interview data sources for the six key themes

Themes	*Documents*	*Stakeholders*
Postcode lottery of care	BR_8, BR_12, BR_17, BR_19, BR_18; RCOG/RCM_1e, RCOG_9, RCOG_15, RCM/RCOG_1l, RCOG_15, RCOG_17; Sands_1; RCM_5, RCM_7, RCM_31, RCM_38, RCM_39, RCM_41; NHSE_8, NHSE_9, NHSE_11; AIMS_2,AIMS_7, AIMS_8; SoR_2, SoR_4, SoR_5, SoR_6, SoR_7, SoR_8, SoR_9, SoR_10, SoR_11, SoR_12	7, 12, 13, 15, 17, 18, 20
Confusion and stress around 'rules'	BR_8, BR_12, BR_14, BR_16, BR_19; RCM_31; AIMS_7	12, 15, 19 20
Unintended Consequences	BR_1, BR_8, BR_12, BR_16, BR_17, BR_23; RCOG/RCM_1e, RCOG/RCM_1j , RCOG/RCM_1k, RCOG/RCM_1l; Sands_1; RCM_2, RCM_7, RCM_8, RCM_11, RCM_16 ; NHSE_1, NHSE_2, , NHSE_11; AIMS_8;	2, 4, 5, 8, 12, 13, 15, 17, 18, 19, 20
Need for flexibility	BR_8, BR_6, BR_15, BR_18; Sands_1; RCM_4, RCM_41; NHSE_8; AIMS_2, AIMS_8; SoR_12	3, 7, 10, 13, 17, 21
Acceptable time for support	BR_1, BR_6, BR_8, BR_16, BR_17, BR_18, BR_23; RCOG_7; Sands_1; RCM_3, RCM_8, RCM_11, RCM_27, RM_28; AIMS_2, AIMS _5, AIMS_8; SoR_2, SoR_4, SoR_5, SoR_9, SoR_10, SoR_11, SoR_12	5, 7, 13, 15, 16, 17, 18, 19, 20, 21, 26
Loss of human rights for gain in infection control	BR_16, BR_17, BR_19, BR_23, BR_8, BR_18; RCOG/RCM_1g , RCOG/RCM_1c, RCOG/RCM_1e, RCOG/RCM_1f , RCOG_9, RCOG_6; RCM_11, RCM_30, RCM_28,RCM_41, RCM_39, RCM-38, RCM_11; NHSE_9, NHSE_10; SoR_2, SoR_6, SoR_5, SoR_8, SoR_7, SoR_4, SoR_9, SoR_10, SoR_11, SoR_12; ISUOG 5, ISUOG_6	3, 5, 11, 14, 17

For peer review only - http://bmjopen.bmj.com/site/about/guidelines.xhtml

Postcode lottery of care

The notion of a postcode lottery of maternity service provision gained traction in the media over the summer of 2020 (16). Concerns were reflected in national documents from almost all included organisations, the stakeholder interviews, and reflected in Trust level responses. Variation was justified by some organisations as a reaction to local need, for example: '*restrictions on other visitors should follow hospital policy and national guidance*' (RCM_7) and 'a*ll staff should work to the same local policy, to provide a consistent service to women'* (SoR_5). The caveat to most guidance was that any policies needed to be re-addressed in the event of local spikes in COVID-19 cases or following local risk assessment, to ensure a '*consistent service to women'* (SoR_2):

> But it's guidance, and every hospital will make its own decisions and to a certain extent, they will have to because the physicality, the layout, the facilities that they have in hospitals will differ. You know, there's not much room in the waiting area if the corridors are very narrow [so] that the people can't have a two-metre distance. (Stakeholder 20, Miscarriage Association)

Some Trusts were identified as having '*gone out of their way to ensure their services remain family-centred'* (BR_14), whereas in others, partners were unable to attend any antenatal appointments or scans (BR_16). NHS England released guidance in September 2020 intended to assist trusts to reintroduce access for partners and visitors (NHSE_8. Some organisational responses claimed that this led to '*some Trusts starting to backtrack and reduce restrictions in maternity services'* (BR_17), while other Trusts continued to impose restrictions (BR_18) and often without a clear rationale for these variations:

> And that's the problem. You… because down the street, down the road, you could get a very warm, empathetic ultrasonographer who says, of course, yeah, I realize how difficult is. You know, it doesn't take much, and we just have lost it because people are stressed and there's lots of reasons for it, but it's just not good enough. (Stakeholder 18, Independent Midwifery Advisor)

Confusion and stress around 'rules'

Concern over a lack of clarity in decision making and changes in policies around companionship were highlighted. For instance, letters from Birthrights to maternity leads (e.g.

For peer review only - http://bmjopen.bmj.com/site/about/guidelines.xhtml

BR_12, BR_16, BR_19) repeatedly emphasised the need for clear reasons, evidence, and justification as to why decisions were being made. Concerns included that Trusts *'acted too quickly to withdraw services'* and *'decision-making has not always been proportionate or transparent'* (BR_18). While most Trusts made some reference to infection risk as the rationale for the restrictions, a sizeable minority (9/25) did not. Many Trusts offered no rationale as to why partners could attend some appointments but not others, e.g.:

> One birth partner may attend for the 20-week anomaly scan only. Women to attend all other scan appointments unaccompanied (South West 2).

Birthrights and some stakeholders highlighted a failure to communicate local restrictions in a timely manner, compounded by rules changing rapidly and difficulties in communicating these changes widely and consistently to large numbers of healthcare professionals. This confusion about the rules at local level was also compound by changeovers of staff and communication between different teams in some areas – *'there's been no consultation with sonographers in terms of risk assessments* […] *or changes in practice'* (Stakeholder 26, The Society of Radiographers), and services being provided by staff from outside the maternity team.

While some stakeholders noted that individual Trusts responded to this confusion by using a range of public communications, data from the 25 maternity trusts found that less than half (10/25) had a consistent message about companionship across different channels. Parent frustration reported by stakeholders also related to how the rules for birth companions and visiting seemed at odds with the social distancing rules outside of the hospital context:

> In the middle of lockdown, it was 'we don't like it, but we know you're keeping us safe'. Now 'it's we don't like it and I don't see how you're keeping us more safe doing this because I can meet my partner in the pub, but he can't come to my scan. I can do this, but I can't do that'. Yes. So, it's more of an angry mood now. (Stakeholder 12, Maternity Voices Partnership)

In some of the documents by Birthrights (e.g. BR_14; BR_16) and RCM (i.e. RCM_31) they called for the harm caused by restricted access by birth partners to be properly and transparently considered within the decision-making processes:

For peer review only - http://bmjopen.bmj.com/site/about/guidelines.xhtml

> We would be grateful if you could publish or send us the risk assessment that quantifies the increased risk of spreading COVID within the unit (despite PPE and other mitigating factors, and the fact that most partners are from the same household) caused by relaxing restrictions, and weighs this against the known harms to pregnant women, birthing people and their families from keeping the current restrictions in place. (BR_16)

Unintended Consequences

Organisations including RCM, RCOG, NHS England and Birthrights highlighted that having trusted companions throughout labour and birth is linked to improved outcomes, and a lack of companionship was associated with increased need for pharmacological or other interventions (AIMS_8, BR_23). This included perceived impacts on the labour process due to, for example, [increased] *'demand for epidurals'* (RCM_8). Alongside the obvious fear and anxiety of infection, organisations and stakeholders highlighted concerns about women feeling '*petrified'* (Stakeholder 15, Professor of Midwifery) or not accessing maternity care, '*due to the women's preferred birth partner not being allowed to accompany her*' (RCM_2). There were also concerns of partners feeling *'unsupported and uncared for'* (Stakeholder 20, Miscarriage Association) due to being unable to be with their partner when they heard bad news (during ultrasound) or missing the birth due to *'being told that they should wait in the car park or something'* (Stakeholder 7, Birthrights). All but four Trust websites contained messages of empathy regarding the restrictions, sometimes alongside expressions of regret and/or justifications for their necessity: '*We understand the restrictions we have had in place over recent months have been particularly hard for pregnant women and their families*' (North West 1). Concerns were also expressed by stakeholders and within documents by Sands and Birthrights, towards women who had experienced prior baby loss or for women for may receive bad news alone during the scan:

> Women are being asked to attend scans alone, with many sharing frustrations that they cannot video link to their partners. These very vulnerable women tell us they are concerned about having to attend stressful antenatal appointments and scans alone. While units are being encouraged to consider facilitating women to take a video clip at the end of an appointment, this is reliant on local policies. (Sands_1)

For peer review only - http://bmjopen.bmj.com/site/about/guidelines.xhtml

Companionship was noted to have practical as well as emotional implications for women. Some of the documents claimed that the absence of companions meant that women required more support from maternity care professionals (e.g. RCOG/RCM_1j; NHSE_1) creating additional stress for over stretched services (RCOG/RCM_1l), and additional potential exposure to COVID infection. Additional workload pressures were also created when staff had to police non-compliant behaviours:

> There have been frustrations. For example, partners now come on to postnatal ward for a fixed time period. And staff have reported things like literally having to find partners from behind curtains. "You need to go now". … So, a lot of stress. Because [having to] explain to people why they can't have what they have or what they want. (Stakeholder 12, Maternity Voices Partnership)

Some practitioner respondents reported that they or their colleagues experienced moral distress when social distancing rules prohibited physical contact with women who were alone, or receiving bad news:

> All of a sudden it was just women on their own for us. And that was really, really stressful for the women and the staff. And a lot of a lot of my job is giving bad news. And to give that to women that are on their own with no support; you can't touch them. You can't hug them. And so that for us is really, really challenging. I think that was probably the most challenging thing. (Stakeholder 24, Midwife Sonographer)

Need for flexibility

Concerns discussed within the documents (e.g. BR_8; BR_18; RCM_4) and raised by stakeholders related to the blanket adoption of visitation rules across whole hospitals. Some commented that pregnant women and childbearing people were a *'separate population with separate needs'* (Stakeholder 7, Birthrights), arguing that visiting rules adopted in other areas of healthcare should not apply to a perinatal population:

> So, you know, several heads of midwifery were saying to me, I want to do this, but they won't let me because they made a decision about what the visiting will look like in this hospital. And they do not see maternity as an exception. And, you know, it is an interesting reflection, isn't it, that maternity has always been a service that has seen

For peer review only - http://bmjopen.bmj.com/site/about/guidelines.xhtml

For peer review only

> itself as an exception to the health care service in which it sits. (Stakeholder 17, NHS England and NHS improvement)

Responses from organisations including AIMS, Birthrights and the RCM argued how there needed to be consideration of women's unique situations. Restrictions on companionship were considered to have a disproportionate impact on those who were facing disadvantages including those for whom English is a second language, women with mental health problems, cognitive impairments, refugee and asylum seeking women (AIMS_8, RCM_4, BR_8, BR_18). Only one Trust included a statement on their website about offering personalised (flexible/individualised) care for all women, that might not be in line with COVID policies. Five others said they offered this on a case-by-case basis (often expressed as 'exceptional circumstances'). One of these referred to Black, Asian, and Minority Ethnic communities along with concerns about greater COVID risks, and three referred to 'allowing' women to bring a companion if they 'needed assistance'. In two Trusts this was explicitly linked to women with disabilities:

> Partners and family members will not be allowed to enter the building unless you need support from a carer/relative (for example, if you have a disability and need support) (North West 2).

Birthrights stipulated how 'protected characteristics' under the Equality Act 2010 (e.g. physical disability or mental health condition) meant that maternity Trusts were obliged to make reasonable adjustments (e.g. BR_15; BR_18). NHS England emphasised the need for 'essential visitors' (seen as different to 'normal visitors') for those with specific communication or care needs (NHSE_8). AIMS also stressed that on some occasions, on a '*case by case basis*', such as a disability, *'a second birth partner may be critical to women's mental wellbeing or other needs'* (AIMS_2).

The lack of flexibility for highly sensitive events such as for those who experienced trauma or loss were also highlighted by Birthrights (e.g. BR_18) and RCM (e.g. RCM_41). While some incidents of positive practice were identified, stakeholders also shared stories of parents whose baby had died in utero being unable to take photographs or spend time with their deceased child:

For peer review only - http://bmjopen.bmj.com/site/about/guidelines.xhtml

> We had a lot of stories from parents who hadn't been allowed to take photographs, haven't had time to sit and hold their baby. And I think all of those were linked to both a lack of space, to lack of bereavement space, but also a lack of staff understanding of how to adapt bereavement care standards. We also saw in this group a lot of problems around not having the partner with them. (Stakeholder 10, Sands)

'Acceptable' time for support

Access to and timing of support was an issue at key stages during the perinatal journey, and notably during antenatal ultrasound appointments and during early onset of labour.

A key area of contention related to women having to attend ultrasound scans unaccompanied: a situation described by one of the stakeholders as *'ludicrous'* (Stakeholder 7. Birthrights). RCOG guidance recommended that *'patients should be asked to attend alone if possible or with a maximum of one partner/visitor'* (RCOG_5), whereas a RCM document stated '*partners should attend scans unless rooms are too small to socially distance: partners may attend scans virtually'* (RCM_28). However, in contrast to this permission for virtual contact, a joint statement by SoR, RCOG, RCM and the British Medical Ultrasound Society stated that devices required for remote contact by partners via video/phones are a vehicle for transmission (due to surface contamination), and that recordings would impact on scan time, sonographer concentration and potential detection of fetal abnormalities – although it was acceptable for the mother (if in line with local policies) to "*save a short 10–30 second cine clip of the fetus at the end of selected examinations"* (SoR_11).

Trust data revealed that while most permitted partners at one or both standard ultrasound appointments (12 and 20 weeks), seven (~30%) did not. Four Trusts mentioned video or other means of 'virtual' companionship, but usually to specify that videos of scans were not permitted. Only one Trust referred to women being able to phone a partner for support if the sonographer were to find "*important clinical information that your partner needs to be aware of*" (Greater London 2). While many stakeholders were critical of the ultrasound restrictions on companionship, one respondent argued the need to highlight that antenatal ultrasound scanning continued, even when *'other screening programmes went into hibernation'* (Stakeholder 26, The Society of Radiographers). Some defended restrictions on companionship during scanning, noting that scans often have to take place in areas *'like a broom cupboard in a very small poorly ventilated space'* (Stakeholder 17, NHS England and NHS improvement)

For peer review only - http://bmjopen.bmj.com/site/about/guidelines.xhtml

coupled with the restricted time to undertake the examination and sterilising the room and equipment after each appointment. One reported that there had been a *'downgrading'* of the importance of scans as a medical examination that required focused concentration in challenging situations, during vociferous debate about partner attendance (Stakeholder 26, The Society of Radiographers). However, while sonographers may have faced increased risks due to screening large numbers of women, the specific rationale for not allowing videos as an alternative was challenged:

> You can argue the toss as to whether some of the justifications for not allowing that were real or weren't real. You know, is there really a risk of infection if you pick up your phone? Really? Maybe some anxiety for sonographers or whoever's doing the scan. You know, you don't really want the phone on with a video while you're doing the scan because who knows, they might use it in some kind of litigation. Who knows? But whatever it was, it really didn't help. (Stakeholder 20, Miscarriage Association)

A further area of contention concerned companionship during labour and birth. While organisations such as Birthrights argued for companionship throughout, less than half (9/25) the Trusts referred to partners attending '*throughout*' or '*for the duration*'. Three Trusts referred to companionship being permissible only when the woman was in '*established*' or '*active*' labour (with no details as to how this would be established); and 13 Trusts did not specify the relevant phase of labour. RCM guidance advised that women would not be able to have partners present during inductions that took place in a bay or ward (RCM_27). Only six Trusts (25%) indicated that partners could be present during induction of labour, and four allowed partners, but with limitations (either restricted to daytime or if the woman needed additional support). About half (12) provided no information, and three explicitly stated that partners were not allowed:

> If you are attending for induction of labour please attend alone, your birth partner will join you once you are transferred to the Delivery Suite. (Yorkshire and Humber 1)

Birthrights and AIMS (i.e. BR_8; AIMS_5; BR_18; BR_23) also raised concerns about cervical dilatation as the only acceptable indicator of active labour, meaning that some women who may not have wanted (or needed) a vaginal examination felt pressured to accept the procedure if they wanted their birth companion to be granted access to the labour ward.

For peer review only - http://bmjopen.bmj.com/site/about/guidelines.xhtml

There were examples of innovation to try to support companionship for women and access for their partners. Some Trusts initiated or extended the provision of labour induction in community settings or in private hospital rooms (rather than multi-occupancy early labour wards) to prevent separation of women from birth partners (RCM_8; RCM_27). Some stakeholders also referred to more flexible approaches to induction such as partners being able to *'come and settle them* [women] *in'* and to use *'Facetime to be with their partner all the time'* (Stakeholder 21, Midwife with a national role).

Loss of human rights for gain in infection control

There was some evidence from stakeholders that hospital decision makers in some settings believed that companionship during the maternity episode should not be prioritised over other areas where attendance of close family members would usually be seen as a critical human need and right: especially when someone was dying in hospital:

> So, it was interesting and when I would speak to the head of midwifery, sometimes it felt like they were saying, you know, well, everyone's got to make….sacrifices. And there are people dying alone in hospital. There are people suffering terribly alone in hospital, unable to have visitors…. [while] there were women saying, you know, it's my right to have a companion it's your job to provide care for me. So, it felt at times like each group with their own concerns was unable to think about or found it difficult to take on board the concerns of the other group. (Stakeholder 17, NHS England and NHS improvement)

The underlying principle within most of the guidance reviewed was that 'safety', was primarily conceptualised as the prevention of transmission of infection, both to service-users and to staff. NHS England documents referred to minimising '*control risks working with your IPC* [Infection, Prevention & Control] *leads, whilst still allowing the maximum possible safe access'* (i.e. NHSE_9). The RCOG/RCM also noted the need to minimise the number of attendees (as ISUOG_5 quote above), but acknowledged that one person could be there for antenatal visits should a woman choose this:

> You will be asked to come alone to clinical appointments or keep the number of people with you to one (including midwifery visits in your home). This will include being

For peer review only - http://bmjopen.bmj.com/site/about/guidelines.xhtml

> asked not to bring your children with you to appointments. This is important to protect maternity staff, other women, and babies, and you and your family from the risk of infection. (RCOG/RCM_1g)

Birthrights was one of the key organisations to recommend that notions of safety might also include emotional and psychosocial risks of women being unattended (e.g. BR_17, BR_16, BR_19, BR_18) - '*The damage caused by ongoing restrictions needs to be weighed up against the requirements of infection control'* (BR_19). Several of their documents (e.g. BR_18; BR_19) claimed that routinely restricting birth companions was a violation of women's (and partners) human rights. Despite this, stakeholders stated that, in practice, women's human rights and choices around companionship did not feature as part of the decision-making processes:

> And I think we've spent the last, you know, however many years banging on about the fact we want to give women choice and rights and sharing that discourse and encouraging women to become empowered. And then COVID comes along and we just say, no, no, we're not doing that (Stakeholder 17, NHS England and NHS improvement)

The RCM (RCM_41) stated that their '*greatest concern'* was '*safety being sacrificed in favour of popularity'* which seemed to imply that companionship should not outweigh the need to prevent infection of its *'members'* and of '*women and families'*. SoR also highlighted that its guidance had *'risk assessments'* at its core (SoR6). The guidance did not preclude 'people being accompanied', but that it '*must only happen if the safety of the patient and sonographer is not compromised*' (SoR_8). However, others argued that day to day decision making was based around a belief about safety that was limited: '*because it's not just about the physical self, it's about psychological self*' (Stakeholder 14 Senior Midwife Leader):

> But I think safety generally is an interesting thing because….You know, so many different things affect safety don't they, so something like being able to have your partner with you might not be seen as a primary thing affecting safety in comparison with protecting against COVID, but actually if it impacts on someone's mental health in either the partner or the mother, that does have an effect on safety (Stakeholder 5, National Maternity Voices Partnership)

For peer review only - http://bmjopen.bmj.com/site/about/guidelines.xhtml

DISCUSSION

In this paper we have drawn on guidance from national statutory and service-user organisations, key stakeholders, and public facing Trust-level data to consider the organisational issues associated with companionship in maternity care during the COVID-19 pandemic. The value of companionship during labour and birth for women and birthing people is widely recognised, in terms of clinical benefits, and short and longer term psychosocial impacts (1, 9). As evidenced within this paper, during the COVID-19 pandemic, at the policy and organisational level, assumptions and norms about companionship, accompaniment and visiting during facility-based health care provision have faced profound challenge. Others have noted the variance in maternity organisation response during the pandemic(17). Some variation can probably be explained by changing national knowledge about the prevalence and impacts of COVID-19, and by different levels of exposure to COVID-19 infection. However, our data suggest that this was not the case where blanket policies were applied with minimal individual flexibility, or where there was unjustified variation in visiting and companionship rules, coupled with poor and inconsistent communication.

We found particular concern about lack of access to companionship in two distinct areas. First women being unable to have any communication (actual or virtual) with partners or other family members at ultrasound scan; and denial of intrapartum companionship until labour was 'established'. In relation to the former case, there is some evidence that, beyond the emotional and psychological benefits for the mother, when fathers and partners are present for antenatal ultrasound scan, there are significant effects on their identification with the fetus (as their future child) and their empathic relating with the mother(18, 19). This implies that being present for ultrasound scans has important public health and relationship benefits for them, and their partner and baby. In the latter case, in some Trusts, ensuring that labour had progressed sufficiently was perceived by some stakeholders to be associated with coercive and invasive practices, such as regular vaginal examinations when women may otherwise not have needed or wanted such examinations. General uncertainty over organisational companionship permissions may also be reflected in anecdotal rises in women choosing to freebirth(20, 21), and the issuing of associated RCM guidance to ensure appropriate professional responses(22). Trust policies that restricted companionship until labour was established (or until birth was imminent) seemed to be built on an assumption that companionship was only really needed when labour was very intense, and/or when the birth was imminent so that the birth companion (as the co-parent) could be 'permitted' to witness to the birth of their baby. In contrast, other

For peer review only - http://bmjopen.bmj.com/site/about/guidelines.xhtml

Trusts seemed to recognise, at the organisational level, that active and engaged birth companionship throughout labour (from the early stages of spontaneous labour, or from the time of labour induction through to the birth) is a mechanism for clinical, psychological and emotional safety for the mother, partner and child, both in the short term, and, critically, in the longer term, when the threat of COVID-19 infection is long over (11, 23).

The pandemic brings into sharp focus the fundamental and underpinning ethical dilemma between social actions that ensure the greatest benefit for the population as a whole, and the individual human rights of each person within that population(24). Resolving this potential conflict of ethical imperatives depends on an open and informed debate about rights and consequences. In terms of maternity care, this requires a sophisticated understanding of what 'companionship' means, over the whole life course, and for mother, baby, and family. It also requires attention to the potential moral distress of maternity care staff (and health care staff in general, including ultrasonographers) who are faced with the stress of having to balance these two imperatives with real people, in intensely emotional real time, repeatedly day in and day out, and at times with insufficient PPE equipment available; at a time when they, too could be pregnant women at risk of exposure to infection, or parents/carers fearful of infecting family members(25-28). The study by Nguyen and colleagues identified that that infection risks for acquiring infection were high for staff compared to women, and particularly among front-line health-care workers(29).

This is the first study to bring together national policy and stakeholder views with Trust based public facing data to understand how maternity care has been organised in England during COVID-19. Although we cannot be sure we captured every single relevant document produced over the period of our data collection, triangulation across data sources and across multiple researchers enabled rich insights into how and why variations occurred, and the perceived impacts. The pragmatic restriction of the Trust level data collection to only 25 Trusts (10% of maternity care providers in the UK), and the restriction to maternity-specific documents and guidance may be seen as a limitation, the organisations that were included were selected purposively to reflect a wide range of relevant characteristics. Since this paper is focused on policy and organisational responses to the pandemic, service-users, birth companions and healthcare professionals were not included. In addition, our analysis did not include findings related to postnatal care, or care in neonatal units. These areas, and the unintended (positive and negative) short- and longer-term consequences of different interpretations of the value of

For peer review only - http://bmjopen.bmj.com/site/about/guidelines.xhtml

companionship when balanced against infection control, are critical areas for examination during the on-going COVID-19 crisis. Future outputs from the ASPIRE project will address these gaps.

CONCLUSION

This paper presents insights from the ASPIRE COVID-19 UK study to understand how companionship in maternity care was operationalised at the organisational level in antenatal and intrapartum care during COVID-19. Our findings illustrate variations in policy at national and local level, coupled with poor and inconsistent communication of how the restrictions changed in some sites, and a lack of clarity in the decision-making processes. The evidence highlighted a lack of flexibility in responding to women with more complex needs in some cases, the negative and positive unintended consequences of companionship restrictions, and the challenges of conceptualising and balancing infection risk and emotional and psychological distress. However, there was evidence that creative solutions were possible, since, despite significant pressures, some Trusts appeared to continue to provide full companionship.

Overall, these concerns illustrate something much more fundamental than merely barriers to hospital 'visiting. While the NHS England Better Births policy agenda highlights the need for safety and personalisation within maternity care, these findings suggest that, over the time period captured by this study, personalisation (and emotional and psychological safety) became sacrificed in some (but not all) situations to the overriding imperative to minimise infection spread with high emotional and psychological costs. Further research should capture the views and experiences of healthcare professionals and service-users, and clinical outcome data from different settings. There is an urgent need to determine how to balance risks and benefits sensitively and flexibly and to create optimum outcomes for women, pregnant people, fathers, parents, infants, families and staff, during the current and future crisis situations.

ACKNOWLEDGEMENTS

A funding statement: This research is funded by the Economic and Social Research Council (ESRC), as part of UK Research and Innovation's rapid response to COVID-19 [grant number ES/V004581/1]. Full details of the main study are available via ResearchRegistry (researchregistry5911) and via UKRI Gateway (https://gtr.ukri.org/projects?ref=ES%2FV004581%2F1)

For peer review only - http://bmjopen.bmj.com/site/about/guidelines.xhtml

Author contributions: SD with input from the ASPIRE-COVID19 Collaborative Group designed the study. RN, GT, SD and NC interviewed the stakeholders; MCB, GM and JC were involved in data extraction for the documentary analysis; SH, AS and NC collected and analysed Trust level data. GT, MC and RN were involved in developing themes from the interview and documentary data, and GT synthesised the Trust level data into the data set. Final themes were agreed with all authors. All authors and the ASPIRE-COVID19 Collaborative Group contributed to writing and reviewing the manuscript.

A data sharing statement: Trust level data is included in the paper. Details of all documents analysed is provided/with all information freely available. All relevant interview data concerning companionship will be openly available from UCLanData and a doi provided on paper acceptance.

REFERENCES

1. Bohren MA, Berger BO, Munthe-Kaas H, Tunçalp Ö. Perceptions and experiences of labour companionship: a qualitative evidence synthesis. *Cochrane Database Syst Rev* 2019;3, doi: 10.1002/14651858.CD012449.pub2

2. Jordan B. Birth in four cultures: A crosscultural investigation of childbirth in Yucatan, Holland, Sweden, and the United States: Waveland Press; 1992.

3. Tani F, Castagna V. Maternal social support, quality of birth experience, and post-partum depression in primiparous women. *J Matern Fetal Neonatal Med* 2017;30(6):689-92.

4. Withers M, Kharazmi N, Lim E. Traditional beliefs and practices in pregnancy, childbirth and postpartum: A review of the evidence from Asian countries. Midwifery. 2018;56:158-70.

5. Draper J. Whose welfare in the labour room? A discussion of the increasing trend of fathers' birth attendance. Midwifery. 1997;13(3):132-8.

6. Jackson SH, Schlichting FR, Hulme RL. Effect of fathers at cesarean birth on postpartum infection rates. AORN 1982;36(6):973-7.

7. Stephan G, Silbermann B. Father in delivery room wins decision that could land you in court. Legal aspects of medical practice. 1979;7(7):49-50.

8. Kabakian-Khasholian T, Portela A. Companion of choice at birth: factors affecting implementation. BMC Preg Childbirth. 2017;17(1):1-13.

9. World Health Organization. Companion of choice during labour and childbirth for improved quality of care: evidence-to-action brief. World Health Organization; 2016.

For peer review only - http://bmjopen.bmj.com/site/about/guidelines.xhtml

10. Pestvenidze E, Bohrer M. Finally, daddies in the delivery room: Parents' education in Georgia. *Global Public Health*. 2007;2(2):169-83.

11. Coulter A, Richards T. Care during covid-19 must be humane and person centred.*BMJ* 2020;370:m348

12. Ferber A. How Covid-19 led to a global crisis in maternity care. New Statesman. 17 December 2020 How Covid-19 led to a global crisis in maternity care (newstatesman.com)

13. Black BL, J; McKay, G. Birth partners are not a luxury
BMJ 24 September 2020 https://blogs.bmj.com/bmj/2020/09/24/birth-partners-are-not-a-luxury/

14. Gale NK, Heath G, Cameron E, Rashid S, Redwood S. Using the framework method for the analysis of qualitative data in multi-disciplinary health research. *BMC Med Res Methodol* 2013;13(1):1-8.

15. Vaismoradi M, Turunen H, Bondas T. Content analysis and thematic analysis: Implications for conducting a qualitative descriptive study. *Nurs Health Sci* 2013;15(3):398-405.

16. Iacobucci G. Partners' access to scans and birth is a postcode lottery, data show. *BMJ* 2020;371:m3876

17. Jardine J, Relph S, Magee LA, von Dadelszen P, Morris E, Ross-Davie M, et al. Maternity services in the UK during the coronavirus disease 2019 pandemic: a national survey of modifications to standard care. BJOG. 2021;28(5):880-889.
doi: 10.1111/1471-0528.16547.

18. Walsh TB, Davis RN, Palladino CL, Romero VC, Vijay Singh M. Moving up the" magic moment": Fathers' experience of prenatal ultrasound. *Fathering* 2014;12(1):18.

19. Draper J. 'It's the first scientific evidence': men's experience of pregnancy confirmation. *J Adv Nurs* 2002;39(6):563-70.

20. Dahlen H. During COVID-19, women are opting for 'freebirthing' if homebirths aren't available. And that's a worry: The Conversation 2020 9 July 2020 https://theconversation.com/during-covid-19-women-are-opting-for-freebirthing-if-homebirths-arent-available-and-thats-a-worry-142261.

21. Greenfield M, Payne-Gifford S, McKenzie G. Between a rock and a hard place: Considering "freebirth" during Covid-19. *Frontiers in Global Women's Health*. 2021;2:5 https://doi.org/10.3389/fgwh.2021.603744.

For peer review only - http://bmjopen.bmj.com/site/about/guidelines.xhtml

22. Royal College of Midwives. Clinical Briefing Sheet: ‘freebirth’ or ‘unassisted childbirth’ 30 April 2020 https://www.rcm.org.uk/media/3904/freebirth_draft_23-april-v5-002-mrd-1.pdf.

23. Lavender T, Downe S, Renfrew M, et al. Rapid analytic review: Labour and birth companionship in a pandemic: Companionship of choice for asymptomatic childbearing women in hospital throughout labour and birth. *Royal College of Midwives* https://www.rcm.org.uk/media/3951/birth-companionship-in-a-pandemic-master-27-04-2020-002.pdf (Accessed 23 February 2021)

24. Xafis V, Schaefer GO, Labude MK, Zhu Y, Hsu LY. The perfect moral storm: Diverse ethical considerations in the COVID-19 pandemic. *Asian Bioeth Rev* 2020;12:65-83.

25. Hunter B, Renfrew MJ, Downe S, et al. Supporting the emotional wellbeing of midwives in a pandemic: Guidance for RCM. 2020. Royal College of Midwives. https://www.rcm.org.uk/media/4095/rcm-supporting-the-emotional-wellbeing-of-midwives-during-a-pandemic-v1-submitted-to-rcm_mrd.pdfm (Accessed 21 February 2021)

26. Jeffrey DI. Relational ethical approaches to the COVID-19 pandemic. *J Medical Ethics* 2020;46(8):495-8.

27. Sheather J, Fidler H. Covid-19 has amplified moral distress in medicine. *BMJ* 2021;372:n28

28. Chew C, Ko D. Medical ethics in the era of COVID-19: Now and the future. *Respirology* https://doi.org/10.1111/resp.13927 (Accessed 5 January 2021)

29. Nguyen LH, Drew DA, Graham MS, et al. Risk of COVID-19 among front-line health-care workers and the general community: a prospective cohort study. *Lancet Public Health* 2020;5(9):e475-e83.

For peer review only

For peer review only

For peer review only - http://bmjopen.bmj.com/site/about/guidelines.xhtml

Supplementary File 1

Interview schedule

Opening question

In your view, what are the most important issues for maternity care provision that have come out of COVID-19?
Probe: *Are these views based on any particularly memorable experiences?*

Changes/adaptions to service delivery

How have [i.e. maternity, midwifery, obstetric, neonatal, ambulance delivery, staffing, any other?] services been adapted during COVID-19 pandemic?
Prompts: *How significant or minor have these changes been?*
Can you describe some of the primary Covid-19 interventions?
Have these adaptions varied by place and/or time? If so, what do you think explains this variation?

Decision making processes

From your perspective, who makes decisions about the changes?

Prompts: *How are these decisions made?*
Were decisions made at a multidisciplinary level or separately?
How did the different agencies – work together - who had overall responsibility?
Who has been consulted about the changes – are all those impacted consulted (e.g. at UK level were MVPs consulted and/or for neonatal were the local PAG group consulted?)
If there is resistance, how is that dealt with?

How have safety principles (for service-users and staff) informed decision-making/practice changes?

How have personalised care principles informed decision-making/practice changes?
Prompts: *What personalised care aspects have been considered?*

To what extent has the needs and care of BAME/vulnerable populations been considered in the decision-making processes?

What other factors have influenced decision-making/changes in care provision?

Communication and implementation

How have the recommended practices and guidance been developed?
Prompts: *How effective have these methods been?*
How was conflict in what to include resolved?

For peer review only

How have they been communicated to practitioners/front line staff?

Prompts: *How effective have these methods been?*
What more is needed?

How is service delivery being monitored over this period?

Prompts: *What feedback loops are in place*?
How is this information used?

Impact

What has been the (positive and negative) impact of the changes/adaptions on:

- Staff
- Parents and families (siblings/grandparents)
- Service delivery
- Data collection and sharing (i.e. Maternity Services Dashboard and Better Births Minimum Data set)

In which data do you think the effects of the pandemic and the adaptations made to service provision can be seen? Can we have access to the data?

Barriers and facilitators

What has worked well in providing safe and personalised maternity care during the pandemic?

Prompts: *Do any particular Trusts/services/lobby groups and etc stand out for you?*
Why is this?

What have been the challenges or barriers in providing safe maternity care?

What have been the challenges or barriers in providing personalised maternity care?

Recommendations and Sustainability

What else is needed to ensure safe and personalised maternity standards and principles are upheld in:

- future routine maternity care
- a future crisis/pandemic

If there was one thing that you would change about how maternity care was managed what that would be?

What innovations been implemented that could be opportunities for how maternity care could be organized in the future?

Prompts: *Can you identify where these innovations and opportunities are happening at the moment?*

How do you think services should prepare for future occurrences/pandemics?

Is there a specific Trust/Health Board you think we should include in our case studies?

For peer review only

Prompts: *If so, can you tell us why you think this is a particularly relevant site?*

Are there other things, that have not been mentioned that you would like to share?

Is there anyone else that you think we should talk to?

For peer review only

Organisation/Code	Name of Document/Link	Date of documen
**AIMS**		
AIMS_1	Coronavirus and the Maternity Services on AIMS website https://www.aims.org.uk/campaigning/item/coronaviru	25/03/2020
AIMS _2	AIMS Response to the Clinical guide for the temporary reorganisation of intrapartum maternity care during the coronavirus pandemic, version 1, published 9/4/20 https://www.aims.org.uk/assets/media/444/aimscomments2020-04-11.pdf	09/04/2020
AIMS_5	Submission from AIMS to the Health and Social Care Committee Inquiry: Delivering Core NHS and Care Services during the Pandemic and Beyond<br>https://www.aims.org.uk/assets/media/447/aimssubmissionhealthandsocialcarecommittee2020-05.pdf	07/05/2020
AIMS_7	AIMS comments on latest guidance on birth partner restrictions https://www.aims.org.uk/campaigning/item/latest-guidance-birth-partner	25/09/2020
AIMS_6	Press Release: Almost Half of NHS Trusts Force Pregnant Women to Attend Vital Maternity Services Alone https://www.aims.org.uk/campaigning/item/trusts-force-pregnant-women-to-attend-alone	12/10/2020
AIMS_9	AIMS calls for clearer guidance from NHS England on lifting partner restrictions in maternity<br>https://www.aims.org.uk/campaigning/item/nhse-letter	21/11/2020
AIMS_8	AIMS asks Society and College of Radiographers to review guidance on partners and supporters at scans https://www.aims.org.uk/campaigning/item/scor-letter	12/11/2020
**Birthrights**		
BR_1	Human rights charity calls for protection of UK women in childbirth during national emergency https://www.birthrights.org.uk/wp-content/uploads/2020/03/Final-Covid-19-Birthrights-31.3.20.pdf	31/03/2020
BR_8	Coronavirus and the impact on people with protected characteristics https://www.birthrights.org.uk/wp-content/uploads/2020/05/Birthrights-Covid-19-Maternity-Briefing-women-and-equalities-committee-final.pdf	01/04/2020
BR_6	Final letter SOR-BMUS 22nd April Birthrights joint letter with National Maternity Voices, Sands, the Twins Trust and ARC https://www.birthrights.org.uk/wp-content/uploads/2020/04/Final-letter-SoR-BMUS-22-April.pdf	22/04/2020
BR_12	Letter to NHSE regarding ongoing visiting restrictions https://www.birthrights.org.uk/wp-content/uploads/2020/08/Letter-to-NHSE-re-ongoing-visiting-restrictions.pdf	20/08/2020

For peer review only

BR_14	Ongoing visitor restrictions in maternity services https://www.birthrights.org.uk/2020/09/14/ongoing-visitor-restrictions-in-maternity-services/	14/09/2020
BR_15	Template letter for ongoing visiting restrictions in maternity services https://www.birthrights.org.uk/template-letters/	14/09/2020
BR_16	Letter on restricting visitors – Sheffield teaching hospitals https://www.birthrights.org.uk/wp-content/uploads/2020/10/Letter-to-Sheffield-15th-October.pdf	15/10/2020
BR_17	"Partners are not visitors to maternity services" says Birthrights and partner organisations [joint letter on visiting restrictions to NHSE&I] https://www.birthrights.org.uk/wp-content/uploads/2020/10/Final-letter-to-NHSE-from-coalition-re-visiting-restrictions.pdf	20/10/2020
BR_19	Human rights charity calls for easing of visiting restrictions in maternity services press release https://www.birthrights.org.uk/wp-content/uploads/2020/08/Easing-of-visitor-restrictions-press-release-20-August-2020.pdf	20/08/202
BR_18	Human rights implications of changes to maternity services during the Covid-19 pandemic Birthrights briefing, July 2020 https://www.birthrights.org.uk/wp-content/uploads/2020/07/Birthrights-Covid-19-Human-Rights-Committee-Briefing-July-2020.pdf	Jul-20
BR_23	Long term impact of visiting restrictions "could be catastrophic" MPs, academics and campaigners warn NHS England CEO… https://www.birthrights.org.uk/2020/11/15/long-term-impact-of-visiting-restrictions-could-be-catastrophic-mps-academics-and-campaigners-warn-nhs-england-ceo/	15/11/2020
**ISUOG**		
ISUOG_6	ISUOG Safety Committee Position Statement: safe performance of obstetric and gynaecological scans and equipment cleaning in the context of COVID-19 https://www.isuog.org/resource/isuog-safety-committee-position-statement-safe-performance-of-obstetric-and-gynecological-scans-and-equipment-cleaning-in-the-context-of-covid-19.html	23/03/2020
ISUOG_5	ISUOG Consensus Statement on organization of routine and specialist obstetric ultrasound services in the context of COVID-19 https://www.isuog.org/resource/wiley-isuog-consensus-statement-on-organization-of-routine-and-specialist-obstetric-ultrasound-services-in-the-context-of-covid-19.html	31/03/2020

For peer review only

ISUOG_3	ISUOG Interim Guidance (COVID-19) during pregnancy & puerperium: Information for healthcare professionals – an update https://obgyn.onlinelibrary.wiley.com/doi/full/10.1002/uog.22061	01/05/2020
**NHS England**		
NHSE_1	Clinical guide for the temporary reorganisation of intrapartum maternity care during the coronavirus pandemic<br>https://madeinheene.hee.nhs.uk/Portals/0/Clinical%20guide%20for%20the%20temporary%20reorganisation<br>%20of%20intrapartum%20maternity%20care.pdf	09/04/2020
NHSE_2	Delivering midwifery intrapartum care where local COVID-19 escalation protocols are required to be enacted<br>https://www.nice.org.uk/media/default/about/covid-19/specialty-guides/delivering-midwifery-intrapartum-care-where-local-covid-19-escalation-protocols-are-required-to-be-enact.pdf	20/06/2020
NHSE_8	Framework to assist NHS trusts to reintroduce access for partners, visitors and other supporters of pregnant women in English maternity services http://allcatsrgrey.org.uk/wp/download/midwifery/par001599-framework-for-the-reintroduction-of-visitors-throughout-maternity-services-sep-2020.pdf	08/09/2020
NHSE_9	Letter to directors of nursing and heads of midwifery<br>(No longer available on website, available on request)	19/09/2020
NHSE_10	Lateral flow antigen test for pregnant women and support people: Frequently asked questions<br>https://www.england.nhs.uk/coronavirus/wp-content/uploads/sites/52/2020/12/C0972-lateral-flow-antigen-tests-for-maternity-faqs-december-2020.pdf	21/12/2020
NHSE_11	Supporting pregnant women using maternity services during the coronavirus pandemic: Actions for NHS providers Version 1, 14 December 2020<br>https://www.england.nhs.uk/coronavirus/wp-content/uploads/sites/52/2020/12/C0961-Supporting-pregnant-women-using-maternity-services-during-the-coronavirus-pandemic-actions-for-NHS-provi.pdf?fbclid=IwAR0w9hbJSbH1gYYPVhGtE8zRAvwd8PidPkPsEaDnhxzlSumFl7t6byshst8	14/12/2020
**RCOG**		
RCOG/RCM_1c	Coronavirus (COVID-19) Infection in Pregnancy Information for healthcare professionals Version 3<br>https://www.rcm.org.uk/media/3799/coronavirus-covid-19-infection-in-pregnancy-v3-20-03-18.pdf	18/03/2020
RCOG/RCM_1d	Coronavirus (COVID-19) Infection in Pregnancy Information for healthcare professionals Version 4<br>https://www.rcm.org.uk/media/3800/2020-03-21-covid19-pregnancy-guidance.pdf	21/03/2020

For peer review only - http://bmjopen.bmj.com/site/about/guidelines.xhtml

RCOG/RCM_1e	Coronavirus (COVID-19) Infection in Pregnancy Information for healthcare professionals Version 5<br>https://www.rcm.org.uk/media/3824/2020-03-28-covid19-pregnancy-guidance.pdf	28-03-202
RCOG/RCM_1f	Coronavirus (COVID-19) Infection in Pregnancy Information for healthcare professionals Version 6<br>http://www.e-lactancia.org/media/papers/2020-04-03-coronavirus-covid-19-infection-in-pregnancy.pdf	03/04/2020
RCOG/RCM_1g	Coronavirus (COVID-19) Infection in Pregnancy Information for healthcare professionals Version 7<br>https://www.sbmfc.org.br/wp-content/uploads/2020/04/2020-04-09-coronavirus-covid-19-infection-in-pregnancy.pdf	09/04/2020
RCOG_9	Guidance for rationalising early pregnancy services in the evolving coronavirus (COVID-19) pandemic<br>https://www.rcog.org.uk/globalassets/documents/guidelines/2020-05-15-guidance-for-rationalising-early-pregnancy-services-in-the-evolving-coronavirus-covid-19-pandemic.pdf	15/05/2020
RCOG_11	Framework for staffing of obstetrics and gynaecology units during the COVID-19 pandemic<br>https://www.rcog.org.uk/globalassets/documents/guidelines/2020-05-22-framework-for-staffing-of-obstetrics-and-gynaecology-units-during-the-covid-19-pandemic.pdf	22/05/2020
RCOG_6	Principles for the testing and triage of women seeking maternity care in hospital settings, during the COVID-19 pandemic<br>https://www.rcog.org.uk/globalassets/documents/guidelines/2020-05-29-principles-for-the-testing-and-triage-of-women-seeking-maternity-care-in-hospital-settings-during-the-covid-19-pandemic.pdf	29/05/2020
RCOG/RCM_1j	Coronavirus (COVID-19) Infection in Pregnancy Information for healthcare professionals Version 10 https://www.rcm.org.uk/media/4113/2020-06-04-coronavirus-covid-19-infection-in-pregnancy.pdf	04/06/2020
RCOG_7b	Guidance for antenatal and postnatal services in the evolving coronavirus (COVID-19) pandemic<br>https://www.rcog.org.uk/globalassets/documents/guidelines/2020-07-10-guidance-for-antenatal-and-postnatal.pdf	10/07/2020
RCOG_14	Restoration and Recovery: Priorities for Obstetrics and Gynaecology Version 2.1<br>https://www.rcog.org.uk/globalassets/documents/guidelines/2020-06-26-restoration-and-recovery---priorities-for-obstetrics-and-gynaecology.pdf	26/06/2020
RCOG/RCM_1k	Coronavirus (COVID-19) infection in pregnancy Information for healthcare professionals Version 11 https://www.rcog.org.uk/globalassets/documents/guidelines/2020-07-24-coronavirus-covid-19-infection-in-pregnancy.pdf	24/07/2020

For peer review only

RCOG_2	Guidance for provision of midwife-led settings and home birth in the evolving coronavirus (COVID-19) pandemic Version 1.3<br>https://www.rcog.org.uk/globalassets/documents/guidelines/2020-07-10-guidance-for-provision-of-midwife-led.pdf	10/07/2020
RCOG_4	Guidance for fetal medicine units (FMUs) in the evolving coronavirus (COVID-19) pandemic<br>https://www.rcog.org.uk/globalassets/documents/guidelines/2020-11-23-guidance-for-fetal-medicine-units.pdf	10/07/2020
RCOG_5	Guidance for antenatal screening and ultrasound in pregnancy in the evolving coronavirus (COVID-19) pandemic<br>https://www.rcog.org.uk/globalassets/documents/guidelines/2020-07-10-guidance-for-antenatal-screening.pdf	10/07/2020
RCOG_15	Joint RCOG & RCM Statement - Planning for Winter 2020/21: reducing the impact of COVID-19 on maternity services in the UK<br>https://www.rcog.org.uk/globalassets/documents/guidelines/2020-10-08-rcog_rcm_winter_secondwave_statement.pdf	08/10/2020
RCOG_7	Guidance for antenatal and postnatal services in the evolving coronavirus (COVID-19) pandemic<br>Version 3<br>https://www.rcog.org.uk/globalassets/documents/guidelines/2020-10-21-guidance-for-antenatal-and-postnatal-services-in-the-evolving-coronavirus-covid-19-pandemic-v3.pdf	21/10/2020
RCOG/RCM_1	Coronavirus (COVID-19) infection in pregnancy Information for healthcare professionals Version 12 https://www.rcm.org.uk/media/4383/2020-10-14-coronavirus-covid-19-infection-in-pregnancy-v12.pdf	14/10/2020
RCOG_17	RCOG responds to revised NHS coronavirus guidelines https://www.rcog.org.uk/en/news/pregnant-women-allowed-partner-support-at-all-times-in-updated-nhs-guidelines/	16/12/2020
**RCM**		
RCM_8	Induction of labour in a pandemic: A rapid analytic scoping review https://www.rcm.org.uk/media/3924/professional-clinical-briefing-no-7-intrapartum-care-with-symptomsmrd010520.pdf	04/04/2020
RCM_2	Optimising maternity services and maternal and newborn outcomes in a pandemic. A rapid analytic scoping review https://www.rcm.org.uk/media/3869/rapid-review-optimising-maternity-services-for-rcm-v4-8-april.pdf	08/04/2020

RCM_15	Guidance for midwives, student midwives and maternity support workers providing community-based care during the Covid-19 pandemic https://www.rcm.org.uk/media/3900/home-visit-guidance-for-midwives.pdf	09/04/2020
RCM_11	Rapid Analytic Review: Labour and Birth Companionship in a pandemic https://www.rcm.org.uk/media/3951/birth-companionship-in-a-pandemic-master-27-04-2020-002.pdf	27/04/2020
RCM_12	RCM Clinical Briefing Sheet: 'freebirth' or 'unassisted childbirth' during the COVID-19 pandemic https://www.rcm.org.uk/media/3923/freebirth_draft_30-april-v2.pdf	30/04/2020
RCM_16	Optimising mother-baby contact and infant feeding in a pandemic. Rapid analytic review https://www.rcm.org.uk/media/4096/optimising-infant-feeding-and-contact-rapid-review-19th-may-2020-submitted.pdf	19/05/2020
RCM_9	Face-coverings and care in labour for all women https://www.rcm.org.uk/media/4151/clinical-briefing-face-mask.pdf	01/07/2020
RCM_5	Antenatal Care for women without suspected or confirmed COVID-19 and living in a symptom free household https://www.rcm.org.uk/media/4180/clinical-guidance-briefing-one-antenatal-care-for-women-without-symptomsfinalv4-1.pdf	17/07/2020
RCM_4	RCM Briefing on Re-introduction of visitors to Maternity Units across the UK during the COVID-19 pandemic https://www.rcm.org.uk/media/4161/rcm-briefing-on-reintroduction-of-visitors-to-maternity-units-in-the-covid-pandemic-003.pdf	15/07/2020
RCM_3	Virtual consultations https://www.rcm.org.uk/media/4192/virtual-consultations-v20-24-july-2020-review-24-august-2020-1.pdf	24/07/2020
RCM_7	Guidance for intrapartum care for women with COVID-19 https://www.rcm.org.uk/media/4193/intrapartum-care_with-covid-19_20200724-v9-1.pdf	24/07/2020
RCM_10	RCM Clinical Briefing Sheet - Waterbirth during the COVID-19 Pandemic https://www.rcm.org.uk/media/4188/waterbirth-during-covid-19-july-20.pdf	19/07/2020
RCM_31	RCM & RCOG urge NHS England to publish guidance on partners during pregnancy https://www.rcm.org.uk/media-releases/2020/september/rcm-and-rcog-urge-nhs-england-to-publish-guidance-on-partners-during-pregnancy	08/09/2020

For peer review only

RCM_27	Birth partners https://www.rcm.org.uk/media/4339/birth-partners-v2-220920.pdf	20/09/2020
RCM_30	Leading Royal Colleges urge the NHS to learn lessons and avoid redeploying maternity staff ahead of second wave https://www.rcog.org.uk/en/news/leading-royal-colleges-urge-the-nhs-to-learn-lessons-and-avoid-redeploying-maternity-staff-ahead-of-second-wave/	30/09/2020
RCM_28	RCM letter to The Times responding to Times column on COVID rules and pregnancy https://www.rcm.org.uk/media-releases/2020/september/rcm-letter-to-the-times-responding-to-times-column-on-covid-rules-and-pregnancy/	02/10/2020
RCM_38	Partners and visitors<br>https://www.rcm.org.uk/partners-and-visitors/	Oct-20
RCM_39	Reduce infection risks to keep pregnant women and their babies safe, says RCM https://www.rcm.org.uk/media-releases/2020/october/reduce-infection-risks-to-keep-	02/11/2020
RCM_41	Midwives call for common sense on maternity visiting guidance<br>https://www.rcm.org.uk/media-releases/2020/december/midwives-call-for-common-sense-on-maternity-visiting-guidance/	15/12/2020
**Sands**		
Sands_1	Sands feedback: experiences of pregnant women, bereaved parents and families during the COVID-19 pandemic. No longer on website, available on request	19-05-2020 to 27/05/2020
Sands_1	Written evidence from SANDS to parliamentary committee Written evidence submitted by SANDS (DEL0092) https://committees.parliament.uk/writtenevidence/4073/html/	May-20
Sands_4	New NHS guidance on partners of pregnant women in England attending scans https://www.sands.org.uk/about-sands/media-centre/news/2020/09/new-nhs-guidance-partners-pregnant-women-england-attending	09/09/2020
**SoR**		
SoR_11	Obstetric ultrasound examinations during the COVID-19 pandemic.<br>Recording of NHS examinations and the use of private ultrasound clinics: A joint statement from the Society and College of Radiographers (SCoR), the Royal College of Midwives (RCM), the Royal College of Obstetricians and Gynaecologists (RCOG) and the British Medical Ultrasound Society (BMUS). https://archive.sor.org/sites/default/files/document-versions/obstetric_ultrasound_examinations_during_the_covid-19_pandemic_v2_0.pdf	24/04/2020

SoR_9	Ultrasound: Frequently asked questions (FAQs) version 1.7 previously on website – superseded by later versions (SoR & BMUS)	27/04/2020
SoR_10	Ultrasound: Frequently asked questions (FAQs) version 1.8 previously on website – superseded by later versions (SoR & BMUS)	21/05/2020
SoR_12	Obstetric ultrasound examinations during the COVID-19 pandemic.<br>Recording of NHS examinations and the use of private ultrasound clinics: A joint statement from the Society and College of Radiographers (SCoR), the Royal College of Midwives (RCM), the Royal College of Obstetricians and Gynaecologists (RCOG) and the British Medical Ultrasound Society (BMUS). https://archive.sor.org/sites/default/files/document-versions/obstetric_ultrasound_examinations_during_the_covid-19_pandemic_v2_0.pdf	27/05/2020
SoR_6	SoR demands NHS Trusts defend sonographers from populist attacks<br>https://www.sor.org/news/sor-demands-nhs-trusts-defend-sonographers-populist-attacks	04/11/2020
SoR_4	Ultrasound: Frequently asked questions (FAQs) version 1.10 previously on website – superseded by later versions (SoR & BMUS)	04/11/2020
SoR_7	Letter from SoR Resisting populist pressure to minimise the risks to ultrasound staff & patients.<br>Document supplied by SoR	06/11/2020
SoR_8	Webpage: New national guidance on partners attending antenatal scans<br>https://www.sor.org/news/obstetrics-and-gynaecology/new-national-guidance-on-partners-attending-antena	16/12/2020

For peer review only

t

For peer review only

COREQ (COnsolidated criteria for REporting Qualitative research) Checklist

A checklist of items that should be included in reports of qualitative research. You must report the page number in your manuscript where you consider each of the items listed in this checklist. If you have not included this information, either revise your manuscript accordingly before submitting or note N/A.

Topic	Item No.	Guide Questions/Description	Reported on Page No.
**Domain 1: Research team and reflexivity**			
Personal characteristics			
Interviewer/facilitator	1	Which author/s conducted the interview or focus group?	7
Credentials	2	What were the researcher's credentials? E.g. PhD, MD	Title
Occupation	3	What was their occupation at the time of the study?	Title/7
Gender	4	Was the researcher male or female?	7
Experience and training	5	What experience or training did the researcher have?	Title/7
Relationship with participants			
Relationship established	6	Was a relationship established prior to study commencement?	7
Participant knowledge of the interviewer	7	What did the participants know about the researcher? e.g. personal goals, reasons for doing the research	7
Interviewer characteristics	8	What characteristics were reported about the inter viewer/facilitator? e.g. Bias, assumptions, reasons and interests in the research topic	7
**Domain 2: Study design**			
Theoretical framework			
Methodological orientation and Theory	9	What methodological orientation was stated to underpin the study? e.g. grounded theory, discourse analysis, ethnography, phenomenology, content analysis	5
Participant selection			
Sampling	10	How were participants selected? e.g. purposive, convenience, consecutive, snowball	6
Method of approach	11	How were participants approached? e.g. face-to-face, telephone, mail, email	6
Sample size	12	How many participants were in the study?	7
Non-participation	13	How many people refused to participate or dropped out? Reasons?	7
Setting			
Setting of data collection	14	Where was the data collected? e.g. home, clinic, workplace	6
Presence of non-participants	15	Was anyone else present besides the participants and researchers?	N/A
Description of sample	16	What are the important characteristics of the sample? e.g. demographic data, date	7
Data collection			
Interview guide	17	Were questions, prompts, guides provided by the authors? Was it pilot tested?	6/Supp File
Repeat interviews	18	Were repeat inter views carried out? If yes, how many?	N/A
Audio/visual recording	19	Did the research use audio or visual recording to collect the data?	6
Field notes	20	Were field notes made during and/or after the inter view or focus group?	N/A
Duration	21	What was the duration of the inter views or focus group?	6
Data saturation	22	Was data saturation discussed?	6
Transcripts returned	23	Were transcripts returned to participants for comment and/or	6

For peer review only

Topic	Item No.	Guide Questions/Description	Reported on Page No.
		correction?	
**Domain 3: analysis and findings**			
Data analysis			
Number of data coders	24	How many data coders coded the data?	6
Description of the coding tree	25	Did authors provide a description of the coding tree?	11
Derivation of themes	26	Were themes identified in advance or derived from the data?	6
Software	27	What software, if applicable, was used to manage the data?	Excel/6
Participant checking	28	Did participants provide feedback on the findings?	6
Reporting			
Quotations presented	29	Were participant quotations presented to illustrate the themes/findings? Was each quotation identified? e.g. participant number	12-20
Data and findings consistent	30	Was there consistency between the data presented and the findings?	12-20
Clarity of major themes	31	Were major themes clearly presented in the findings?	12-20
Clarity of minor themes	32	Is there a description of diverse cases or discussion of minor themes?	All data includ

Developed from: Tong A, Sainsbury P, Craig J. Consolidated criteria for reporting qualitative research (COREQ): a 32-item checklist for interviews and focus groups. *International Journal for Quality in Health Care*. 2007. Volume 19, Number 6: pp. 349 – 357

Once you have completed this checklist, please save a copy and upload it as part of your submission. DO NOT include this checklist as part of the main manuscript document. It must be uploaded as a separate file.

For peer review only

BMJ Open

Companionship for women using antenatal and intrapartum care in England during COVID-19: A mixed-methods analysis of national and organisational responses and perspectives

Journal:	*BMJ Open*
Manuscript ID	bmjopen-2021-051965.R1
Article Type:	Original research
Date Submitted by the Author:	13-Sep-2021
Complete List of Authors:	Thomson, Gillian; University of Central Lancashire, School of Health<br>Balaam, Marie-Claire; University of Central Lancashire,<br>Nowland (Harris), Rebecca; University of Central Lancashire, School of Nursing<br>Crossland, Nicola; University of Central Lancashire, School of Health<br>Moncrieff, Gill; University of Central Lancashire, School of Community Health & Midwifery<br>Heys, Stephanie; North West Ambulance Service NHS Trust<br>Sarian, Arni; University of Central Lancashire, School of Medicine<br>Cull , Joanne; University of Central Lancashire, School of Community Health & Midwifery<br>Downe, Soo; University of Central Lancashire, research in childbirth and health
**Primary Subject Heading**:	Obstetrics and gynaecology
Secondary Subject Heading:	Qualitative research, Health services research, Public health
Keywords:	GYNAECOLOGY, Organisational development < HEALTH SERVICES ADMINISTRATION & MANAGEMENT, Risk management < HEALTH SERVICES ADMINISTRATION & MANAGEMENT, Public health < INFECTIOUS DISEASES, QUALITATIVE RESEARCH

SCHOLARONE™
Manuscripts

BMJ

I, the Submitting Author has the right to grant and does grant on behalf of all authors of the Work (as defined in the below author licence), an exclusive licence and/or a non-exclusive licence for contributions from authors who are: i) UK Crown employees; ii) where BMJ has agreed a CC-BY licence shall apply, and/or iii) in accordance with the terms applicable for US Federal Government officers or employees acting as part of their official duties; on a worldwide, perpetual, irrevocable, royalty-free basis to BMJ Publishing Group Ltd ("BMJ") its licensees and where the relevant Journal is co-owned by BMJ to the co-owners of the Journal, to publish the Work in this journal and any other BMJ products and to exploit all rights, as set out in our licence.

The Submitting Author accepts and understands that any supply made under these terms is made by BMJ to the Submitting Author unless you are acting as an employee on behalf of your employer or a postgraduate student of an affiliated institution which is paying any applicable article publishing charge ("APC") for Open Access articles. Where the Submitting Author wishes to make the Work available on an Open Access basis (and intends to pay the relevant APC), the terms of reuse of such Open Access shall be governed by a Creative Commons licence – details of these licences and which Creative Commons licence will apply to this Work are set out in our licence referred to above.

Other than as permitted in any relevant BMJ Author's Self Archiving Policies, I confirm this Work has not been accepted for publication elsewhere, is not being considered for publication elsewhere and does not duplicate material already published. I confirm all authors consent to publication of this Work and authorise the granting of this licence.

For peer review only

Title: Companionship for women using antenatal and intrapartum care in England during COVID-19: A mixed-methods analysis of national and organisational responses and perspectives

Authorship

*Gill Thomson, Professor in Perinatal Health, School of Community Health & Midwifery, University of Central Lancashire, Preston, UK. PR1 2HE. GThomson@uclan.ac.uk

Marie-Clare Balaam, School of Community Health & Midwifery, University of Central Lancashire, Preston, UK.

Rebecca Nowland, School of Nursing, University of Central Lancashire, Preston, UK.

Nicola Crossland, School of Community Health and Midwifery, University of Central Lancashire, Preston, UK.

Gill Moncrieff, School of Community Health & Midwifery, University of Central Lancashire, Preston, UK.

Stephanie Heys, Consultant Midwife, North West Ambulance Service, Bolton, UK.

Arni Sarian, School of Medicine, University of Central Lancashire, Preston, UK.

Joanne Cull, School of Community Health & Midwifery, University of Central Lancashire, Preston, UK.

Soo Downe, School of Community Health & Midwifery, University of Central Lancashire, Preston, UK.

And the ASPIRE-COVID19 Collaborative Group

* Corresponding author

Word count: 6,427

ASPIRE-COVID19 Collaborative Group

Co-investigators
Soo Downe; University of Central Lancashire, George Ellison; University of Central Lancashire, Alan Fenton; Newcastle upon Tyne Hospitals NHS Foundation Trust, Alexander Heazell; University of Manchester, Ank de Jonge; Amsterdam University Medical Center, Carol Kingdon; University of Central Lancashire, Zoe Matthews; University of Southampton, Alexandra Severns; NHS England and NHS Improvement North West, Gill Thomson; University of Central Lancashire, Anastasia Topalidou; University of Central Lancashire, Alison Wright; Royal Free Teaching Hospital in London

Research Staff
Naseerah Akooji; University of Central Lancashire, Marie-Clare Balaam; University of Central Lancashire, Jo Cull; University of Central Lancashire, Lauri van den Berg; Amsterdam

For peer review only - http://bmjopen.bmj.com/site/about/guidelines.xhtml

University Medical Center, Nicola Crossland; University of Central Lancashire, Claire Feeley; University of Central Lancashire, Beata Franso; Amsterdam University Medical Center, Steph Heys; University of Central Lancashire, Gill Moncrieff; University of Central Lancashire, Rebecca Nowland; University of Central Lancashire, Arni Sarian; University of Central Lancashire

Steering committee

Maria Booker; Birthrights, Jane Sandall; Kings College London, Jim Thornton (chair); The University of Nottingham, Tisian Lynskey-Wilkie; University of Central Lancashire, Vanessa Wilson; Lancashire and South Cumbria Local Maternity System

Stakeholder Group

Rebecca Abe and Tinuke Awe; FivexMore, Toyin Adeyinka; MVP BAME group, Ruth Bender-Atik; The Miscarriage Association, Lia Brigante; RCM, Rebecca Brione; Birthrights, Franka Cadée; International Confederation of Midwives, Elizabeth Duff; Expert, postnatal care, Tim Draycott; Royal College of Obstetricians and Gynaecologists, Duncan Fisher; Fathers included/Family included/ the Family Initiative, Annie Francis; Independent Midwifery Advisor, Arie Franx; Erasmus MC, Lucy Frith; University of Liverpool, Louise Griew; National Maternity Voices, Clea Harmer; Sands, Caroline Homer; Burnet Institute, Australia, Marian Knight; National Perinatal Epidemiology Unit, Amali Lokugamage; Whittington Health NHS Trust/ University College London, Amanda Mansfield; London Ambulance Service Trust, Neil Marlow; University College London, Trixie Mcaree; NHS England, David Monteith; Grace in Action, Keith Reed; Twins Trust, Yana Richens; UCL & City University, Lucia Rocca-Ihenacho; Midwifery Unit Network, Mary Ross-Davie; Royal College of Midwives Scotland, Seana Talbot; BirthWise NI, Myles Taylor; British Maternal and Fetal Medicine Society, Maureen Treadwell; Birth Trauma Association

For peer review only

For peer review only - http://bmjopen.bmj.com/site/about/guidelines.xhtml

ABSTRACT

Objectives: To explore stakeholders' and national organisational perspectives on companionship for women using antenatal and intrapartum care in England during COVID-19, as part of the ASPIRE COVID-19 study.

Setting: Maternity care provision in England.

Participants: Interviews were held with 26 national governmental, professional, and service-user organisation leads (July-Dec 2020). Other data included public-facing outputs logged from 25 maternity Trusts (Sept/Oct 2020) and data extracted from 78 documents from eight key governmental, professional, and service-user organisations that informed national maternity care guidance and policy (Feb-Dec 2020).

Results: Six themes emerged: '*Postcode lottery of care*' highlights variations in companionship and visiting practices between trusts/locations, '*Confusion and stress around 'rules''* relates to a lack of and variable information concerning companionship/visiting. '*Unintended consequences'* concerns the negative impacts of restricted companionship or visiting on service-users and staff, '*Need for flexibility'* highlights concerns about applying companionship and visiting policies irrespective of need, ''*Acceptable' time for support'* highlights variations in when and if companionship was 'allowed' antenatally and intrapartum; and '*Loss of human rights for gain in infection control'* emphasizes how a predominant focus on infection control was at a cost to psychological safety and women's human rights.

Conclusions: Policies concerning companionship and visiting have been inconsistently applied within English maternity services during the COVID-19 pandemic. In some cases, policies were not justified by the level of risk, and were applied indiscriminately regardless of need. There is an urgent need to determine how to sensitively and flexibly balance risks and benefits and optimise outcomes during the current and future crisis situations.

Strengths and limitations of this study

- This is the first paper to consider links between policy and practice in companionship and visiting in maternity care during the COVID-19 pandemic.
- Data triangulation across national level stakeholders in policy and practice and public facing Trust documentation provides nuanced and context related perspectives on why and how companionship and visiting was impacted.

For peer review only - http://bmjopen.bmj.com/site/about/guidelines.xhtml

- Stakeholders included national representatives from all key agencies involved in maternity care.
- Practice related issues were collected from 25 Trusts websites and social media-based public facing information, which may or may not reflect actual care practices.
- The paper focuses on antenatal and intrapartum care, with postnatal (including neonatal) care to be the focus of future publications.
- The study does not include information directly reported by parents or healthcare professionals.

INTRODUCTION

In many cultures around the world, pregnancy is framed as a social event rather than a clinical condition(1-3). Even where pregnancy, labour and birth are framed as medically risky, social support is expected. Such support, generally framed as companionship, is usually provided through ongoing family and community relationships, often by female relatives and friends, or community members(4). As maternity care has become more hospital based, the power to determine who should accompany women in clinics and facility settings has shifted to the organisation, and its employees(5). In the general hospital setting, the public is usually divided into 'patients' or 'visitors'. During the early decades of mass hospitalisation for antenatal care and birth in the UK, accompaniment for pregnant, childbearing, and postnatal women was either disallowed, or conceptualised as 'visiting', and restricted to specific visiting hours. These limitations were justified on the grounds of infection control, overcrowding, privacy for others, and defence from potential litigation if the accompanying partner or partners witness activities they perceive to be negligent or dangerous(6-8).

More recently the evidence for the value of continuous support and companionship from partners, family, or doulas during intrapartum care has increased(1). Companionship in this context has been associated with four key attributes: informational support, advocacy, practical support, and emotional support(1). Where the companion is a co-parent, there is also a high level of investment in the nature and experience of care provided, whether antenatally, intrapartum, or postnatally. Arguably, this differs from the less active role of 'hospital visitor'. Qualitative studies show that most women and birthing people value such companionship, and it is strongly recommended within global guidelines(9). Though restrictions persist in some health economies around the world, companionship and visiting policies in maternity clinics

For peer review only - http://bmjopen.bmj.com/site/about/guidelines.xhtml

and hospitals in the UK have become increasingly liberal over the last 40 years. A survey published in 2013 found that over half of fathers/co-parents attended at least one antenatal check, and that 'almost all' were present for ultrasound screening in pregnancy, and for labour(10). In 2019 97% of women in England said that their partner or someone else close to them was involved as much as they wanted them to be during labour and birth(11).

The COVID-19 pandemic brought the issue of both visitors and companions in health facilities worldwide into sharp focus(12). In terms of maternity care, there have been anecdotal accounts of wide variations concerning if and whether women have been permitted companionship or visitor rights at various points throughout the maternity episode, both between countries, and across different care providers within countries. Concerns about women being alone for antenatal contacts, for ultrasound scans (especially when there is bad news), during labour and birth have been widespread and global in media reports (for example: (12-14)).

To understand how companionship and visiting in maternity care during COVID-19 was operationalised organisationally in England during antenatal and intrapartum care, this paper presents an analysis of relevant national level policy documents; interviews with key stakeholders; and a review of public facing information produced by 25 purposively selected maternity providers in England.

METHODS

This mixed-methods study is part of a larger mixed-methods, observational, multi-site comparative research project - Achieving Safe and Personalised maternity care In Response to Epidemics (ASPIRE COVID-19 UK).

Data relating to companionship and visiting during the antenatal and intrapartum period in maternity care were extracted from national policy level documents and interviews with national stakeholders and mapped to analysis of public-facing communication channels from 25 Trusts (maternity care organisations). The Trusts were selected using maximum variation sampling, based on macro-level factors impacting upon health inequalities (area level deprivation reported in the English Indices of Deprivation(15)), meso-level considerations relating to the organisation (Care Quality Commission (CQC) rating and maternal and neonatal mortality figures), and micro-level factors including aspects such as parity and access to care identified within national documents.

For peer review only - http://bmjopen.bmj.com/site/about/guidelines.xhtml

Data collection

Documentary review

Guidelines, position papers, and reports relating to maternity care were collected prospectively between February and December 2020 from key governmental, professional, and service-user organisations. The organisations were identified as those who provide guidance, campaign and/or advocate for national practice and policy in relation to maternity care. These documents were sourced via organisation-based websites and from key stakeholders involved in the ASPIRE study. All documents that concerned maternity care provision were reviewed with any/all data in relation to companionship and visiting during antenatal and intrapartum care extracted and logged in Excel files.

Trust-level public facing communication about maternity service provision

Data related to companionship and visiting in pregnancy, labour and birth were extracted from Trust websites and Trust-related Twitter, Facebook, and Instagram feeds, between September and October 2020. We extracted information on the format of information presented, access to companionship or visiting antenatally (including ultrasound), and during labour and birth (including induction of labour). We also extracted information that discussed personalisation, organizational response to specific additional needs, Trust response to national guidance, rationale for decisions about companionship/visiting rules, and any additional information on companionship/visiting in the context of COVID-19.

Interviews

Purposive sampling was used to recruit individuals from relevant national governmental, professional, and service-user organisation leads involved in maternity care. Key individuals were identified by project and advisory teams and via snowballing. All participants were approached by email and provided with an information sheet about the study. A consent form was either completed and returned via email, or the consent process was audio-recorded at the start of the interview and stored separately from the interview recording. Semi-structured interviews were held July-December 2020 via videoconferencing (using Microsoft Teams). Interviews lasted between 45-60 minutes, were audio recorded and transcribed in full. The interview schedule (see Supplementary File 1) explored stakeholders' perceptions and experiences of what, why and how changes in maternity care delivery had been made during the pandemic, how changes had been monitored and assessed, and their views on facilitators

For peer review only - http://bmjopen.bmj.com/site/about/guidelines.xhtml

and barriers to those changes. A total of 50 individuals were approached to participate and interviews were held with 26 stakeholders. While some stakeholders did not respond to the requests, others provided names of individuals who they considered would be more suitable. Recruitment was not based on saturation but rather was designed to ensure that we included representation from all key organisations and from individuals that were considered to offer important insights into maternity care delivery.

Ethics approval

Ethics approval to undertake the interviews was obtained from the Health ethics sub-committee from the lead author's institution (project no: 0079). Participants were asked if they would like to check any public facing quotes prior to publication as part of the consent process, with quotes returned to seven participants for feedback, and four required slight changes to be made. All feedback either involved minor grammatical changes; ensuring the quote was being used in the correct context; or, in one case, a change to job title.

Data analysis

All data analysis was undertaken by hand using Excel. Data analysis for the different forms of data (interviews, documents, Trust level data) were initially undertaken separately (by RN, MCB and NC respectively) before being combined into five key themes using a descriptive content analysis approach(16). The stages of analysis were as follows:

a) Trust-level data were mapped to different aspects of care (e.g. appointments, ultrasound, induction, labour/birth) to identify any variations in maternity service companionship/visiting policies**.**

b) All interview and documentary data that concerned companionship/visiting were extracted and then read on a line-by-line basis to inductively identify meaning units - *'the constellation of words or statements that relate to the same central meaning'*((17)p.106). The meaning units were 'manifest' in terms of identifying the visible and salient components in the data.

c) The meaning unit labels, and associated data were then grouped and synthesised into themes. This process is referred to as *'abstraction'* by emphasising descriptions at a *'higher logical level'*((17)p.106);

d) In the final phase, the Trust level data were integrated into the themes for reporting purposes.

For peer review only - http://bmjopen.bmj.com/site/about/guidelines.xhtml

Four members of the team (GT, RN, MCB, NC) were involved in data analysis, and the final themes were agreed by all named authors.

Public and Patient Involvement

This study was funded under a rapid response call and while no formal PPI involvement in the original design of the study, UK service-user leads (Maternity Voices Partnership) and members of national charities and from service-user organisations are involved as co-investigators, steering group and advisory members for the ASPIRE project, to ensure that service-user inputs have been considered at every stage of the study.

Reflexivity

The authors and members of the collaborating group associated with this study are from a range of academic and clinical backgrounds including midwifery, psychology, obstetrics, neonatology, sociology, and social statistics. All the authors are female, and the four interviewers are experienced in undertaking qualitative interviews. One had previously collaborated with some of the interviewees. All authors believe that women and birthing people highly value companionship during key moments in their maternity care, and that, for many fathers/co-parents, being present is more than just being a visitor or a supporter. From her psychological background, RN also believes that birth supporters other than fathers/coparents play a significant role during childbirth in promoting psychological well-being. As midwives, SD, GM, JC, and SH view companionship for women throughout labour and during antenatal care as a normative practice.

RESULTS

All the twenty-six interview participants held a national and/or strategic role in midwifery (n=9), obstetrics (n=1), neonatology (n=1), anaesthesia (n=1), radiography/sonography (n=2) or as an NHS improvement lead (n=1). One was from a doula organisation, three were from the Maternity Voice Partnership (a NHS working group comprising lay members and professionals dedicated to improving maternity care), five were from national charities (focused on birth trauma, premature/sick infants, stillbirth, miscarriage, multiple births) and two service-user organisations that campaign and advocate for maternity care improvements.

For peer review only - http://bmjopen.bmj.com/site/about/guidelines.xhtml

All the documents were collected from eight governmental, professional, and service-user sources (Box 1), with a total of 78 documents providing evidence for the paper (see Supplementary File 2 for full details/references).

Box 1: Organisations included for documentary analysis

Sands (national charity focused on stillbirth)
AIMS (service-user organisation dedicated to improving maternity care)
Royal College of Midwives (RCM)
Royal College of Obstetricians and Gynaecologists (RCOG)
Society of Radiographers (SoR)
International Society of Ultrasound in Obstetrics and Gynaecology (ISUOG)
NHS England (NHSE)
Birthrights (BR) (service-user organisation dedicated to improving maternity care)

The public facing data logged between September and October 2020 demonstrated a very wide range of policies and practices between the 25 included Trusts for companionship/visiting during four specific maternity care episodes (antenatal scanning; antenatal appointments; antenatal ward stays; and intrapartum) (see Table1). While this could be explained by different COVID-19 infection exposure rates, this may not explain variation between Trusts in the same region.

Table 1: Public facing information on maternity service companionship and visiting policies during antenatal and intrapartum care in 25 English maternity Trusts, September to October 2020

	**Antenatal**			**Intrapartum**		
	**Ultrasound**	**Antenatal appointments**	**Antenatal ward**	**Number of birth partners**	**Timing**	**Induction of labour**
Greater London 1	Unaccompanied	Unaccompanied	Booked time slot	One	Throughout labour	Partner allowed if assessed as needing support
Greater London 2	Partner allowed (20 weeks) *	Unaccompanied	2-6pm	Two	Not specified	No information

For peer review only

Greater London 3	Partner allowed (20 weeks)	No details	No visitors	One (two if additional need identified)	Established labour	No information
Greater London 4	Unaccompanied	Unaccompanied	No visitors	One	Established labour	No information
Greater London 5	Partner allowed	Unaccompanied	Daytime	One (two if need identified)	Not specified	No information
Greater London 6	Partner allowed	No details	Daytime	One	Throughout labour	Partner allowed daytime only
South East 1	Unaccompanied	One companion	Booked time slot	One	Not specified	No information
South East 2	Partner allowed (12 and 20 weeks)	No details	Daytime	Two	Not specified	Partner allowed
South East 3	Unaccompanied	Unaccompanied	Daytime	One	Throughout	No information
South West 1	Partner allowed (12 and 20 weeks)	Unaccompanied	Booked time slot	One	Not specified	Daytime only
South West 2	Partner allowed (20 weeks)	Unaccompanied	No visitors	One	Throughout	Partner not allowed until labour has started
South West 3	Partner allowed (20 weeks)	No details	No information	One	Throughout	Partner allowed daytime or if additional support needed
West Midlands 1	Unaccompanied	Unaccompanied	No visitors	One	Not specified	No information
West Midlands 2	Unaccompanied	Unaccompanied	No information	One	Not specified	Partner allowed if language/communicatio n needs
East Midlands 1	Partner allowed (12 and 20 weeks)	Unaccompanied	No information	One	Throughout	No information
East of England 1	Partners allowed	No information	Booked time slot	One	Throughout	No information
East of England 2	Partner allowed (20 weeks)	Unaccompanied	No information	One	Established labour	Partner not allowed until labour has started
East of England 3	Partner allowed (12 and 20 weeks)¶	Unaccompanied	2-6 pm	One	Not specified	No information
Yorkshire and Humber 1	Partners allowed	No details	No visitors	One	Not specified	Partner not allowed until labour has started
Yorkshire and Humber 2	Partner allowed (12 weeks)	No details	Booked time slot between 1-5pm	One	Throughout	No information
North West 1	Partner allowed (12 and 20 weeks)	Unaccompanied	Booked time slot	One	Throughout	One partner allowed

For peer review only - http://bmjopen.bmj.com/site/about/guidelines.xhtml

North West 2	Partners allowed	Unaccompanied	No information	Two	Not specified	No information
North West 3	Partner allowed (20 weeks)	Unaccompanied	No information	Two	Not specified	No information
North East 1	Partner allowed (12 and 20 weeks)†	Unaccompanied	No information	One	Not specified	No information
North East 2	Partners allowed	No details	No information	One	Not specified	No information

Note: The term 'partner' is used in this table as this, and 'birth partner', were the most commonly used terms to refer to an antenatal or intrapartum companion.

* Phone call offered if clinical concerns

¶ Video offered of a small section at the end of scan

† Phone call offered (end of scan)

Details of themes

Overall, six themes emerged from synthesising the meaning units from the documentary and interview data sets (see Table 2). (Further details of the documents and interviews that generated data for each theme are provided in Supplementary File 3).

Table 2: Theme and associated meaning units from the documentary and interview data

**Themes**	**Meaning units**	
	**Documentary data**	**Interview data**
**Postcode lottery of care**	Different policies used in local situations	Trust dictates rationale for decision making
	Tensions between national and local policy and practise	Differences between trusts resulting in geographical variations
**Confusion and stress around rules**	Concern over transparency, clarity and rationale for decision making	Confusion with rules leads to frustrated parents
		Confusions between staff about the rules
**Unintended consequences**	The need for birth companions as they improve wellbeing and outcomes for women (and the negative impact of not having birth companions)	Lack of companionship created a distressing and frightening experience for women

For peer review only - http://bmjopen.bmj.com/site/about/guidelines.xhtml

	The unintended consequences of lack of/restrictions on birth companions	Lack of support for mother from partner or other family members
	The presence of birth partners supports staff	Increased work burden for staff
	The need to provide alternative support for women if birth partner not present	Being alone when getting bad news at the scan
**Need for flexibility**	Need for consideration of women who are identified to be particularly vulnerable, marginalised or need extra support (e.g. due to ethnicity, language issues, baby loss)	Maternity services should be an exception
	The need to look at situations on a case by case basis to support personalised care	Rules should be applied flexibly to meet the needs of vulnerable women
		Importance of being flexible with rules when babies die
**Acceptable time for support**	The use of virtual means to replace physical companionship in antenatal scans	Ultrasound - rigidity and lack of flexibility
	Concerns over lack of companionship in early labour and for women who are induced and need for support at this time	Knowing when to bring the partner in with active labour - impacts of being in prolonged labour alone
	Concerns over women only allowed support in 'active' labour and how this is determined.	Issues around companionship at the time of induction
**Loss of human rights for gain in infection control**	The balance between risk of transmission and the risks to women.	Expectation that women's rights around childbirth needed to be sacrificed for safety
	The assertion of women's (and partners) human rights as the basis of companionship	Limited focus on safety, centred on infection control

For peer review only

Postcode lottery of care

The notion of a postcode lottery of maternity service provision gained traction in the media over the summer of 2020(18). Concerns were reflected in national documents from almost all included organisations, the stakeholder interviews, and reflected in Trust level responses. Variation was justified by some organisations as a reaction to local need, for example: *'restrictions on other visitors should follow hospital policy and national guidance*' (RCM_7) and '*all staff should work to the same local policy, to provide a consistent service to women'* (SoR_5). The caveat to most guidance was that any policies needed to be re-addressed in the

For peer review only - http://bmjopen.bmj.com/site/about/guidelines.xhtml

event of local spikes in COVID-19 cases or following local risk assessment, to ensure a '*consistent service to women*' (SoR_2):

> But it's guidance, and every hospital will make its own decisions and to a certain extent, they will have to because the physicality, the layout, the facilities that they have in hospitals will differ. You know, there's not much room in the waiting area if the corridors are very narrow [so] that the people can't have a two-metre distance. (Stakeholder 20, National charity)

Some Trusts were identified as having '*gone out of their way to ensure their services remain family-centred*' (BR_14), whereas in others, partners or other companions of choice were unable to attend any antenatal appointments or scans (BR_16). NHS England released guidance in September 2020 intended to assist Trusts to reintroduce access for partners and visitors (NHSE_8. Some organisational responses claimed that this led to '*some Trusts starting to backtrack and reduce restrictions in maternity services*' (BR_17), while other Trusts continued to impose restrictions (BR_18) and often without a clear rationale for these variations:

> And that's the problem. You… because down the street, down the road, you could get a very warm, empathetic ultrasonographer who says, of course, yeah, I realize how difficult is. You know, it doesn't take much, and we just have lost it because people are stressed and there's lots of reasons for it, but it's just not good enough. (Stakeholder 18, Midwifery – Strategic role)

Confusion and stress around 'rules'

Concern over a lack of clarity in decision making and changes in policies around companionship/visiting were highlighted. For instance, letters from Birthrights to maternity leads (e.g. BR_12, BR_16, BR_19) repeatedly emphasised the need for clear reasons, evidence, and justification as to why decisions were being made. Concerns included that Trusts '*acted too quickly to withdraw services*' and '*decision-making has not always been proportionate or transparent*' (BR_18). While most Trusts made some reference to infection risk as the rationale for the restrictions, a sizeable minority (9/25) did not. Many Trusts offered no rationale as to why partners could attend some appointments but not others, e.g.:

For peer review only - http://bmjopen.bmj.com/site/about/guidelines.xhtml

> One birth partner may attend for the 20-week anomaly scan only. Women to attend all other scan appointments unaccompanied (South West 2).

Birthrights and some stakeholders highlighted a failure to communicate local restrictions in a timely manner, compounded by rules changing rapidly and difficulties in communicating these changes widely and consistently to large numbers of healthcare professionals. This confusion about the rules at local level was also compound by changeovers of staff and communication between different teams in some areas – *'there's been no consultation with sonographers in terms of risk assessments* […] *or changes in practice'* (Stakeholder 26, Radiographer), and services being provided by staff from outside the maternity team.

While some stakeholders noted that individual Trusts responded to this confusion by using a range of public communications, data from the 25 maternity Trusts found that less than half (10/25) had a consistent message about companionship or visiting across different channels. Parent frustration reported by stakeholders also related to how the rules for birth companions and visiting seemed at odds with the social distancing rules outside of the hospital context:

> In the middle of lockdown, it was 'we don't like it, but we know you're keeping us safe'. Now 'it's we don't like it and I don't see how you're keeping us more safe doing this because I can meet my partner in the pub, but he can't come to my scan. I can do this, but I can't do that'. Yes. So, it's more of an angry mood now. (Stakeholder 12, Maternity Voices Partnership)

In some of the documents by Birthrights (e.g. BR_14; BR_16) and RCM (i.e. RCM_31) they called for the harm caused by restricted access by birth partners to be properly and transparently considered within the decision-making processes:

> We would be grateful if you could publish or send us the risk assessment that quantifies the increased risk of spreading COVID within the unit (despite PPE and other mitigating factors, and the fact that most partners are from the same household) caused by relaxing restrictions, and weighs this against the known harms to pregnant women, birthing people and their families from keeping the current restrictions in place. (BR_16)

For peer review only - http://bmjopen.bmj.com/site/about/guidelines.xhtml

Unintended Consequences

Organisations including RCM, RCOG, NHS England and Birthrights highlighted that having trusted companions throughout labour and birth is linked to improved outcomes, and a lack of companionship was associated with increased need for pharmacological or other interventions (AIMS_8, BR_23). This included perceived impacts on the labour process due to, for example, [increased] *'demand for epidurals'* (RCM_8). Alongside the obvious fear and anxiety of infection, organisations and stakeholders highlighted concerns about women feeling '*petrified'* (Stakeholder 15, Midwifery – Strategic role) or not accessing maternity care, '*due to the women's preferred birth partner not being allowed to accompany her*' (RCM_2). There were also concerns of partners feeling *'unsupported and uncared for'* (Stakeholder 20, National charity) due to being unable to be with their partner when they heard bad news (during ultrasound) or missing the birth due to *'being told that they should wait in the car park or something'* (Stakeholder 7, Service-user organisation). All but four Trust websites contained messages of empathy regarding the restrictions, sometimes alongside expressions of regret and/or justifications for their necessity: '*We understand the restrictions we have had in place over recent months have been particularly hard for pregnant women and their families*' (North West 1). Concerns were also expressed by stakeholders and within documents by Sands and Birthrights, towards women who had experienced prior baby loss or for women for may receive bad news alone during the scan:

> Women are being asked to attend scans alone, with many sharing frustrations that they cannot video link to their partners. These very vulnerable women tell us they are concerned about having to attend stressful antenatal appointments and scans alone. While units are being encouraged to consider facilitating women to take a video clip at the end of an appointment, this is reliant on local policies. (Sands_1)

Companionship was noted to have practical as well as emotional implications for women. Some of the documents claimed that the absence of companions meant that women required more support from maternity care professionals (e.g. RCOG/RCM_1j; NHSE_1) creating additional stress for over stretched services (RCOG/RCM_1l), and additional potential exposure to COVID infection. Some practitioner respondents also reported that they or their colleagues experienced moral distress when social distancing rules prohibited physical contact with women who were alone, or receiving bad news:

For peer review only - http://bmjopen.bmj.com/site/about/guidelines.xhtml

> All of a sudden it was just women on their own for us. And that was really, really stressful for the women and the staff. And a lot of a lot of my job is giving bad news. And to give that to women that are on their own with no support; you can't touch them. You can't hug them. And so that for us is really, really challenging. I think that was probably the most challenging thing. (Stakeholder 24, Sonographer)

Need for flexibility

Concerns discussed within the documents (e.g. BR_8; BR_18; RCM_4) and raised by stakeholders related to the blanket adoption of visitation rules across whole hospitals. Some commented that pregnant women and childbearing people were a *'separate population with separate needs'* (Stakeholder 7, Service-user organisation), arguing that visiting rules adopted in other areas of healthcare should not apply to a perinatal population:

> So, you know, several heads of midwifery were saying to me, I want to do this, but they won't let me because they made a decision about what the visiting will look like in this hospital. And they do not see maternity as an exception. And, you know, it is an interesting reflection, isn't it, that maternity has always been a service that has seen itself as an exception to the health care service in which it sits. (Stakeholder 17, NHS improvement lead)

Responses from organisations including AIMS, Birthrights, and the RCM argued how there needed to be consideration of women's unique situations. Restrictions on companionship were considered to have a disproportionate impact on those who were facing disadvantages including those for whom English is a second language, women with mental health problems, cognitive impairments, refugee and asylum seeking women (AIMS_8, RCM_4, BR_8, BR_18). Only one Trust included a statement on their website about offering personalised (flexible/individualised) care for all women, that might not be in line with COVID policies. Five others said they offered this on a case-by-case basis (often expressed as 'exceptional circumstances'). One of these referred to Black, Asian, and Minority Ethnic communities along with concerns about greater COVID risks, and three referred to *'allowing'* women to bring a companion if they *'needed assistance'*. In two Trusts this was explicitly linked to women with disabilities:

For peer review only - http://bmjopen.bmj.com/site/about/guidelines.xhtml

> Partners and family members will not be allowed to enter the building unless you need support from a carer/relative (for example, if you have a disability and need support) (North West 2).

Birthrights stipulated how 'protected characteristics' under the Equality Act 2010 (e.g. physical disability or mental health condition) meant that maternity Trusts were obliged to make reasonable adjustments (e.g. BR_15; BR_18). NHS England emphasised the need for *'essential visitors'* (seen as different to 'normal visitors') for those with specific communication or care needs (NHSE_8). AIMS also stressed that on some occasions, on a '*case by case basis*', such as a disability, *'a second birth partner may be critical to women's mental wellbeing or other needs'* (AIMS_2).

The lack of flexibility for highly sensitive events such as for those who experienced trauma or loss were also highlighted by Birthrights (e.g. BR_18) and RCM (e.g. RCM_41). While some incidents of positive practice were identified, stakeholders also shared stories of parents whose baby had died in utero being unable to take photographs or spend time with their deceased child:

> We had a lot of stories from parents who hadn't been allowed to take photographs, haven't had time to sit and hold their baby. And I think all of those were linked to both a lack of space, to lack of bereavement space, but also a lack of staff understanding of how to adapt bereavement care standards. We also saw in this group a lot of problems around not having the partner with them. (Stakeholder 10, National charity)

'Acceptable' time for support

Access to and timing of support was an issue at key stages during the perinatal journey, and notably during antenatal ultrasound appointments and during early onset of labour. A key area of contention related to women having to attend ultrasound scans unaccompanied: a situation described by one of the stakeholders as *'ludicrous'* (Stakeholder 7, Service-user organisation). RCOG guidance recommended that *'patients should be asked to attend alone if possible or with a maximum of one partner/visitor'* (RCOG_5), whereas a RCM document stated '*partners should attend scans unless rooms are too small to socially distance: partners may attend scans virtually'* (RCM_28). However, in contrast to this permission for virtual contact, a joint statement by SoR, RCOG, RCM and the British Medical Ultrasound Society stated that devices

required for remote contact by partners via video/phones are a vehicle for transmission (due to surface contamination), and that recordings would impact on scan time, sonographer concentration and potential detection of fetal abnormalities – although it was acceptable for the mother (if in line with local policies) to "*save a short 10–30 second cine clip of the fetus at the end of selected examinations"* (SoR_11).

Trust data revealed that while most permitted partners at one or both standard ultrasound appointments (12 and 20 weeks), seven (~30%) did not. Four Trusts mentioned video or other means of 'virtual' companionship, but usually to specify that videos of scans were not permitted. Only one Trust referred to women being able to phone a partner for support if the sonographer were to find "*important clinical information that your partner needs to be aware of*" (Greater London 2). While many stakeholders were critical of the ultrasound restrictions on the right to be accompanied, one respondent argued the need to highlight that antenatal ultrasound scanning continued, even when *'other screening programmes went into hibernation'* (Stakeholder 26, Radiographer). Some defended restrictions on women being accompanied during scanning, noting that scans often have to take place in areas *'like a broom cupboard in a very small poorly ventilated space'* (Stakeholder 17, NHS improvement lead) coupled with the restricted time to undertake the examination and sterilising the room and equipment after each appointment. One reported that there had been a *'downgrading'* of the importance of scans as a medical examination that required focused concentration in challenging situations, during vociferous debate about partner attendance (Stakeholder 26, Radiographer). However, while sonographers may have faced increased risks due to screening large numbers of women, the specific rationale for not allowing videos as an alternative was challenged:

> You can argue the toss as to whether some of the justifications for not allowing that were real or weren't real. You know, is there really a risk of infection if you pick up your phone? Really? Maybe some anxiety for sonographers or whoever's doing the scan. You know, you don't really want the phone on with a video while you're doing the scan because who knows, they might use it in some kind of litigation. Who knows? But whatever it was, it really didn't help. (Stakeholder 20, National charity)

A further area of contention concerned companionship during labour and birth. While organisations such as Birthrights argued for companionship throughout, less than half (9/25)

For peer review only - http://bmjopen.bmj.com/site/about/guidelines.xhtml

the Trusts referred to partners or other birth companions of choice attending '*throughout*' or '*for the duration*'. Three Trusts referred to companionship being permissible only when the woman was in '*established*' or '*active*' labour (with no details as to how this would be established); and 13 Trusts did not specify the relevant phase of labour. RCM guidance advised that women would not be able to have partners present during inductions that took place in a bay or ward (RCM_27). Only six Trusts (25%) indicated that birth partners could be present during induction of labour, and four allowed birth partners, but with limitations (either restricted to daytime or if the woman needed additional support). About half (12) provided no information, and three explicitly stated that birth partners were not allowed:

> If you are attending for induction of labour please attend alone, your birth partner will join you once you are transferred to the Delivery Suite. (Yorkshire and Humber 1)

Birthrights and AIMS (i.e. BR_8; AIMS_5; BR_18; BR_23) also raised concerns about cervical dilatation as the only acceptable indicator of active labour, meaning that some women who may not have wanted (or needed) a vaginal examination felt pressured to accept the procedure if they wanted their chosen birth companion to be granted access to the labour ward.

There were examples of innovation to try to support companionship for women and access for their partners. Some Trusts initiated or extended the provision of labour induction in community settings or in private hospital rooms (rather than multi-occupancy early labour wards) to prevent separation of women from birth partners (RCM_8; RCM_27). Some stakeholders also referred to more flexible approaches to induction such as partners being able to *'come and settle them* [women] *in'* and to use *'Facetime to be with their partner all the time'* (Stakeholder 21, Midwife - National role).

Loss of human rights for gain in infection control

There was some evidence from stakeholders that hospital decision makers in some settings believed that companionship during the maternity episode should not be prioritised over visiting rights in other areas where attendance of close family members would usually be seen as a critical human need and right: especially when someone was dying in hospital:

> So, it was interesting and when I would speak to the head of midwifery, sometimes it felt like they were saying, you know, well, everyone's got to make…. sacrifices. And

For peer review only - http://bmjopen.bmj.com/site/about/guidelines.xhtml

> there are people dying alone in hospital. There are people suffering terribly alone in hospital, unable to have visitors…. [while] there were women saying, you know, it's my right to have a companion it's your job to provide care for me. So, it felt at times like each group with their own concerns was unable to think about or found it difficult to take on board the concerns of the other group. (Stakeholder 17, NHS improvement lead)

The underlying principle within most of the guidance reviewed was that 'safety', was primarily conceptualised as the prevention of transmission of infection, both to service-users and to staff. NHS England documents referred to minimising '*control risks working with your IPC* [Infection, Prevention & Control] *leads, whilst still allowing the maximum possible safe access'* (i.e. NHSE_9). The RCOG/RCM also noted the need to minimise the number of attendees (as ISUOG_5 quote above), but acknowledged that one person could be there for antenatal visits should a woman choose this:

> You will be asked to come alone to clinical appointments or keep the number of people with you to one (including midwifery visits in your home). This will include being asked not to bring your children with you to appointments. This is important to protect maternity staff, other women, and babies, and you and your family from the risk of infection. (RCOG/RCM_1g)

Birthrights was one of the key organisations to recommend that notions of safety might also include emotional and psychosocial risks of women being unattended (e.g. BR_17, BR_16, BR_19, BR_18) - '*The damage caused by ongoing restrictions needs to be weighed up against the requirements of infection control'* (BR_19). Several of their documents (e.g. BR_18; BR_19) claimed that routinely restricting birth companions was a violation of women's (and fathers/co-parents) human rights. Despite this, stakeholders stated that, in practice, women's human rights and choices around companionship did not feature as part of the decision-making processes:

> And I think we've spent the last, you know, however many years banging on about the fact we want to give women choice and rights and sharing that discourse and encouraging women to become empowered. And then COVID comes along and we just say, no, no, we're not doing that (Stakeholder 17, NHS improvement lead)

For peer review only - http://bmjopen.bmj.com/site/about/guidelines.xhtml

The RCM (RCM_41) stated that their '*greatest concern'* was '*safety being sacrificed in favour of popularity'* which seemed to imply that companionship or visiting should not outweigh the need to prevent infection of its *'members'* and of *'women and families'*. SoR also highlighted that its guidance had *'risk assessments'* at its core (SoR6). The guidance did not preclude *'people being accompanied'*, but that it '*must only happen if the safety of the patient and sonographer is not compromised*' (SoR_8). However, others argued that day to day decision making was based around a belief about safety that was limited: '*because it's not just about the physical self, it's about psychological self*' (Stakeholder 14, Midwife – National role):

> But I think safety generally is an interesting thing because….You know, so many different things affect safety don't they, so something like being able to have your partner with you might not be seen as a primary thing affecting safety in comparison with protecting against COVID, but actually if it impacts on someone's mental health in either the partner or the mother, that does have an effect on safety (Stakeholder 5, Maternity Voices Partnership)

DISCUSSION

In this paper we have drawn on guidance from national statutory and service-user organisations, key stakeholders, and public facing Trust-level data to consider the organisational issues associated with companionship and visiting in antenatal and intrapartum care during the COVID-19 pandemic. The terms companionship and visiting were not always clearly differentiated in data relating to the antenatal and intrapartum period, though most sources were consistent in referring to accompaniment as 'companionship' during labour and birth. The value of active companionship during labour and birth for women and birthing people is widely recognised, in terms of clinical benefits, and short and longer term psychosocial impacts(1, 19). As evidenced within this paper, during the COVID-19 pandemic, at the policy and organisational level, assumptions and norms about companionship, accompaniment, and visiting during facility-based health care provision have faced profound challenge. Some of the key organisational challenges have concerned personnel shortage issues, infection control and restricted space. Others have noted the variance in maternity organisation response during the pandemic(20). Some variation can probably be explained by changing national knowledge about the prevalence and impacts of COVID-19, and by different levels of exposure to COVID-19 infection. However, our data suggest that this was not the case

For peer review only - http://bmjopen.bmj.com/site/about/guidelines.xhtml

where blanket policies were applied with minimal individual flexibility, or where there was unjustified variation in visiting and companionship rules, coupled with poor and inconsistent communication. There were no clear patterns in the Trust-level data that would allow us to explain the differences we documented in responses. While population level disparities may be a contributing factor, most Trusts serve a range of sociodemographic/economic areas, and other potentially relevant information such as space constraints was not publicly available.

We found particular concern about lack of access to companionship (in the sense of informational, practical and social support and advocacy(1) in two distinct areas. First, women being unable to have any communication (actual or virtual) with partners or other family members at ultrasound scan; and, second, denial of intrapartum companionship until labour was 'established'. In relation to the former case, there is some evidence that, beyond the emotional and psychological benefits for the mother, when fathers and partners are present for antenatal ultrasound scan, there are significant effects on their identification with the fetus (as their future child) and their empathic relating with the mother(21, 22). This implies that being present for ultrasound scans is more than simply 'visiting'. It has important public health and relationship benefits for the woman/birthing person, and their partner, and baby. In the latter case, in some Trusts, ensuring that labour had progressed sufficiently was perceived by some stakeholders to be associated with coercive and invasive practices, such as regular vaginal examinations when women may otherwise not have needed or wanted such examinations. General uncertainty over organisational companionship permissions during labour and birth may also be reflected in anecdotal rises in women/birthing people choosing to freebirth(23, 24), and the issuing of associated RCM guidance to ensure appropriate professional responses(25). Trust policies that restricted intrapartum companionship until labour was established (or until birth was imminent) seemed to be built on an assumption that companionship was only really needed when labour was very intense, and/or when the birth was happening, so that the birth companion (assumed to be the co-parent in this case) could be 'permitted' to witness to the birth of their baby. In contrast, other Trusts seemed to recognise, at the organisational level, that active and engaged birth companionship throughout labour (from the early stages of spontaneous labour, or from the time of labour induction through to the birth) is a mechanism for clinical, psychological and emotional safety for the mother, partner and child, both in the short term, and, critically, in the longer term, when the threat of COVID-19 infection is long over (12, 26).

For peer review only - http://bmjopen.bmj.com/site/about/guidelines.xhtml

The pandemic brings into sharp focus the fundamental and underpinning ethical dilemma between social actions that ensure the greatest benefit for the population as a whole, and the individual human rights of each person within that population(27). Resolving this potential conflict of ethical imperatives depends on an open and informed debate about rights and consequences. In terms of maternity care, this requires a sophisticated understanding of what 'companionship' (as opposed to 'visiting') means, over the whole life course, and for mother/birthing person, baby, and family. It also requires attention to the potential moral distress of maternity care staff (and health care staff in general, including ultrasonographers). These professionals are faced with the stress of having to balance these two imperatives with real people, in intensely emotional real time, repeatedly day in and day out, and at times with insufficient PPE equipment available; at a time when they, too could be pregnant women at risk of exposure to infection, or parents/carers fearful of infecting family members(28-31).

This is the first study to bring together national policy and organisational stakeholder views with Trust based public facing data to understand how companionship and visiting in antenatal and intrapartum care has been organised in England during COVID-19. Although we cannot be sure we captured every single relevant document produced over the period of our data collection, triangulation across data sources and across multiple researchers enabled rich insights into how and why variations occurred, and the perceived impacts. Returning quotes to stakeholders (as they requested), also provided a further level of rigour. The pragmatic restriction of the Trust level data collection to only 25 Trusts (10% of maternity care providers in the UK), and the restriction to maternity-specific documents and guidance may be a limitation. However, the organisations that were included were selected purposively to reflect a wide range of relevant characteristics. Trust-level data were collected during a discrete period (September and October 2020), aiming to capture responses to changed national guidance; this limitation means we do not address how Trusts continued to respond to the changing pandemic. Since this paper is focused on policy and organisational responses to the pandemic, service-users, birth companions and healthcare professionals at Trust level were not included. In addition, our analysis did not include findings related to postnatal care, or care in neonatal units. These areas, and the unintended (positive and negative) short- and longer-term consequences of different interpretations of the value of companionship (in itself, and as opposed to being a hospital visitor), when balanced against infection control, are critical areas for examination during the on-going COVID-19 crisis. Future outputs from the ASPIRE project will address these gaps.

For peer review only - http://bmjopen.bmj.com/site/about/guidelines.xhtml

CONCLUSION

This paper presents insights from the ASPIRE COVID-19 UK study to understand how companionship and visiting in maternity care was operationalised at the organisational level in antenatal and intrapartum care during COVID-19. Our findings illustrate variations in policy at national and local level, coupled with poor and inconsistent communication of how the restrictions changed in some sites, and a lack of clarity in the decision-making processes. The evidence highlighted a lack of flexibility in responding to women with more complex needs in some cases, the negative and positive unintended consequences of companionship restrictions, and the challenges of conceptualising and balancing infection risk and emotional and psychological distress. However, there was evidence that creative solutions were possible, since, despite significant pressures, some Trusts appeared to continue to provide full companionship.

Overall, these concerns illustrate something much more fundamental than merely barriers to hospital 'visiting'. While the NHS England Better Births policy agenda highlights the need for safety and personalisation within maternity care, these findings suggest that, over the time period captured by this study, personalisation (and emotional and psychological safety) became sacrificed in some (but not all) situations to the overriding imperative to minimise infection spread with high emotional and psychological costs. Further research should capture the views and experiences of healthcare professionals and service-users, and clinical outcome data from different settings. There is an urgent need to determine how to balance risks and benefits sensitively and flexibly and to create optimum outcomes for women, pregnant people, fathers, parents, infants, families, and staff, during the current and future crisis situations.

ACKNOWLEDGEMENTS

A funding statement: This research is funded by the Economic and Social Research Council (ESRC), as part of UK Research and Innovation's rapid response to COVID-19 [grant number ES/V004581/1]. Full details of the main study are available via ResearchRegistry (researchregistry5911) and via UKRI Gateway (https://gtr.ukri.org/projects?ref=ES%2FV004581%2F1)

Author contributions: SD with input from the ASPIRE-COVID19 Collaborative Group designed the study. RN, GT, SD, and NC interviewed the stakeholders; GT, MCB, GM and JC were involved in data extraction for the documentary analysis; SH, AS and NC collected and

For peer review only - http://bmjopen.bmj.com/site/about/guidelines.xhtml

analysed Trust level data. GT, MC, and RN were involved in developing themes from the interview and documentary data, and GT synthesised the Trust level data into the data set. Final themes were agreed with all authors. All authors and the ASPIRE-COVID19 Collaborative Group contributed to writing and reviewing the manuscript.

A data sharing statement: Trust level data is included in the paper. Details of all documents analysed is provided/with all information freely available. All relevant interview data concerning companionship will be openly available from UCLanData and a doi provided on paper acceptance.

Competing interest statement: None of the authors have any competing interests.

REFERENCES

1. Bohren MA, Berger BO, Munthe-Kaas H, Tunçalp Ö. Perceptions and experiences of labour companionship: a qualitative evidence synthesis. *Cochrane Database Syst Rev* 2019;3, doi: 10.1002/14651858.CD012449.pub2

2. Jordan B. Birth in four cultures: A crosscultural investigation of childbirth in Yucatan, Holland, Sweden, and the United States: Waveland Press; 1992.

3. Tani F, Castagna V. Maternal social support, quality of birth experience, and post-partum depression in primiparous women. *J Matern Fetal Neonatal Med* 2017;30(6):689-92.

4. Withers M, Kharazmi N, Lim E. Traditional beliefs and practices in pregnancy, childbirth and postpartum: A review of the evidence from Asian countries. Midwifery. 2018;56:158-70.

5. Draper J. Whose welfare in the labour room? A discussion of the increasing trend of fathers' birth attendance. Midwifery. 1997;13(3):132-8.

6. Jackson SH, Schlichting FR, Hulme RL. Effect of fathers at cesarean birth on postpartum infection rates. AORN 1982;36(6):973-7.

7. Stephan G, Silbermann B. Father in delivery room wins decision that could land you in court. Legal aspects of medical practice. 1979;7(7):49-50.

For peer review only - http://bmjopen.bmj.com/site/about/guidelines.xhtml

8. Kabakian-Khasholian T, Portela A. Companion of choice at birth: factors affecting implementation. BMC Preg Childbirth. 2017;17(1):1-13.

9. World Health Organization. WHO recommendations: intrapartum care for a positive childbirth experience: transforming care of women and babies for improved health and well-being. World Health Organization; 2018.

10. Redshaw M, Henderson J. Fathers' engagement in pregnancy and childbirth: evidence from a national survey. BMC Preg childbirth. 2013;13(1):1-15.

11. Care Quality Commission. 2019 survey of women's experiences of maternity care https://www.cqc.org.uk/sites/default/files/20200128_mat19_statisticalrelease.pdf (Accessed 20 August 2021)

12. Coulter A, Richards T. Care during covid-19 must be humane and person centred.*BMJ* 2020;370:m348

13. Ferber A. How Covid-19 led to a global crisis in maternity care. New Statesman. 17 December 2020 How Covid-19 led to a global crisis in maternity care (newstatesman.com)

14. Black BL, J; McKay, G. Birth partners are not a luxury BMJ 24 September 2020 https://blogs.bmj.com/bmj/2020/09/24/birth-partners-are-not-a-luxury/

15. National Statistics. English indices of deprivation 2019. English indices of deprivation 2019 - GOV.UK (www.gov.uk) (Accessed 15 August 2021)

16. Vaismoradi M, Turunen H, Bondas T. Content analysis and thematic analysis: Implications for conducting a qualitative descriptive study. *Nurs Health Sci* 2013;15(3):398-405.

17. Graneheim UH, Lundman B. Qualitative content analysis in nursing research: concepts, procedures and measures to achieve trustworthiness. *Nurse Educ* 2004;24(2):105-12.

For peer review only - http://bmjopen.bmj.com/site/about/guidelines.xhtml

18. Iacobucci G. Partners' access to scans and birth is a postcode lottery, data show. *BMJ* 2020;371:m3876

19. WOrld Health Organization. Companion of choice during labour and childbirth for improved quality of care: evidence-to-action brief. World Health Organization; 2016.

20. Jardine J, Relph S, Magee LA, von Dadelszen P, Morris E, Ross-Davie M, et al. Maternity services in the UK during the coronavirus disease 2019 pandemic: a national survey of modifications to standard care. BJOG. 2021;28(5):880-889.
doi: 10.1111/1471-0528.16547.

21. Walsh TB, Davis RN, Palladino CL, Romero VC, Vijay Singh M. Moving up the" magic moment": Fathers' experience of prenatal ultrasound. *Fathering* 2014;12(1):18.

22. Draper J. 'It's the first scientific evidence': men's experience of pregnancy confirmation. *J Adv Nurs* 2002;39(6):563-70.

23. Dahlen H. During COVID-19, women are opting for 'freebirthing' if homebirths aren't available. And that's a worry: The Conversation 2020 9 July 2020 https://theconversation.com/during-covid-19-women-are-opting-for-freebirthing-if-homebirths-arent-available-and-thats-a-worry-142261.

24. Greenfield M, Payne-Gifford S, McKenzie G. Between a rock and a hard place: Considering "freebirth" during Covid-19. *Frontiers in Global Women's Health*. 2021;2:5 https://doi.org/10.3389/fgwh.2021.603744.

25. Royal College of Midwives. Clinical Briefing Sheet: 'freebirth' or 'unassisted childbirth' 30 April 2020 https://www.rcm.org.uk/media/3904/freebirth_draft_23-april-v5-002-mrd-1.pdf.

26. Lavender T, Downe S, Renfrew M, et al. Rapid analytic review: Labour and birth companionship in a pandemic: Companionship of choice for asymptomatic childbearing women in hospital throughout labour and birth. *Royal College of*

For peer review only - http://bmjopen.bmj.com/site/about/guidelines.xhtml

Midwives https://www.rcm.org.uk/media/3951/birth-companionship-in-a-pandemic-master-27-04-2020-002.pdf (Accessed 23 February 2021)

27. Xafis V, Schaefer GO, Labude MK, Zhu Y, Hsu LY. The perfect moral storm: Diverse ethical considerations in the COVID-19 pandemic. *Asian Bioeth Rev* 2020;12:65-83.

28. Hunter B, Renfrew MJ, Downe S, et al. Supporting the emotional wellbeing of midwives in a pandemic: Guidance for RCM. 2020. Royal College of Midwives. https://www.rcm.org.uk/media/4095/rcm-supporting-the-emotional-wellbeing-of-midwives-during-a-pandemic-v1-submitted-to-rcm_mrd.pdfm (Accessed 21 February 2021)

29. Jeffrey DI. Relational ethical approaches to the COVID-19 pandemic. *J Medical Ethics* 2020;46(8):495-8.

30. Sheather J, Fidler H. Covid-19 has amplified moral distress in medicine. *BMJ* 2021;372:n28

31. Chew C, Ko D. Medical ethics in the era of COVID-19: Now and the future. *Respirology* https://doi.org/10.1111/resp.13927 (Accessed 5 January 2021)

For peer review only - http://bmjopen.bmj.com/site/about/guidelines.xhtml

Supplementary File 1

Interview schedule

Opening question

In your view, what are the most important issues for maternity care provision that have come out of COVID-19?
Probe: *Are these views based on any particularly memorable experiences?*

Changes/adaptions to service delivery

How have [i.e. maternity, midwifery, obstetric, neonatal, ambulance delivery, staffing, any other?] services been adapted during COVID-19 pandemic?
Prompts: *How significant or minor have these changes been?*
Can you describe some of the primary Covid-19 interventions?
Have these adaptions varied by place and/or time? If so, what do you think explains this variation?

Decision making processes

From your perspective, who makes decisions about the changes?

Prompts: *How are these decisions made?*
Were decisions made at a multidisciplinary level or separately?
How did the different agencies – work together - who had overall responsibility?
Who has been consulted about the changes – are all those impacted consulted (e.g. at UK level were MVPs consulted and/or for neonatal were the local PAG group consulted?)
If there is resistance, how is that dealt with?

How have safety principles (for service-users and staff) informed decision-making/practice changes?

How have personalised care principles informed decision-making/practice changes?
Prompts: *What personalised care aspects have been considered?*

To what extent has the needs and care of BAME/vulnerable populations been considered in the decision-making processes?

What other factors have influenced decision-making/changes in care provision?

Communication and implementation

How have the recommended practices and guidance been developed?
Prompts: *How effective have these methods been?*
How was conflict in what to include resolved?

For peer review only

How have they been communicated to practitioners/front line staff?
Prompts: *How effective have these methods been?*
What more is needed?

How is service delivery being monitored over this period?
Prompts: *What feedback loops are in place*?
How is this information used?

Impact

What has been the (positive and negative) impact of the changes/adaptions on:
- Staff
- Parents and families (siblings/grandparents)
- Service delivery
- Data collection and sharing (i.e. Maternity Services Dashboard and Better Births Minimum Data set)

In which data do you think the effects of the pandemic and the adaptations made to service provision can be seen? Can we have access to the data?

Barriers and facilitators

What has worked well in providing safe and personalised maternity care during the pandemic?
Prompts: *Do any particular Trusts/services/lobby groups and etc stand out for you?*
Why is this?

What have been the challenges or barriers in providing safe maternity care?

What have been the challenges or barriers in providing personalised maternity care?

Recommendations and Sustainability

What else is needed to ensure safe and personalised maternity standards and principles are upheld in:
- future routine maternity care
- a future crisis/pandemic

If there was one thing that you would change about how maternity care was managed what that would be?

What innovations been implemented that could be opportunities for how maternity care could be organized in the future?
Prompts: *Can you identify where these innovations and opportunities are happening at the moment?*

How do you think services should prepare for future occurrences/pandemics?

Is there a specific Trust/Health Board you think we should include in our case studies?

For peer review only

Prompts: *If so, can you tell us why you think this is a particularly relevant site?*

Are there other things, that have not been mentioned that you would like to share?

Is there anyone else that you think we should talk to?

For peer review only

Organisation/Code	Name of Document/Link	Date of documen
**AIMS**		
AIMS_1	Coronavirus and the Maternity Services on AIMS website https://www.aims.org.uk/campaigning/item/coronaviru	25/03/2020
AIMS _2	AIMS Response to the Clinical guide for the temporary reorganisation of intrapartum maternity care during the coronavirus pandemic, version 1, published 9/4/20 https://www.aims.org.uk/assets/media/444/aimscomments2020-04-11.pdf	09/04/2020
AIMS_5	Submission from AIMS to the Health and Social Care Committee Inquiry: Delivering Core NHS and Care Services during the Pandemic and Beyond<br>https://www.aims.org.uk/assets/media/447/aimssubmissionhealthandsocialcarecommittee2020-05.pdf	07/05/2020
AIMS_7	AIMS comments on latest guidance on birth partner restrictions https://www.aims.org.uk/campaigning/item/latest-guidance-birth-partner	25/09/2020
AIMS_6	Press Release: Almost Half of NHS Trusts Force Pregnant Women to Attend Vital Maternity Services Alone https://www.aims.org.uk/campaigning/item/trusts-force-pregnant-women-to-attend-alone	12/10/2020
AIMS_9	AIMS calls for clearer guidance from NHS England on lifting partner restrictions in maternity<br>https://www.aims.org.uk/campaigning/item/nhse-letter	21/11/2020
AIMS_8	AIMS asks Society and College of Radiographers to review guidance on partners and supporters at scans https://www.aims.org.uk/campaigning/item/scor-letter	12/11/2020
**Birthrights**		
BR_1	Human rights charity calls for protection of UK women in childbirth during national emergency https://www.birthrights.org.uk/wp-content/uploads/2020/03/Final-Covid-19-Birthrights-31.3.20.pdf	31/03/2020
BR_8	Coronavirus and the impact on people with protected characteristics https://www.birthrights.org.uk/wp-content/uploads/2020/05/Birthrights-Covid-19-Maternity-Briefing-women-and-equalities-committee-final.pdf	01/04/2020
BR_6	Final letter SOR-BMUS 22nd April Birthrights joint letter with National Maternity Voices, Sands, the Twins Trust and ARC https://www.birthrights.org.uk/wp-content/uploads/2020/04/Final-letter-SoR-BMUS-22-April.pdf	22/04/2020
BR_12	Letter to NHSE regarding ongoing visiting restrictions https://www.birthrights.org.uk/wp-content/uploads/2020/08/Letter-to-NHSE-re-ongoing-visiting-restrictions.pdf	20/08/2020

For peer review only

BR_14	Ongoing visitor restrictions in maternity services https://www.birthrights.org.uk/2020/09/14/ongoing-visitor-restrictions-in-maternity-services/	14/09/2020
BR_15	Template letter for ongoing visiting restrictions in maternity services https://www.birthrights.org.uk/template-letters/	14/09/2020
BR_16	Letter on restricting visitors – Sheffield teaching hospitals https://www.birthrights.org.uk/wp-content/uploads/2020/10/Letter-to-Sheffield-15th-October.pdf	15/10/2020
BR_17	"Partners are not visitors to maternity services" says Birthrights and partner organisations [joint letter on visiting restrictions to NHSE&I] https://www.birthrights.org.uk/wp-content/uploads/2020/10/Final-letter-to-NHSE-from-coalition-re-visiting-restrictions.pdf	20/10/2020
BR_19	Human rights charity calls for easing of visiting restrictions in maternity services press release https://www.birthrights.org.uk/wp-content/uploads/2020/08/Easing-of-visitor-restrictions-press-release-20-August-2020.pdf	20/08/202
BR_18	Human rights implications of changes to maternity services during the Covid-19 pandemic Birthrights briefing, July 2020 https://www.birthrights.org.uk/wp-content/uploads/2020/07/Birthrights-Covid-19-Human-Rights-Committee-Briefing-July-2020.pdf	Jul-20
BR_23	Long term impact of visiting restrictions "could be catastrophic" MPs, academics and campaigners warn NHS England CEO... https://www.birthrights.org.uk/2020/11/15/long-term-impact-of-visiting-restrictions-could-be-catastrophic-mps-academics-and-campaigners-warn-nhs-england-ceo/	15/11/2020
**ISUOG**		
ISUOG_6	ISUOG Safety Committee Position Statement: safe performance of obstetric and gynaecological scans and equipment cleaning in the context of COVID-19 https://www.isuog.org/resource/isuog-safety-committee-position-statement-safe-performance-of-obstetric-and-gynecological-scans-and-equipment-cleaning-in-the-context-of-covid-19.html	23/03/2020
ISUOG_5	ISUOG Consensus Statement on organization of routine and specialist obstetric ultrasound services in the context of COVID-19 https://www.isuog.org/resource/wiley-isuog-consensus-statement-on-organization-of-routine-and-specialist-obstetric-ultrasound-services-in-the-context-of-covid-19.html	31/03/2020

For peer review only

ISUOG_3	ISUOG Interim Guidance (COVID-19) during pregnancy & puerperium: Information for healthcare professionals – an update https://obgyn.onlinelibrary.wiley.com/doi/full/10.1002/uog.22061	01/05/2020
**NHS England**		
NHSE_1	Clinical guide for the temporary reorganisation of intrapartum maternity care during the coronavirus pandemic<br>https://madeinheene.hee.nhs.uk/Portals/0/Clinical%20guide%20for%20the%20temporary%20reorganisation<br>%20of%20intrapartum%20maternity%20care.pdf	09/04/2020
NHSE_2	Delivering midwifery intrapartum care where local COVID-19 escalation protocols are required to be enacted<br>https://www.nice.org.uk/media/default/about/covid-19/specialty-guides/delivering-midwifery-intrapartum-care-where-local-covid-19-escalation-protocols-are-required-to-be-enact.pdf	20/06/2020
NHSE_8	Framework to assist NHS trusts to reintroduce access for partners, visitors and other supporters of pregnant women in English maternity services http://allcatsrgrey.org.uk/wp/download/midwifery/par001599-framework-for-the-reintroduction-of-visitors-throughout-maternity-services-sep-2020.pdf	08/09/2020
NHSE_9	Letter to directors of nursing and heads of midwifery<br>(No longer available on website, available on request)	19/09/2020
NHSE_10	Lateral flow antigen test for pregnant women and support people: Frequently asked questions<br>https://www.england.nhs.uk/coronavirus/wp-content/uploads/sites/52/2020/12/C0972-lateral-flow-antigen-tests-for-maternity-faqs-december-2020.pdf	21/12/2020
NHSE_11	Supporting pregnant women using maternity services during the coronavirus pandemic: Actions for NHS providers Version 1, 14 December 2020<br>https://www.england.nhs.uk/coronavirus/wp-content/uploads/sites/52/2020/12/C0961-Supporting-pregnant-women-using-maternity-services-during-the-coronavirus-pandemic-actions-for-NHS-provi.pdf?fbclid=IwAR0w9hbJSbH1gYYPVhGtE8zRAvwd8PidPkPsEaDnhxzlSumFl7t6byshst8	14/12/2020
**RCOG**		
RCOG/RCM_1c	Coronavirus (COVID-19) Infection in Pregnancy Information for healthcare professionals Version 3<br>https://www.rcm.org.uk/media/3799/coronavirus-covid-19-infection-in-pregnancy-v3-20-03-18.pdf	18/03/2020
RCOG/RCM_1d	Coronavirus (COVID-19) Infection in Pregnancy Information for healthcare professionals Version 4<br>https://www.rcm.org.uk/media/3800/2020-03-21-covid19-pregnancy-guidance.pdf	21/03/2020

For peer review only

RCOG/RCM_1e	Coronavirus (COVID-19) Infection in Pregnancy Information for healthcare professionals Version 5<br>https://www.rcm.org.uk/media/3824/2020-03-28-covid19-pregnancy-guidance.pdf	28-03-202
RCOG/RCM_1f	Coronavirus (COVID-19) Infection in Pregnancy Information for healthcare professionals Version 6<br>http://www.e-lactancia.org/media/papers/2020-04-03-coronavirus-covid-19-infection-in-pregnancy.pdf	03/04/2020
RCOG/RCM_1g	Coronavirus (COVID-19) Infection in Pregnancy Information for healthcare professionals Version 7<br>https://www.sbmfc.org.br/wp-content/uploads/2020/04/2020-04-09-coronavirus-covid-19-infection-in-pregnancy.pdf	09/04/2020
RCOG_9	Guidance for rationalising early pregnancy services in the evolving coronavirus (COVID-19) pandemic<br>https://www.rcog.org.uk/globalassets/documents/guidelines/2020-05-15-guidance-for-rationalising-early-pregnancy-services-in-the-evolving-coronavirus-covid-19-pandemic.pdf	15/05/2020
RCOG_11	Framework for staffing of obstetrics and gynaecology units during the COVID-19 pandemic<br>https://www.rcog.org.uk/globalassets/documents/guidelines/2020-05-22-framework-for-staffing-of-obstetrics-and-gynaecology-units-during-the-covid-19-pandemic.pdf	22/05/2020
RCOG_6	Principles for the testing and triage of women seeking maternity care in hospital settings, during the COVID-19 pandemic<br>https://www.rcog.org.uk/globalassets/documents/guidelines/2020-05-29-principles-for-the-testing-and-triage-of-women-seeking-maternity-care-in-hospital-settings-during-the-covid-19-pandemic.pdf	29/05/2020
RCOG/RCM_1j	Coronavirus (COVID-19) Infection in Pregnancy Information for healthcare professionals Version 10 https://www.rcm.org.uk/media/4113/2020-06-04-coronavirus-covid-19-infection-in-pregnancy.pdf	04/06/2020
RCOG_7b	Guidance for antenatal and postnatal services in the evolving coronavirus (COVID-19) pandemic<br>https://www.rcog.org.uk/globalassets/documents/guidelines/2020-07-10-guidance-for-antenatal-and-postnatal.pdf	10/07/2020
RCOG_14	Restoration and Recovery: Priorities for Obstetrics and Gynaecology Version 2.1<br>https://www.rcog.org.uk/globalassets/documents/guidelines/2020-06-26-restoration-and-recovery---priorities-for-obstetrics-and-gynaecology.pdf	26/06/2020
RCOG/RCM_1k	Coronavirus (COVID-19) infection in pregnancy Information for healthcare professionals Version 11 https://www.rcog.org.uk/globalassets/documents/guidelines/2020-07-24-coronavirus-covid-19-infection-in-pregnancy.pdf	24/07/2020

RCOG_2	Guidance for provision of midwife-led settings and home birth in the evolving coronavirus (COVID-19) pandemic Version 1.3<br>https://www.rcog.org.uk/globalassets/documents/guidelines/2020-07-10-guidance-for-provision-of-midwife-led.pdf	10/07/2020
RCOG_4	Guidance for fetal medicine units (FMUs) in the evolving coronavirus (COVID-19) pandemic<br>https://www.rcog.org.uk/globalassets/documents/guidelines/2020-11-23-guidance-for-fetal-medicine-units.pdf	10/07/2020
RCOG_5	Guidance for antenatal screening and ultrasound in pregnancy in the evolving coronavirus (COVID-19) pandemic<br>https://www.rcog.org.uk/globalassets/documents/guidelines/2020-07-10-guidance-for-antenatal-screening.pdf	10/07/2020
RCOG_15	Joint RCOG & RCM Statement - Planning for Winter 2020/21: reducing the impact of COVID-19 on maternity services in the UK<br>https://www.rcog.org.uk/globalassets/documents/guidelines/2020-10-08-rcog_rcm_winter_secondwave_statement.pdf	08/10/2020
RCOG_7	Guidance for antenatal and postnatal services in the evolving coronavirus (COVID-19) pandemic<br>Version 3<br>https://www.rcog.org.uk/globalassets/documents/guidelines/2020-10-21-guidance-for-antenatal-and-postnatal-services-in-the-evolving-coronavirus-covid-19-pandemic-v3.pdf	21/10/2020
RCOG/RCM_1	Coronavirus (COVID-19) infection in pregnancy Information for healthcare professionals Version 12 https://www.rcm.org.uk/media/4383/2020-10-14-coronavirus-covid-19-infection-in-pregnancy-v12.pdf	14/10/2020
RCOG_17	RCOG responds to revised NHS coronavirus guidelines https://www.rcog.org.uk/en/news/pregnant-women-allowed-partner-support-at-all-times-in-updated-nhs-guidelines/	16/12/2020
**RCM**		
RCM_8	Induction of labour in a pandemic: A rapid analytic scoping review https://www.rcm.org.uk/media/3924/professional-clinical-briefing-no-7-intrapartum-care-with-symptomsmrd010520.pdf	04/04/2020
RCM_2	Optimising maternity services and maternal and newborn outcomes in a pandemic. A rapid analytic scoping review https://www.rcm.org.uk/media/3869/rapid-review-optimising-maternity-services-for-rcm-v4-8-april.pdf	08/04/2020

RCM_15	Guidance for midwives, student midwives and maternity support workers providing community-based care during the Covid-19 pandemic https://www.rcm.org.uk/media/3900/home-visit-guidance-for-midwives.pdf	09/04/2020
RCM_11	Rapid Analytic Review: Labour and Birth Companionship in a pandemic https://www.rcm.org.uk/media/3951/birth-companionship-in-a-pandemic-master-27-04-2020-002.pdf	27/04/2020
RCM_12	RCM Clinical Briefing Sheet: 'freebirth' or 'unassisted childbirth' during the COVID-19 pandemic https://www.rcm.org.uk/media/3923/freebirth_draft_30-april-v2.pdf	30/04/2020
RCM_16	Optimising mother-baby contact and infant feeding in a pandemic. Rapid analytic review https://www.rcm.org.uk/media/4096/optimising-infant-feeding-and-contact-rapid-review-19th-may-2020-submitted.pdf	19/05/2020
RCM_9	Face-coverings and care in labour for all women https://www.rcm.org.uk/media/4151/clinical-briefing-face-mask.pdf	01/07/2020
RCM_5	Antenatal Care for women without suspected or confirmed COVID-19 and living in a symptom free household https://www.rcm.org.uk/media/4180/clinical-guidance-briefing-one-antenatal-care-for-women-without-symptomsfinalv4-1.pdf	17/07/2020
RCM_4	RCM Briefing on Re-introduction of visitors to Maternity Units across the UK during the COVID-19 pandemic https://www.rcm.org.uk/media/4161/rcm-briefing-on-reintroduction-of-visitors-to-maternity-units-in-the-covid-pandemic-003.pdf	15/07/2020
RCM_3	Virtual consultations https://www.rcm.org.uk/media/4192/virtual-consultations-v20-24-july-2020-review-24-august-2020-1.pdf	24/07/2020
RCM_7	Guidance for intrapartum care for women with COVID-19 https://www.rcm.org.uk/media/4193/intrapartum-care_with-covid-19_20200724-v9-1.pdf	24/07/2020
RCM_10	RCM Clinical Briefing Sheet - Waterbirth during the COVID-19 Pandemic https://www.rcm.org.uk/media/4188/waterbirth-during-covid-19-july-20.pdf	19/07/2020
RCM_31	RCM & RCOG urge NHS England to publish guidance on partners during pregnancy https://www.rcm.org.uk/media-releases/2020/september/rcm-and-rcog-urge-nhs-england-to-publish-guidance-on-partners-during-pregnancy	08/09/2020

For peer review only

RCM_27	Birth partners https://www.rcm.org.uk/media/4339/birth-partners-v2-220920.pdf	20/09/2020
RCM_30	Leading Royal Colleges urge the NHS to learn lessons and avoid redeploying maternity staff ahead of second wave https://www.rcog.org.uk/en/news/leading-royal-colleges-urge-the-nhs-to-learn-lessons-and-avoid-redeploying-maternity-staff-ahead-of-second-wave/	30/09/2020
RCM_28	RCM letter to The Times responding to Times column on COVID rules and pregnancy https://www.rcm.org.uk/media-releases/2020/september/rcm-letter-to-the-times-responding-to-times-column-on-covid-rules-and-pregnancy/	02/10/2020
RCM_38	Partners and visitors<br>https://www.rcm.org.uk/partners-and-visitors/	Oct-20
RCM_39	Reduce infection risks to keep pregnant women and their babies safe, says RCM https://www.rcm.org.uk/media-releases/2020/october/reduce-infection-risks-to-keep-	02/11/2020
RCM_41	Midwives call for common sense on maternity visiting guidance<br>https://www.rcm.org.uk/media-releases/2020/december/midwives-call-for-common-sense-on-maternity-visiting-guidance/	15/12/2020
**Sands**		
Sands_1	Sands feedback: experiences of pregnant women, bereaved parents and families during the COVID-19 pandemic. No longer on website, available on request	19-05-2020 to<br>27/05/2020
Sands_1	Written evidence from SANDS to parliamentary committee Written evidence submitted by SANDS (DEL0092) https://committees.parliament.uk/writtenevidence/4073/html/	May-20
Sands_4	New NHS guidance on partners of pregnant women in England attending scans https://www.sands.org.uk/about-sands/media-centre/news/2020/09/new-nhs-guidance-partners-pregnant-women-england-attending	09/09/2020
**SoR**		
SoR_11	Obstetric ultrasound examinations during the COVID-19 pandemic.<br>Recording of NHS examinations and the use of private ultrasound clinics: A joint statement from the Society and College of Radiographers (SCoR), the Royal College of Midwives (RCM), the Royal College of Obstetricians and Gynaecologists (RCOG) and the British Medical Ultrasound Society (BMUS). https://archive.sor.org/sites/default/files/document-versions/obstetric_ultrasound_examinations_during_the_covid-19_pandemic_v2_0.pdf	24/04/2020

For peer review only

SoR_9	Ultrasound: Frequently asked questions (FAQs) version 1.7 previously on website – superseded by later versions (SoR & BMUS)	27/04/2020
SoR_10	Ultrasound: Frequently asked questions (FAQs) version 1.8 previously on website – superseded by later versions (SoR & BMUS)	21/05/2020
SoR_12	Obstetric ultrasound examinations during the COVID-19 pandemic.<br>Recording of NHS examinations and the use of private ultrasound clinics: A joint statement from the Society and College of Radiographers (SCoR), the Royal College of Midwives (RCM), the Royal College of Obstetricians and Gynaecologists (RCOG) and the British Medical Ultrasound Society (BMUS). https://archive.sor.org/sites/default/files/document-versions/obstetric_ultrasound_examinations_during_the_covid-19_pandemic_v2_0.pdf	27/05/2020
SoR_6	SoR demands NHS Trusts defend sonographers from populist attacks<br>https://www.sor.org/news/sor-demands-nhs-trusts-defend-sonographers-populist-attacks	04/11/2020
SoR_4	Ultrasound: Frequently asked questions (FAQs) version 1.10 previously on website – superseded by later versions (SoR & BMUS)	04/11/2020
SoR_7	Letter from SoR Resisting populist pressure to minimise the risks to ultrasound staff & patients.<br>Document supplied by SoR	06/11/2020
SoR_8	Webpage: New national guidance on partners attending antenatal scans<br>https://www.sor.org/news/obstetrics-and-gynaecology/new-national-guidance-on-partners-attending-antena	16/12/2020

For peer review only

t

For peer review only

Supplementary File 3: Documentary and interview data sources for the six key themes

Themes	***Documents***	***Stakeholders***
Postcode lottery of care	BR_8, BR_12, BR_17, BR_19, BR_18; RCOG/RCM_1e, RCOG_9, RCOG_15, RCM/RCOG_1l, RCOG_15, RCOG_17; Sands_1; RCM_5, RCM_7, RCM_31, RCM_38, RCM_39, RCM_41; NHSE_8, NHSE_9, NHSE_11; AIMS_2,AIMS_7, AIMS_8; SoR_2, SoR_4, SoR_5, SoR_6, SoR_7, SoR_8, SoR_9, SoR_10, SoR_11, SoR_12	7, 12, 13, 15, 17, 18, 20
Confusion and stress around 'rules'	BR_8, BR_12, BR_14, BR_16, BR_19; RCM_31; AIMS_7	12, 15, 19 20
Unintended Consequences	BR_1, BR_8, BR_12, BR_16, BR_17, BR_23; RCOG/RCM_1e, RCOG/RCM_1j , RCOG/RCM_1k, RCOG/RCM_1l; Sands_1; RCM_2, RCM_7, RCM_8, RCM_11, RCM_16 ; NHSE_1, NHSE_2, , NHSE_11; AIMS_8;	2, 4, 5, 8, 12, 13, 15, 17, 18, 19, 20
Need for flexibility	BR_8, BR_6, BR_15, BR_18; Sands_1; RCM_4, RCM_41; NHSE_8; AIMS_2, AIMS_8; SoR_12	3, 7, 10, 13, 17, 21
Acceptable time for support	BR_1, BR_6, BR_8, BR_16, BR_17, BR_18, BR_23; RCOG_7; Sands_1; RCM_3, RCM_8, RCM_11, RCM_27, RM_28; AIMS_2, AIMS _5, AIMS_8; SoR_2, SoR_4, SoR_5, SoR_9, SoR_10, SoR_11, SoR_12	5, 7, 13, 15, 16, 17, 18, 19, 20, 21, 26
Loss of human rights for gain in infection control	BR_16, BR_17, BR_19, BR_23, BR_8, BR_18; RCOG/RCM_1g , RCOG/RCM_1c, RCOG/RCM_1e, RCOG/RCM_1f , RCOG_9, RCOG_6; RCM_11, RCM_30, RCM_28,RCM_41, RCM_39, RCM-38, RCM_11; NHSE_9, NHSE_10; SoR_2, SoR_6, SoR_5, SoR_8, SoR_7, SoR_4, SoR_9, SoR_10, SoR_11, SoR_12; ISUOG 5, ISUOG_6	3, 5, 11, 14, 17

For peer review only

COREQ (COnsolidated criteria for REporting Qualitative research) Checklist

A checklist of items that should be included in reports of qualitative research. You must report the page number in your manuscript where you consider each of the items listed in this checklist. If you have not included this information, either revise your manuscript accordingly before submitting or note N/A.

Topic	Item No.	Guide Questions/Description	Reported on Page No.
**Domain 1: Research team and reflexivity**			
Personal characteristics			
Interviewer/facilitator	1	Which author/s conducted the interview or focus group?	7
Credentials	2	What were the researcher's credentials? E.g. PhD, MD	Title
Occupation	3	What was their occupation at the time of the study?	Title/7
Gender	4	Was the researcher male or female?	7
Experience and training	5	What experience or training did the researcher have?	Title/7
Relationship with participants			
Relationship established	6	Was a relationship established prior to study commencement?	7
Participant knowledge of the interviewer	7	What did the participants know about the researcher? e.g. personal goals, reasons for doing the research	7
Interviewer characteristics	8	What characteristics were reported about the inter viewer/facilitator? e.g. Bias, assumptions, reasons and interests in the research topic	7
**Domain 2: Study design**			
Theoretical framework			
Methodological orientation and Theory	9	What methodological orientation was stated to underpin the study? e.g. grounded theory, discourse analysis, ethnography, phenomenology, content analysis	5
Participant selection			
Sampling	10	How were participants selected? e.g. purposive, convenience, consecutive, snowball	6
Method of approach	11	How were participants approached? e.g. face-to-face, telephone, mail, email	6
Sample size	12	How many participants were in the study?	7
Non-participation	13	How many people refused to participate or dropped out? Reasons?	7
Setting			
Setting of data collection	14	Where was the data collected? e.g. home, clinic, workplace	6
Presence of non-participants	15	Was anyone else present besides the participants and researchers?	N/A
Description of sample	16	What are the important characteristics of the sample? e.g. demographic data, date	7
Data collection			
Interview guide	17	Were questions, prompts, guides provided by the authors? Was it pilot tested?	6/Supp File
Repeat interviews	18	Were repeat inter views carried out? If yes, how many?	N/A
Audio/visual recording	19	Did the research use audio or visual recording to collect the data?	6
Field notes	20	Were field notes made during and/or after the inter view or focus group?	N/A
Duration	21	What was the duration of the inter views or focus group?	6
Data saturation	22	Was data saturation discussed?	6
Transcripts returned	23	Were transcripts returned to participants for comment and/or	6

For peer review only

For peer review only - http://bmjopen.bmj.com/site/about/guidelines.xhtml

Topic	Item No.	Guide Questions/Description	Reported on Page No.
		correction?	
**Domain 3: analysis and findings**			
Data analysis			
Number of data coders	24	How many data coders coded the data?	6
Description of the coding tree	25	Did authors provide a description of the coding tree?	11
Derivation of themes	26	Were themes identified in advance or derived from the data?	6
Software	27	What software, if applicable, was used to manage the data?	Excel/6
Participant checking	28	Did participants provide feedback on the findings?	6
Reporting			
Quotations presented	29	Were participant quotations presented to illustrate the themes/findings? Was each quotation identified? e.g. participant number	12-20
Data and findings consistent	30	Was there consistency between the data presented and the findings?	12-20
Clarity of major themes	31	Were major themes clearly presented in the findings?	12-20
Clarity of minor themes	32	Is there a description of diverse cases or discussion of minor themes?	All data includ

Developed from: Tong A, Sainsbury P, Craig J. Consolidated criteria for reporting qualitative research (COREQ): a 32-item checklist for interviews and focus groups. *International Journal for Quality in Health Care*. 2007. Volume 19, Number 6: pp. 349 – 357

Once you have completed this checklist, please save a copy and upload it as part of your submission. DO NOT include this checklist as part of the main manuscript document. It must be uploaded as a separate file.

For peer review only

BMJ Open

Companionship for women/birthing people using antenatal and intrapartum care in England during COVID-19: A mixed-methods analysis of national and organisational responses and perspectives

Journal:	*BMJ Open*
Manuscript ID	bmjopen-2021-051965.R2
Article Type:	Original research
Date Submitted by the Author:	24-Nov-2021
Complete List of Authors:	Thomson, Gillian; University of Central Lancashire, MAINN, School of Community Health & Midwifery<br>Balaam, Marie-Claire; University of Central Lancashire, ReaCH, School of Community Health & Midwifery<br>Nowland (Harris), Rebecca; University of Central Lancashire, MAINN, School of Community Health & Midwifery<br>Crossland, Nicola; University of Central Lancashire, MAINN, School of Community Health & Midwifery<br>Moncrieff, Gill; University of Central Lancashire, School of Community Health & Midwifery<br>Heys, Stephanie; North West Ambulance Service NHS Trust<br>Sarian, Arni; University of Central Lancashire, School of Medicine<br>Cull , Joanne; University of Central Lancashire, School of Community Health & Midwifery<br>Topalidou, Anastasia; University of Central Lancashire, ReaCH, School of Community Health & Midwifery<br>Downe, Soo; University of Central Lancashire, ReaCH, School of Community Health & Midwifery
**Primary Subject Heading**:	Obstetrics and gynaecology
Secondary Subject Heading:	Qualitative research, Health services research, Public health
Keywords:	GYNAECOLOGY, Organisational development < HEALTH SERVICES ADMINISTRATION & MANAGEMENT, Risk management < HEALTH SERVICES ADMINISTRATION & MANAGEMENT, Public health < INFECTIOUS DISEASES, QUALITATIVE RESEARCH

SCHOLARONE™ Manuscripts

I, the Submitting Author has the right to grant and does grant on behalf of all authors of the Work (as defined in the below author licence), an exclusive licence and/or a non-exclusive licence for contributions from authors who are: i) UK Crown employees; ii) where BMJ has agreed a CC-BY licence shall apply, and/or iii) in accordance with the terms applicable for US Federal Government officers or employees acting as part of their official duties; on a worldwide, perpetual, irrevocable, royalty-free basis to BMJ Publishing Group Ltd ("BMJ") its licensees and where the relevant Journal is co-owned by BMJ to the co-owners of the Journal, to publish the Work in this journal and any other BMJ products and to exploit all rights, as set out in our licence.

The Submitting Author accepts and understands that any supply made under these terms is made by BMJ to the Submitting Author unless you are acting as an employee on behalf of your employer or a postgraduate student of an affiliated institution which is paying any applicable article publishing charge ("APC") for Open Access articles. Where the Submitting Author wishes to make the Work available on an Open Access basis (and intends to pay the relevant APC), the terms of reuse of such Open Access shall be governed by a Creative Commons licence – details of these licences and which Creative Commons licence will apply to this Work are set out in our licence referred to above.

Other than as permitted in any relevant BMJ Author's Self Archiving Policies, I confirm this Work has not been accepted for publication elsewhere, is not being considered for publication elsewhere and does not duplicate material already published. I confirm all authors consent to publication of this Work and authorise the granting of this licence.

For peer review only

Title: Companionship for women/birthing people using antenatal and intrapartum care in England during COVID-19: A mixed-methods analysis of national and organisational responses and perspectives

Authorship

*Gill Thomson, Professor in Perinatal Health, School of Community Health & Midwifery, University of Central Lancashire, Preston, UK. PR1 2HE. GThomson@uclan.ac.uk

Marie-Clare Balaam, School of Community Health & Midwifery, University of Central Lancashire, Preston, UK.

Rebecca Nowland, School of Nursing, University of Central Lancashire, Preston, UK.

Nicola Crossland, School of Community Health and Midwifery, University of Central Lancashire, Preston, UK.

Gill Moncrieff, School of Community Health & Midwifery, University of Central Lancashire, Preston, UK.

Stephanie Heys, Consultant Midwife, North West Ambulance Service, Bolton, UK.

Arni Sarian, School of Medicine, University of Central Lancashire, Preston, UK.

Joanne Cull, School of Community Health & Midwifery, University of Central Lancashire, Preston, UK.

Anastasia Topalidou, School of Community Health & Midwifery, University of Central Lancashire, Preston, UK.

Soo Downe, School of Community Health & Midwifery, University of Central Lancashire, Preston, UK.

And the ASPIRE-COVID19 Collaborative Group

* Corresponding author

Word count: 6,760

ASPIRE-COVID19 Collaborative Group

Co-investigators
Soo Downe; University of Central Lancashire, George Ellison; University of Central Lancashire, Alan Fenton; Newcastle upon Tyne Hospitals NHS Foundation Trust, Alexander Heazell; University of Manchester, Ank de Jonge; Amsterdam University Medical Center, Carol Kingdon; University of Central Lancashire, Zoe Matthews; University of Southampton, Alexandra Severns; NHS England and NHS Improvement North West, Gill Thomson; University of Central Lancashire, Anastasia Topalidou; University of Central Lancashire, Alison Wright; Royal Free Teaching Hospital in London

For peer review only - http://bmjopen.bmj.com/site/about/guidelines.xhtml

Research Staff

Naseerah Akooji; University of Central Lancashire, Marie-Clare Balaam; University of Central Lancashire, Jo Cull; University of Central Lancashire, Lauri van den Berg; Amsterdam University Medical Center, Nicola Crossland; University of Central Lancashire, Claire Feeley; University of Central Lancashire, Beata Franso; Amsterdam University Medical Center, Steph Heys; University of Central Lancashire, Gill Moncrieff; University of Central Lancashire, Rebecca Nowland; University of Central Lancashire, Arni Sarian; University of Central Lancashire

Steering committee

Maria Booker; Birthrights, Jane Sandall; Kings College London, Jim Thornton (chair); The University of Nottingham, Tisian Lynskey-Wilkie; University of Central Lancashire, Vanessa Wilson; Lancashire and South Cumbria Local Maternity System

Stakeholder Group

Rebecca Abe and Tinuke Awe; FivexMore, Toyin Adeyinka; MVP BAME group, Ruth Bender-Atik; The Miscarriage Association, Lia Brigante; RCM, Rebecca Brione; Birthrights, Franka Cadée; International Confederation of Midwives, Elizabeth Duff; Expert, postnatal care, Tim Draycott; Royal College of Obstetricians and Gynaecologists, Duncan Fisher; Fathers included/Family included/ the Family Initiative, Annie Francis; Independent Midwifery Advisor, Arie Franx; Erasmus MC, Lucy Frith; University of Liverpool, Louise Griew; National Maternity Voices, Clea Harmer; Sands, Caroline Homer; Burnet Institute, Australia, Marian Knight; National Perinatal Epidemiology Unit, Amali Lokugamage; Whittington Health NHS Trust/ University College London, Amanda Mansfield; London Ambulance Service Trust, Neil Marlow; University College London, Trixie Mcaree; NHS England, David Monteith; Grace in Action, Keith Reed; Twins Trust, Yana Richens; UCL & City University, Lucia Rocca-Ihenacho; Midwifery Unit Network, Mary Ross-Davie; Royal College of Midwives Scotland, Seana Talbot; BirthWise NI, Myles Taylor; British Maternal and Fetal Medicine Society, Maureen Treadwell; Birth Trauma Association

For peer review only - http://bmjopen.bmj.com/site/about/guidelines.xhtml

ABSTRACT

Objectives: To explore stakeholders' and national organisational perspectives on companionship for women/birthing people using antenatal and intrapartum care in England during COVID-19, as part of the ASPIRE COVID-19 study.

Setting: Maternity care provision in England.

Participants: Interviews were held with 26 national governmental, professional, and service-user organisation leads (July-Dec 2020). Other data included public-facing outputs logged from 25 maternity Trusts (Sept/Oct 2020) and data extracted from 78 documents from eight key governmental, professional, and service-user organisations that informed national maternity care guidance and policy (Feb-Dec 2020).

Results: Six themes emerged: '*Postcode lottery of care*' highlights variations in companionship and visiting practices between trusts/locations, '*Confusion and stress around 'rules''* relates to a lack of and variable information concerning companionship/visiting. '*Unintended consequences'* concerns the negative impacts of restricted companionship or visiting on women/birthing people and staff, '*Need for flexibility'* highlights concerns about applying companionship and visiting policies irrespective of need, ''*Acceptable' time for support'* highlights variations in when and if companionship was 'allowed' antenatally and intrapartum; and '*Loss of human rights for gain in infection control'* emphasizes how a predominant focus on infection control was at a cost to psychological safety and human rights.

Conclusions: Policies concerning companionship and visiting have been inconsistently applied within English maternity services during the COVID-19 pandemic. In some cases, policies were not justified by the level of risk, and were applied indiscriminately regardless of need. There is an urgent need to determine how to sensitively and flexibly balance risks and benefits and optimise outcomes during the current and future crisis situations.

Strengths and limitations of this study

- This is the first paper to consider links between policy and practice in companionship and visiting in maternity care during the COVID-19 pandemic.
- Data triangulation across national level policy documents, interviews with key stakeholders and public facing Trust documentation provides nuanced and context related perspectives on why and how companionship and visiting was impacted.

For peer review only - http://bmjopen.bmj.com/site/about/guidelines.xhtml

- Practice related issues were collected from 25 Trusts websites and social media-based public facing information, which may or may not reflect actual care practices.
- The paper focuses on antenatal and intrapartum care, with postnatal (including neonatal) care to be the focus of future publications.
- The study does not include information directly reported by parents or healthcare professionals.

INTRODUCTION

In many cultures around the world, pregnancy is framed as a social event rather than a clinical condition[1-3]. Even where pregnancy, labour and birth are framed as medically risky, social support is expected. Such support, generally framed as companionship, is usually provided through ongoing family and community relationships, often by female relatives and friends, or community members[4]. As maternity care has become more hospital based, the power to determine who should accompany women/birthing people in clinics and facility settings has shifted to the organisation, and its employees[5]. In the general hospital setting, the public is usually divided into 'patients' or 'visitors'. During the early decades of mass hospitalisation for antenatal care and birth in the UK, accompaniment for pregnant, childbearing, and postnatal women/birthing people was either disallowed, or conceptualised as 'visiting', and restricted to specific visiting hours. These limitations were justified on the grounds of infection control, overcrowding, privacy for others, and defence from potential litigation if the accompanying companions witness activities they perceive to be negligent or dangerous[6-8].

Companionship in maternity care is an evidence-based practice with documented benefits and care experiences and clinical outcomes [1] and has been associated with four key attributes: informational support, advocacy, practical support, and emotional support[1]. Qualitative studies show that most women/birthing people value companionship, and global guidelines strongly emphasis the need to support and facilitate women's choice to be accompanied throughout the maternity journey [9]. Though restrictions persist in some health economies around the world, companionship and visiting policies in maternity clinics and hospitals in the UK have become increasingly liberal over the last 40 years.
A survey published in 2013 found that over half of fathers/co-parents attended at least one antenatal check, and that 'almost all' were present for ultrasound screening in pregnancy, and for labour[10]. In 2019 97% of women/birthing people in England said that their partner or

For peer review only - http://bmjopen.bmj.com/site/about/guidelines.xhtml

someone else close to them was involved as much as they wanted them to be during labour and birth[11]. This is likely related to more inclusive policies as well as consumer demand.

The COVID-19 pandemic brought the issue of both visitors and companions in health facilities worldwide into sharp focus[12]. In terms of maternity care, there have been anecdotal accounts of wide variations concerning if and whether women/birthing people have been permitted companionship or visitor rights at various points throughout the maternity episode, both between countries, and across different care providers within countries. Concerns about women/birthing people being alone for antenatal contacts, for ultrasound scans (especially when there is bad news), during labour and birth have been widespread and global in media reports (for example: [12-14]).

To understand how companionship and visiting in maternity care during COVID-19 was operationalised organisationally in England during antenatal and intrapartum care, this paper presents an analysis of relevant national level policy documents; interviews with key stakeholders; and a review of public facing information produced by 25 purposively selected maternity providers in England.

METHODS

This mixed-methods study is part of a larger mixed-methods, observational, multi-site comparative research project - Achieving Safe and Personalised maternity care In Response to Epidemics (ASPIRE COVID-19 UK).

Data relating to companionship and visiting during the antenatal and intrapartum period in maternity care were extracted from national policy level documents and interviews with national stakeholders and mapped to analysis of public-facing communication channels from 25 Trusts (maternity care organisations). The Trusts were selected using maximum variation sampling, based on macro-level factors impacting upon health inequalities (area level deprivation reported in the English Indices of Deprivation[15]), meso-level considerations relating to the organisation (Care Quality Commission (CQC) rating and maternal and neonatal mortality figures), and micro-level factors including aspects such as parity and access to care identified within national documents.

Data collection

For peer review only - http://bmjopen.bmj.com/site/about/guidelines.xhtml

Documentary review

Guidelines, position papers, and reports relating to maternity care were collected prospectively between February and December 2020 from key governmental, professional, and service-user organisations. The organisations were identified as those who provide guidance, campaign and/or advocate for national practice and policy in relation to maternity care. These documents were sourced via organisation-based websites and from key stakeholders involved in the ASPIRE study. All documents that concerned maternity care provision were reviewed with any/all data in relation to companionship and visiting during antenatal and intrapartum care extracted and logged in Excel files.

Trust-level public facing communication about maternity service provision

Data related to companionship and visiting in pregnancy, labour and birth were extracted from Trust websites and Trust-related Twitter, Facebook, and Instagram feeds, between September and October 2020. We extracted information on the format of information presented, access to companionship or visiting antenatally (including ultrasound), and during labour and birth (including induction of labour). We also extracted information that discussed personalisation, organizational response to specific additional needs, Trust response to national guidance, rationale for decisions about companionship/visiting rules, and any additional information on companionship/visiting in the context of COVID-19.

Interviews

Purposive sampling was used to recruit individuals from relevant national governmental, professional, and service-user organisation leads involved in maternity care. Key individuals were identified by project and advisory teams and via snowballing. All participants were approached by email and provided with an information sheet about the study. A consent form was either completed and returned via email, or the consent process was audio-recorded at the start of the interview and stored separately from the interview recording. Semi-structured interviews were held July-December 2020 via videoconferencing (using Microsoft Teams). Interviews lasted between 45-60 minutes, were audio recorded and transcribed in full. The interview schedule (see Supplementary File 1) explored stakeholders' perceptions and experiences of what, why and how changes in maternity care delivery had been made during the pandemic, how changes had been monitored and assessed, and their views on facilitators and barriers to those changes. A total of 50 individuals were approached to participate and interviews were held with 26 stakeholders. While some stakeholders did not respond to the

For peer review only - http://bmjopen.bmj.com/site/about/guidelines.xhtml

request, others provided names of individuals who they considered would be more suitable. Recruitment was not based on saturation but rather was designed to ensure that we included representation from all key organisations and from individuals that were considered to offer important insights into maternity care delivery.

Ethics approval

Ethics approval to undertake the interviews was obtained from the Health ethics sub-committee from the lead author's institution (project no: 0079). Participants were asked if they would like to check any public facing quotes prior to publication as part of the consent process, with quotes returned to seven participants for feedback, and four required slight changes to be made. All feedback either involved minor grammatical changes; ensuring the quote was being used in the correct context, or, in one case, a change to job title.

Data analysis

All data analysis was undertaken by hand using Excel. Data analysis for the different forms of data (interviews, documents, Trust level data) were initially undertaken separately (by RN, MCB and NC respectively) before being combined into six key themes using a descriptive content analysis approach[16] led by GT. The stages of analysis were as follows:

a) Trust-level data were mapped to different aspects of care (e.g. appointments, ultrasound, induction, labour/birth) to identify any variations in maternity service companionship/visiting policies.

b) All interview and documentary data that concerned companionship/visiting were extracted and then read on a line-by-line basis to inductively identify meaning units - *'the constellation of words or statements that relate to the same central meaning'*([17]p.106). The meaning units were 'manifest' in terms of identifying the visible and salient components in the data.

c) The meaning unit labels, and associated data were then grouped and synthesised into themes. This process is referred to as *'abstraction'* by emphasising descriptions at a *'higher logical level'*([17]p.106);

d) In the final phase, the Trust level data were integrated into the themes for reporting purposes.

Four members of the team (GT, RN, MCB, NC) were involved in data analysis, and the final themes were agreed by all named authors.

For peer review only - http://bmjopen.bmj.com/site/about/guidelines.xhtml

Public and Patient Involvement

This study was funded under a rapid response call and while no formal PPI involvement in the original design of the study, UK service-user leads (Maternity Voices Partnership) and members of national charities and from service-user organisations are involved as co-investigators, steering group and advisory members for the ASPIRE project, to ensure that service-user inputs have been considered at every stage of the study.

Reflexivity

The authors and members of the collaborating group associated with this study are from a range of academic and clinical backgrounds including midwifery, psychology, obstetrics, neonatology, sociology, and social statistics. All the authors are female, and the four interviewers are experienced in undertaking qualitative interviews. One had previously collaborated with some of the interviewees. All authors believe that women/birthing people highly value companionship during key moments in their maternity care, that the priority for policies should be for supporting and facilitating companionship, if desired, and that, for many fathers/co-parents, being present is more than just being a visitor or a supporter. From her psychological background, RN also believes that companions other than fathers/co-parents play a significant role during childbirth in promoting psychological well-being. As midwives, SD, GM, JC, and SH view companionship for women/birthing people throughout labour and during antenatal care as a normative practice.

RESULTS

All the twenty-six interview participants held a national and/or strategic role in midwifery (n=9), obstetrics (n=1), neonatology (n=1), anaesthesia (n=1), radiography/sonography (n=2) or as an NHS improvement lead (n=1). One was from a doula organisation, three were from the Maternity Voice Partnership (a NHS working group comprising lay members and professionals dedicated to improving maternity care), five were from national charities (focused on birth trauma, premature/sick infants, stillbirth, miscarriage, multiple births) and two service-user organisations that campaign and advocate for maternity care improvements.

For peer review only - http://bmjopen.bmj.com/site/about/guidelines.xhtml

All the documents were collected from eight governmental, professional, and service-user sources (Box 1), with a total of 78 documents providing evidence for the paper (see Supplementary File 2 for full details/references).

Box 1: Organisations included for documentary analysis

Sands (national charity focused on stillbirth)
AIMS (service-user organisation dedicated to improving maternity care)
Royal College of Midwives (RCM)
Royal College of Obstetricians and Gynaecologists (RCOG)
Society of Radiographers (SoR)
International Society of Ultrasound in Obstetrics and Gynaecology (ISUOG)
NHS England (NHSE)
Birthrights (BR) (service-user organisation dedicated to improving maternity care)

For peer review only

The public facing data logged between September and October 2020 demonstrated a very wide range of policies and practices between the 25 included Trusts for companionship/visiting during four specific maternity care episodes (antenatal scanning; antenatal appointments; antenatal ward stays; and intrapartum) (see Table1). While this could be explained by different COVID-19 infection exposure rates, this may not explain variation between Trusts in the same region.

Table 1: Public facing information on maternity service companionship and visiting policies during antenatal and intrapartum care in 25 English maternity Trusts, September to October 2020

	**Antenatal**			**Intrapartum**		
	**Ultrasound**	**Antenatal appointments**	**Antenatal ward**	**Number of birth partners**	**Timing**	**Induction of labour**
Greater London 1	Unaccompanied	Unaccompanied	Booked time slot	One	Throughout labour	Partner allowed if assessed as needing support
Greater London 2	Partner allowed (20 weeks) *	Unaccompanied	2-6pm	Two	Not specified	No information

For peer review only - http://bmjopen.bmj.com/site/about/guidelines.xhtml

Greater London 3	Partner allowed (20 weeks)	No details	No visitors	One (two if additional need identified)	Established labour	No information
Greater London 4	Unaccompanied	Unaccompanied	No visitors	One	Established labour	No information
Greater London 5	Partner allowed	Unaccompanied	Daytime	One (two if need identified)	Not specified	No information
Greater London 6	Partner allowed	No details	Daytime	One	Throughout labour	Partner allowed daytime only
South East 1	Unaccompanied	One companion	Booked time slot	One	Not specified	No information
South East 2	Partner allowed (12 and 20 weeks)	No details	Daytime	Two	Not specified	Partner allowed
South East 3	Unaccompanied	Unaccompanied	Daytime	One	Throughout	No information
South West 1	Partner allowed (12 and 20 weeks)	Unaccompanied	Booked time slot	One	Not specified	Daytime only
South West 2	Partner allowed (20 weeks)	Unaccompanied	No visitors	One	Throughout	Partner not allowed until labour has started
South West 3	Partner allowed (20 weeks)	No details	No information	One	Throughout	Partner allowed daytime or if additional support needed
West Midlands 1	Unaccompanied	Unaccompanied	No visitors	One	Not specified	No information
West Midlands 2	Unaccompanied	Unaccompanied	No information	One	Not specified	Partner allowed if language/communicatio n needs
East Midlands 1	Partner allowed (12 and 20 weeks)	Unaccompanied	No information	One	Throughout	No information
East of England 1	Partners allowed	No information	Booked time slot	One	Throughout	No information
East of England 2	Partner allowed (20 weeks)	Unaccompanied	No information	One	Established labour	Partner not allowed until labour has started
East of England 3	Partner allowed (12 and 20 weeks)¶	Unaccompanied	2-6 pm	One	Not specified	No information
Yorkshire and Humber 1	Partners allowed	No details	No visitors	One	Not specified	Partner not allowed until labour has started
Yorkshire and Humber 2	Partner allowed (12 weeks)	No details	Booked time slot between 1-5pm	One	Throughout	No information
North West 1	Partner allowed (12 and 20 weeks)	Unaccompanied	Booked time slot	One	Throughout	One partner allowed

For peer review only - http://bmjopen.bmj.com/site/about/guidelines.xhtml

North West 2	Partners allowed	Unaccompanied	No information	Two	Not specified	No information
North West 3	Partner allowed (20 weeks)	Unaccompanied	No information	Two	Not specified	No information
North East 1	Partner allowed (12 and 20 weeks)†	Unaccompanied	No information	One	Not specified	No information
North East 2	Partners allowed	No details	No information	One	Not specified	No information

Note: The term 'partner' is used in this table as this, and 'birth partner', were the most commonly used terms to refer to an antenatal or intrapartum companion.

* Phone call offered if clinical concerns

¶ Video offered of a small section at the end of scan

† Phone call offered (end of scan)

Details of themes

Overall, six themes emerged from synthesising the meaning units from the documentary and interview data sets (see Table 2). (Further details of the documents and interviews that generated data for each theme are provided in Supplementary File 3).

Table 2: Theme and associated meaning units from the documentary and interview data

**Themes**	**Meaning units**	
	**Documentary data**	**Interview data**
**Postcode lottery of care**	Different policies used in local situations	Trust dictates rationale for decision making
	Tensions between national and local policy and practise	Differences between trusts resulting in geographical variations
**Confusion and stress around rules**	Concern over transparency, clarity and rationale for decision making	Confusion with rules leads to frustration
		Confusions between staff about the rules
**Unintended consequences**	The need for companions as they improve wellbeing and outcomes for women/birthing people (and the negative impact of not having companions)	Lack of companionship created a distressing and frightening experience for women/birthing people

For peer review only - http://bmjopen.bmj.com/site/about/guidelines.xhtml

	The unintended consequences of lack of/restrictions on companions	Lack of support for women/birthing people from companions
	The presence of companions supports staff	Increased work burden for staff
	The need to provide alternative support for women/birthing people if companion not present	Being alone when getting bad news at the scan
**Need for flexibility**	Need for consideration of women/birthing people who are identified to be particularly vulnerable, marginalised or need extra support (e.g. due to ethnicity, language issues, baby loss)	Maternity services should be an exception
	The need to look at situations on a case by case basis to support personalised care	Rules should be applied flexibly to meet the needs of vulnerable women/birthing people
		Importance of being flexible with rules when babies die
**Acceptable time for support**	The use of virtual means to replace physical companionship in antenatal scans	Ultrasound - rigidity and lack of flexibility
	Concerns over lack of companionship in early labour and for women/birthing people who are induced and need for support at this time	Knowing when to bring the companion in with active labour - impacts of being in prolonged labour alone
	Concerns over women/birthing people only allowed support in 'active' labour and how this is determined.	Issues around companionship at the time of induction
**Loss of human rights for gain in infection control**	The balance between risk of transmission and the risks to women/birthing people	Expectation that women's/birthing people's rights around childbirth needed to be sacrificed for safety
	The assertion of women's/birthing people's (and companions) human rights as the basis of companionship	Limited focus on safety, centred on infection control

Postcode lottery of care

The notion of a postcode lottery of maternity service provision gained traction in the media over the summer of 2020[18]. Concerns were reflected in national documents from almost all included organisations, the stakeholder interviews, and reflected in Trust level responses. Variation was justified by some organisations as a reaction to local need, for example: *'restrictions on other visitors should follow hospital policy and national guidance'* (RCM_7)

For peer review only - http://bmjopen.bmj.com/site/about/guidelines.xhtml

and 'a*ll staff should work to the same local policy, to provide a consistent service to women'* (SoR_5). The caveat to most guidance was that any policies needed to be re-addressed in the event of local spikes in COVID-19 cases or following local risk assessment, to ensure a '*consistent service to women'* (SoR_2):

> But it's guidance, and every hospital will make its own decisions and to a certain extent, they will have to because the physicality, the layout, the facilities that they have in hospitals will differ. You know, there's not much room in the waiting area if the corridors are very narrow [so] that the people can't have a two-metre distance. (Stakeholder 20, National charity)

Some Trusts were identified as having '*gone out of their way to ensure their services remain family-centred'* (BR_14), whereas in others, partners or other companions of choice were unable to attend any antenatal appointments or scans (BR_16). NHS England released guidance in September 2020 intended to assist Trusts to reintroduce access for companions (NHSE_8). Some organisational responses claimed that this led to '*some Trusts starting to backtrack and reduce restrictions in maternity services'* (BR_17), while other Trusts continued to impose restrictions (BR_18) and often without a clear rationale for these variations:

> And that's the problem. You… because down the street, down the road, you could get a very warm, empathetic ultrasonographer who says, of course, yeah, I realize how difficult is. You know, it doesn't take much, and we just have lost it because people are stressed and there's lots of reasons for it, but it's just not good enough. (Stakeholder 18, Midwifery – Strategic role)

Confusion and stress around 'rules'

Concern over a lack of clarity in decision making and changes in policies around companionship/visiting were highlighted. For instance, letters from Birthrights to maternity leads (e.g. BR_12, BR_16, BR_19) repeatedly emphasised the need for clear reasons, evidence, and justification as to why decisions were being made. Concerns included that Trusts *'acted too quickly to withdraw services'* and *'decision-making has not always been proportionate or transparent'* (BR_18). While most Trusts made some reference to infection risk as the rationale for the restrictions, a sizeable minority (9/25) did not. Many Trusts offered no rationale as to why partners could attend some appointments but not others, e.g.:

For peer review only - http://bmjopen.bmj.com/site/about/guidelines.xhtml

> One birth partner may attend for the 20-week anomaly scan only. Women to attend all other scan appointments unaccompanied (South West 2).

Birthrights and some stakeholders highlighted a failure to communicate local restrictions in a timely manner, compounded by rules changing rapidly and difficulties in communicating these changes widely and consistently to large numbers of healthcare professionals. This confusion about the rules was also compound by changeovers of staff and communication between different teams in some areas – *'there's been no consultation with sonographers in terms of risk assessments* […] *or changes in practice'* (Stakeholder 26, Radiographer), and services being provided by staff from outside the maternity team.

While some stakeholders noted that individual Trusts responded to this confusion by using a range of public communications, data from the 25 maternity Trusts found that less than half (10/25) had a consistent message about companionship or visiting across different channels. Parent frustration reported by stakeholders also related to how the rules for companions and visiting seemed at odds with the social distancing rules outside of the hospital context:

> In the middle of lockdown, it was 'we don't like it, but we know you're keeping us safe'. Now 'it's we don't like it and I don't see how you're keeping us more safe doing this because I can meet my partner in the pub, but he can't come to my scan. I can do this, but I can't do that'. Yes. So, it's more of an angry mood now. (Stakeholder 12, Maternity Voices Partnership)

In some of the documents by Birthrights (e.g. BR_14; BR_16) and RCM (i.e. RCM_31) they called for the harm caused by restricted access by companions to be properly and transparently considered within the decision-making processes:

> We would be grateful if you could publish or send us the risk assessment that quantifies the increased risk of spreading COVID within the unit (despite PPE and other mitigating factors, and the fact that most partners are from the same household) caused by relaxing restrictions, and weighs this against the known harms to pregnant women, birthing people and their families from keeping the current restrictions in place. (BR_16)

For peer review only - http://bmjopen.bmj.com/site/about/guidelines.xhtml

Unintended Consequences

Organisations including RCM, RCOG, NHS England and Birthrights highlighted that having trusted companions throughout labour and birth is linked to improved outcomes, and a lack of companionship was associated with increased need for pharmacological or other interventions (AIMS_8, BR_23). This included perceived impacts on the labour process due to, for example, [increased] *'demand for epidurals'* (RCM_8). Alongside the obvious fear and anxiety of infection, organisations and stakeholders highlighted concerns about women/birthing people feeling '*petrified'* (Stakeholder 15, Midwifery – Strategic role) or not accessing maternity care, '*due to the women's preferred birth partner not being allowed to accompany her*' (RCM_2). There were also concerns of companions feeling *'unsupported and uncared for'* (Stakeholder 20, National charity) due to being unable to be with the woman/birthing person when they heard bad news (during ultrasound) or missing the birth due to *'being told that they should wait in the car park or something'* (Stakeholder 7, Service-user organisation). All but four Trust websites contained messages of empathy regarding the restrictions, sometimes alongside expressions of regret and/or justifications for their necessity: '*We understand the restrictions we have had in place over recent months have been particularly hard for pregnant women and their families*' (North West 1). Concerns were also expressed by stakeholders and within documents by Sands and Birthrights, towards women/birthing people who had experienced prior baby loss or who may receive bad news alone during the scan:

> Women are being asked to attend scans alone, with many sharing frustrations that they cannot video link to their partners. These very vulnerable women tell us they are concerned about having to attend stressful antenatal appointments and scans alone. While units are being encouraged to consider facilitating women to take a video clip at the end of an appointment, this is reliant on local policies. (Sands_1)

Companionship was noted to have practical as well as emotional implications for women/birthing people. Some of the documents claimed that the absence of companions meant that women/birthing people required more support from maternity care professionals (e.g. RCOG/RCM_1j; NHSE_1) creating additional stress for over stretched services (RCOG/RCM_1l), and additional potential exposure to COVID infection. Some practitioner respondents also reported that they or their colleagues experienced moral distress when social

For peer review only - http://bmjopen.bmj.com/site/about/guidelines.xhtml

distancing rules prohibited physical contact with women/birthing people who were alone, or receiving bad news:

> All of a sudden it was just women on their own for us. And that was really, really stressful for the women and the staff. And a lot of a lot of my job is giving bad news. And to give that to women that are on their own with no support; you can't touch them. You can't hug them. And so that for us is really, really challenging. I think that was probably the most challenging thing. (Stakeholder 24, Sonographer)

Need for flexibility

Concerns discussed within the documents (e.g. BR_8; BR_18; RCM_4) and raised by stakeholders related to the blanket adoption of visitation rules across whole hospitals. Some commented that pregnant women/birthing people were a *'separate population with separate needs'* (Stakeholder 7, Service-user organisation), arguing that visiting rules adopted in other areas of healthcare should not apply to a perinatal population:

> So, you know, several heads of midwifery were saying to me, I want to do this, but they won't let me because they made a decision about what the visiting will look like in this hospital. And they do not see maternity as an exception. And, you know, it is an interesting reflection, isn't it, that maternity has always been a service that has seen itself as an exception to the health care service in which it sits. (Stakeholder 17, NHS improvement lead)

Responses from organisations including AIMS, Birthrights, and the RCM argued how there needed to be consideration of women's/birthing people's unique situations. Restrictions on companionship were considered to have a disproportionate impact on those who were facing disadvantages including those for whom English is a second language, those with mental health problems, cognitive impairments, refugee and asylum seekers (AIMS_8, RCM_4, BR_8, BR_18). Only one Trust included a statement on their website about offering personalised (flexible/individualised) care for all women/birthing people, that might not be in line with COVID policies. Five others said they offered this on a case-by-case basis (often expressed as 'exceptional circumstances'). One of these referred to Black, Asian, and Minority Ethnic communities along with concerns about greater COVID risks, and three referred to *'allowing'*

For peer review only - http://bmjopen.bmj.com/site/about/guidelines.xhtml

women/birthing people to bring a companion if they *'needed assistance'*. In two Trusts this was explicitly linked to those with disabilities:

> Partners and family members will not be allowed to enter the building unless you need support from a carer/relative (for example, if you have a disability and need support) (North West 2).

Birthrights stipulated how 'protected characteristics' under the Equality Act 2010 (e.g. physical disability or mental health condition) meant that maternity Trusts were obliged to make reasonable adjustments (e.g. BR_15; BR_18). NHS England emphasised the need for *'essential visitors'* (seen as different to 'normal visitors') for those with specific communication or care needs (NHSE_8). AIMS also stressed that on some occasions, on a '*case by case basis*', such as a disability, *'a second birth partner may be critical to women's mental wellbeing or other needs'* (AIMS_2).

The lack of flexibility for highly sensitive events such as experiences of trauma or loss were also highlighted by Birthrights (e.g. BR_18) and RCM (e.g. RCM_41). While some incidents of positive practice were identified, stakeholders also shared stories of those whose baby had died in utero being unable to take photographs or spend time with their deceased child:

> We had a lot of stories from parents who hadn't been allowed to take photographs, haven't had time to sit and hold their baby. And I think all of those were linked to both a lack of space, to lack of bereavement space, but also a lack of staff understanding of how to adapt bereavement care standards. We also saw in this group a lot of problems around not having the partner with them. (Stakeholder 10, National charity)

'Acceptable' time for support

Access to and timing of support from companions was an issue at key stages during the perinatal journey, and notably during antenatal ultrasound appointments and during early onset of labour. A key area of contention related to women/birthing people having to attend ultrasound scans unaccompanied: a situation described by one of the stakeholders as *'ludicrous'* (Stakeholder 7, Service-user organisation). RCOG guidance recommended that *'patients should be asked to attend alone if possible or with a maximum of one partner/visitor'* (RCOG_5), whereas a RCM document stated *'partners should attend scans unless rooms are*

For peer review only - http://bmjopen.bmj.com/site/about/guidelines.xhtml

too small to socially distance: partners may attend scans virtually' (RCM_28). However, in contrast to this permission for virtual contact, a joint statement by SoR, RCOG, RCM and the British Medical Ultrasound Society stated that devices required for remote contact by companions via video/phones are a vehicle for transmission (due to surface contamination), and that recordings would impact on scan time, sonographer concentration and potential detection of fetal abnormalities – although it was acceptable for the woman/birthing person (if in line with local policies) to "*save a short 10–30 second cine clip of the fetus at the end of selected examinations"* (SoR_11).

Trust data revealed that while most permitted companions at one or both standard ultrasound appointments (12 and 20 weeks), seven (~30%) did not. Four Trusts mentioned video or other means of 'virtual' companionship, but usually to specify that videos of scans were not permitted. Only one Trust referred to women/birthing people being able to phone a companion for support if the sonographer were to find "*important clinical information that your partner needs to be aware of*" (Greater London 2). While many stakeholders were critical of the ultrasound restrictions on the right to be accompanied, one respondent argued the need to highlight that antenatal ultrasound scanning continued, even when *'other screening programmes went into hibernation'* (Stakeholder 26, Radiographer). Some defended restrictions on women/birthing people being accompanied during scanning, noting that scans often have to take place in areas *'like a broom cupboard in a very small poorly ventilated space'* (Stakeholder 17, NHS improvement lead) coupled with the restricted time to undertake the examination and sterilising the room and equipment after each appointment. One reported that there had been a *'downgrading'* of the importance of scans as a medical examination that required focused concentration in challenging situations, during vociferous debate about companion attendance (Stakeholder 26, Radiographer). However, while sonographers may have faced increased risks due to screening large numbers of women/birthing people, the specific rationale for not allowing videos as an alternative was challenged:

> You can argue the toss as to whether some of the justifications for not allowing that were real or weren't real. You know, is there really a risk of infection if you pick up your phone? Really? Maybe some anxiety for sonographers or whoever's doing the scan. You know, you don't really want the phone on with a video while you're doing the scan because who knows, they might use it in some kind of litigation. Who knows? But whatever it was, it really didn't help. (Stakeholder 20, National charity)

For peer review only - http://bmjopen.bmj.com/site/about/guidelines.xhtml

A further area of contention concerned companionship during labour and birth. While organisations such as Birthrights argued for companionship throughout, less than half (9/25) the Trusts referred to companions of choice attending '*throughout*' or '*for the duration*'. Three Trusts referred to companionship being permissible only when the woman/birthing person was in '*established*' or '*active*' labour (with no details as to how this would be established); and 13 Trusts did not specify the relevant phase of labour. RCM guidance advised that women/birthing people would not be able to have companions present during inductions that took place in a bay or ward (RCM_27). Only six Trusts (25%) indicated that companions could be present during induction of labour, and four allowed companions, but with limitations (either restricted to daytime or if the woman/birthing person needed additional support). About half (12) provided no information, and three explicitly stated that companions were not allowed:

> If you are attending for induction of labour please attend alone, your birth partner will join you once you are transferred to the Delivery Suite. (Yorkshire and Humber 1)

Birthrights and AIMS (i.e. BR_8; AIMS_5; BR_18; BR_23) also raised concerns about cervical dilatation as the only acceptable indicator of active labour. As this meant that some women/birthing people who may not have wanted (or needed) a vaginal examination felt pressured to accept the procedure if they wanted their chosen companion to be granted access.

There were examples of innovation to try to support companionship. Some Trusts initiated or extended the provision of labour induction in community settings or in private hospital rooms (rather than multi-occupancy early labour wards) to prevent separation of women/birthing people from their companions (RCM_8; RCM_27). Some stakeholders also referred to more flexible approaches to induction such as companions being able to *'come and settle them* [women] *in'* and to use *'Facetime to be with their partner all the time'* (Stakeholder 21, Midwife - National role).

Loss of human rights for gain in infection control

There was some evidence from stakeholders that hospital decision makers in some settings believed that companionship during the maternity episode should not be prioritised over visiting rights in other areas where attendance of close family members would usually be seen as a critical human need and right: especially when someone was dying in hospital:

For peer review only - http://bmjopen.bmj.com/site/about/guidelines.xhtml

> So, it was interesting and when I would speak to the head of midwifery, sometimes it felt like they were saying, you know, well, everyone's got to make…. sacrifices. And there are people dying alone in hospital. There are people suffering terribly alone in hospital, unable to have visitors…. [while] there were women saying, you know, it's my right to have a companion it's your job to provide care for me. So, it felt at times like each group with their own concerns was unable to think about or found it difficult to take on board the concerns of the other group. (Stakeholder 17, NHS improvement lead)

The underlying principle within most of the guidance reviewed was that 'safety', was primarily conceptualised as the prevention of transmission of infection, for women/birthing people, companions, and staff. NHS England documents referred to minimising '*control risks working with your IPC* [Infection, Prevention & Control] *leads, whilst still allowing the maximum possible safe access'* (i.e. NHSE_9). The RCOG/RCM also noted the need to minimise the number of attendees, but acknowledged that one person could be there for antenatal visits should a woman/birthing person choose this:

> You will be asked to come alone to clinical appointments or keep the number of people with you to one (including midwifery visits in your home). This will include being asked not to bring your children with you to appointments. This is important to protect maternity staff, other women, and babies, and you and your family from the risk of infection. (RCOG/RCM_1g)

Birthrights was one of the key organisations to recommend that notions of safety might also include emotional and psychosocial risks of women/birthing people being unattended (e.g. BR_17, BR_16, BR_19, BR_18) - '*The damage caused by ongoing restrictions needs to be weighed up against the requirements of infection control'* (BR_19). Several of their documents (e.g. BR_18; BR_19) claimed that routinely restricting companions was a violation of women's/birthing people's (and companions) human rights. Despite this, stakeholders stated that, in practice, human rights and choices around companionship did not feature as part of the decision-making processes:

For peer review only - http://bmjopen.bmj.com/site/about/guidelines.xhtml

> And I think we've spent the last, you know, however many years banging on about the fact we want to give women choice and rights and sharing that discourse and encouraging women to become empowered. And then COVID comes along and we just say, no, no, we're not doing that (Stakeholder 17, NHS improvement lead)

The RCM (RCM_41) stated that their '*greatest concern*' was '*safety being sacrificed in favour of popularity*' which seemed to imply that companionship or visiting should not outweigh the need to prevent infection of its *'members'* and of '*women and families'*. SoR also highlighted that its guidance had *'risk assessments'* at its core (SoR_6). The guidance did not preclude *'people being accompanied'*, but that it '*must only happen if the safety of the patient and sonographer is not compromised*' (SoR_8). However, others argued that day to day decision making was based around a belief about safety that was limited: '*because it's not just about the physical self, it's about psychological self*' (Stakeholder 14, Midwife – National role):

> But I think safety generally is an interesting thing because….You know, so many different things affect safety don't they, so something like being able to have your partner with you might not be seen as a primary thing affecting safety in comparison with protecting against COVID, but actually if it impacts on someone's mental health in either the partner or the mother, that does have an effect on safety (Stakeholder 5, Maternity Voices Partnership)

DISCUSSION

In this paper we have drawn on guidance from national statutory and service-user organisations, key stakeholders, and public facing Trust-level data to consider the organisational issues associated with companionship and visiting in antenatal and intrapartum care during the COVID-19 pandemic. The terms companionship and visiting were not always clearly differentiated in data relating to the antenatal and intrapartum period, though most sources were consistent in referring to accompaniment as 'companionship' during labour and birth. The value of active companionship during labour and birth for women/birthing people is widely recognised, in terms of clinical benefits, and short and longer term psychosocial impacts[1, 19]. As evidenced within this paper, during the COVID-19 pandemic, at the policy and organisational level, assumptions and norms about companionship, accompaniment, and visiting during facility-based health care provision have faced profound challenge. Some of the key organisational challenges have concerned personnel shortage issues, infection control

For peer review only - http://bmjopen.bmj.com/site/about/guidelines.xhtml

and restricted space. Others have noted the variance in maternity organisation response during the pandemic(20]. Some variation can probably be explained by changing national knowledge about the prevalence and impacts of COVID-19, and by different levels of exposure to COVID-19 infection. However, our data suggest that this was not the case where blanket policies were applied with minimal individual flexibility, or where there was unjustified variation in visiting and companionship rules, coupled with poor and inconsistent communication. There were no clear patterns in the Trust-level data that would allow us to explain the differences we documented in responses. While population level disparities may be a contributing factor, most Trusts serve a range of sociodemographic/economic areas, and other potentially relevant information such as space constraints was not publicly available.

We found particular concern about lack of access to companionship (in the sense of informational, practical and social support and advocacy[1]) in two distinct areas. First, women/birthing people being unable to have any communication (actual or virtual) with companions at ultrasound scan; and, second, denial of intrapartum companionship until labour was 'established'. In relation to the former case, there is some evidence that, beyond the emotional and psychological benefits for the mother, when fathers and co-parents are present for antenatal ultrasound scan, there are significant effects on their identification with the fetus (as their future child) and their empathic relating with the woman/birthing person [21, 22]. This implies that being present for ultrasound scans is more than simply 'visiting'. It has important public health and relationship benefits for the woman/birthing person, their partner, and baby. In the latter case, in some Trusts, ensuring that labour had progressed sufficiently was perceived by some stakeholders to be associated with coercive and invasive practices, such as regular vaginal examinations when women/birthing people may otherwise not have needed or wanted such examinations. General uncertainty over organisational companionship permissions during labour and birth may also be reflected in anecdotal rises in women/birthing people choosing to freebirth[23, 24], and the associated RCM guidance to ensure appropriate professional responses[25]. Trust policies that restricted intrapartum companionship until labour was established (or until birth was imminent) seemed to be built on an assumption that companionship was only really needed when labour was very intense, and/or when the birth was happening, so that the companion could be 'permitted' to witness to the birth of the baby. In contrast, other Trusts seemed to recognise, at the organisational level, that active and engaged companionship throughout labour (from the early stages of spontaneous labour, or from the time of labour induction through to the birth) is a mechanism for clinical,

For peer review only - http://bmjopen.bmj.com/site/about/guidelines.xhtml

psychological and emotional safety for the woman/birthing person, partner and child, both in the short term, and, critically, in the longer term, when the threat of COVID-19 infection is long over [12, 26].

The pandemic brings into sharp focus the fundamental and underpinning ethical dilemma between social actions that ensure the greatest benefit for the population as a whole, and the individual human rights of each person within that population[27]. Resolving this potential conflict of ethical imperatives depends on an open and informed debate about rights and consequences. In terms of maternity care, this requires a sophisticated understanding of what 'companionship' (as opposed to 'visiting') means, over the whole life course, and for the woman/birthing person, baby, and family. It also requires attention to the potential moral distress of maternity care staff (and health care staff in general, including ultrasonographers). These professionals are faced with the stress of having to balance these two imperatives with real people, in intensely emotional real time, repeatedly day in and day out, and at times with insufficient PPE equipment available; at a time when they, too could be pregnant at risk of exposure to infection, or fearful of infecting others [28-31].

This is the first study to bring together national policy and organisational stakeholder views with Trust based public facing data to understand how companionship and visiting in antenatal and intrapartum care has been organised in England during COVID-19. Although we cannot be sure we captured every single relevant document produced over the period of our data collection, triangulation across data sources and across multiple researchers enabled rich insights into how and why variations occurred, and the perceived impacts. Returning quotes to stakeholders (as they requested), also provided a further level of rigour. The pragmatic restriction of the Trust level data collection to only 25 Trusts (10% of maternity care providers in the UK), and the restriction to maternity-specific documents and guidance may be a limitation. However, the organisations that were included were selected purposively to reflect a wide range of relevant characteristics. Trust-level data were collected during a discrete period (September and October 2020), aiming to capture responses to changed national guidance; this limitation means we do not address how Trusts continued to respond to the changing pandemic. Since this paper is focused on policy and organisational responses to the pandemic, the views of women/birthing people, companions and healthcare professionals at Trust level were not included. In addition, our analysis did not include findings related to postnatal care, or care in neonatal units. These areas, and the unintended (positive and negative) short- and longer-term

For peer review only - http://bmjopen.bmj.com/site/about/guidelines.xhtml

consequences of different interpretations of the value of companionship (in itself, and as opposed to being a hospital visitor), when balanced against infection control, are critical areas for examination during the on-going COVID-19 crisis. Future outputs from the ASPIRE project will address these gaps.

CONCLUSION

This paper presents insights from the ASPIRE COVID-19 UK study to understand how companionship and visiting in maternity care was operationalised at the organisational level in antenatal and intrapartum care during COVID-19. Our findings illustrate variations in policy at national and local level, coupled with poor and inconsistent communication of how the restrictions changed in some sites, and a lack of clarity in the decision-making processes. The evidence highlights a lack of flexibility in responding to women/birthing people with more complex needs, the negative and positive unintended consequences of companionship restrictions, and the challenges of conceptualising and balancing infection risk and emotional and psychological distress. However, there was evidence that creative solutions were possible, since, despite significant pressures, some Trusts appeared to continue to provide full companionship.

Overall, these concerns illustrate something much more fundamental than merely barriers to hospital 'visiting'. While the NHS England Better Births policy agenda highlights the need for safety and personalisation within maternity care, these findings suggest that, over the time period captured by this study, personalisation (and emotional and psychological safety) became sacrificed in some (but not all) situations to the overriding imperative to minimise infection spread with high emotional and psychological costs. Further research should capture the views and experiences of healthcare professionals, women/birthing people, and clinical outcome data from different settings. There is an urgent need to determine how to balance risks and benefits sensitively and flexibly and to create optimum outcomes for women/birthing people, companions (including fathers, co-parents, and others), infants, families, and staff, during the current and future crisis situations.

ACKNOWLEDGEMENTS

A funding statement: This research is funded by the Economic and Social Research Council (ESRC), as part of UK Research and Innovation's rapid response to COVID-19 [grant

For peer review only - http://bmjopen.bmj.com/site/about/guidelines.xhtml

number ES/V004581/1]. Full details of the main study are available via ResearchRegistry (researchregistry5911) and via UKRI Gateway (https://gtr.ukri.org/projects?ref=ES%2FV004581%2F1)

Author contributions: SD and AT with input from the ASPIRE-COVID19 Collaborative Group designed the study. RN, GT, SD, and NC interviewed the stakeholders; GT, MCB, GM and JC were involved in data extraction for the documentary analysis; SH, AS and NC collected and analysed Trust level data. GT, MC, and RN were involved in developing themes from the interview and documentary data, and GT synthesised the Trust level data into the data set. Final themes were agreed with all authors. All authors and the ASPIRE-COVID19 Collaborative Group contributed to writing and reviewing the manuscript.

A data sharing statement: Trust level data is included in the paper. Details of all documents analysed is provided/with all information freely available. All relevant interview data concerning companionship are available in UCLanData repository at https://doi.org/10.17030/uclan.data.00000319. [32]

Competing interest statement: None of the authors have any competing interests.

REFERENCES

1. Bohren MA, Berger BO, Munthe-Kaas H, Tunçalp Ö. Perceptions and experiences of labour companionship: a qualitative evidence synthesis. *Cochrane Database Syst Rev* 2019;3, doi: 10.1002/14651858.CD012449.pub2

2. Jordan B. Birth in four cultures: A crosscultural investigation of childbirth in Yucatan, Holland, Sweden, and the United States: Waveland Press; 1992.

3. Tani F, Castagna V. Maternal social support, quality of birth experience, and post-partum depression in primiparous women. *J Matern Fetal Neonatal Med* 2017;30(6):689-92.

4. Withers M, Kharazmi N, Lim E. Traditional beliefs and practices in pregnancy, childbirth and postpartum: A review of the evidence from Asian countries. Midwifery. 2018;56:158-70.

5. Draper J. Whose welfare in the labour room? A discussion of the increasing trend of fathers' birth attendance. Midwifery. 1997;13(3):132-8.

For peer review only - http://bmjopen.bmj.com/site/about/guidelines.xhtml

6. Jackson SH, Schlichting FR, Hulme RL. Effect of fathers at cesarean birth on postpartum infection rates. AORN 1982;36(6):973-7.

7. Stephan G, Silbermann B. Father in delivery room wins decision that could land you in court. Legal aspects of medical practice. 1979;7(7):49-50.

8. Kabakian-Khasholian T, Portela A. Companion of choice at birth: factors affecting implementation. BMC Preg Childbirth. 2017;17(1):1-13.

9. World Health Organization. WHO recommendations: intrapartum care for a positive childbirth experience: transforming care of women and babies for improved health and well-being. World Health Organization; 2018.

10. Redshaw M, Henderson J. Fathers' engagement in pregnancy and childbirth: evidence from a national survey. BMC Preg childbirth. 2013;13(1):1-15.

11. Care Quality Commission. 2019 survey of women's experiences of maternity care https://www.cqc.org.uk/sites/default/files/20200128_mat19_statisticalrelease.pdf (Accessed 20 August 2021)

12. Coulter A, Richards T. Care during covid-19 must be humane and person centred.*BMJ* 2020;370:m348

13. Ferber A. How Covid-19 led to a global crisis in maternity care. New Statesman. 17 December 2020 How Covid-19 led to a global crisis in maternity care (newstatesman.com)

14. Black BL, J; McKay, G. Birth partners are not a luxury BMJ 24 September 2020 https://blogs.bmj.com/bmj/2020/09/24/birth-partners-are-not-a-luxury/

15. National Statistics. English indices of deprivation 2019. English indices of deprivation 2019 - GOV.UK (www.gov.uk) (Accessed 15 August 2021)

For peer review only - http://bmjopen.bmj.com/site/about/guidelines.xhtml

16. Vaismoradi M, Turunen H, Bondas T. Content analysis and thematic analysis: Implications for conducting a qualitative descriptive study. *Nurs Health Sci* 2013;15(3):398-405.

17. Graneheim UH, Lundman B. Qualitative content analysis in nursing research: concepts, procedures and measures to achieve trustworthiness. *Nurse Educ* 2004;24(2):105-12.

18. Iacobucci G. Partners' access to scans and birth is a postcode lottery, data show. *BMJ* 2020;371:m3876

19. WOrld Health Organization. Companion of choice during labour and childbirth for improved quality of care: evidence-to-action brief. World Health Organization; 2016.

20. Jardine J, Relph S, Magee LA, von Dadelszen P, Morris E, Ross-Davie M, et al. Maternity services in the UK during the coronavirus disease 2019 pandemic: a national survey of modifications to standard care. BJOG. 2021;28(5):880-889.

doi: 10.1111/1471-0528.16547.

21. Walsh TB, Davis RN, Palladino CL, Romero VC, Vijay Singh M. Moving up the" magic moment": Fathers' experience of prenatal ultrasound. *Fathering* 2014;12(1):18.

22. Draper J. 'It's the first scientific evidence': men's experience of pregnancy confirmation. *J Adv Nurs* 2002;39(6):563-70.

23. Dahlen H. During COVID-19, women are opting for 'freebirthing' if homebirths aren't available. And that's a worry: The Conversation 2020 9 July 2020 https://theconversation.com/during-covid-19-women-are-opting-for-freebirthing-if-homebirths-arent-available-and-thats-a-worry-142261.

24. Greenfield M, Payne-Gifford S, McKenzie G. Between a rock and a hard place: Considering "freebirth" during Covid-19. *Frontiers in Global Women's Health*. 2021;2:5 https://doi.org/10.3389/fgwh.2021.603744.

For peer review only - http://bmjopen.bmj.com/site/about/guidelines.xhtml

25. Royal College of Midwives. Clinical Briefing Sheet: 'freebirth' or 'unassisted childbirth' 30 April 2020 https://www.rcm.org.uk/media/3904/freebirth_draft_23-april-v5-002-mrd-1.pdf.

26. Lavender T, Downe S, Renfrew M, et al. Rapid analytic review: Labour and birth companionship in a pandemic: Companionship of choice for asymptomatic childbearing women in hospital throughout labour and birth. *Royal College of Midwives* https://www.rcm.org.uk/media/3951/birth-companionship-in-a-pandemic-master-27-04-2020-002.pdf (Accessed 23 February 2021)

27. Xafis V, Schaefer GO, Labude MK, Zhu Y, Hsu LY. The perfect moral storm: Diverse ethical considerations in the COVID-19 pandemic. *Asian Bioeth Rev* 2020;12:65-83.

28. Hunter B, Renfrew MJ, Downe S, et al. Supporting the emotional wellbeing of midwives in a pandemic: Guidance for RCM. 2020. Royal College of Midwives. https://www.rcm.org.uk/media/4095/rcm-supporting-the-emotional-wellbeing-of-midwives-during-a-pandemic-v1-submitted-to-rcm_mrd.pdfm (Accessed 21 February 2021)

29. Jeffrey DI. Relational ethical approaches to the COVID-19 pandemic. *J Medical Ethics* 2020;46(8):495-8.

30. Sheather J, Fidler H. Covid-19 has amplified moral distress in medicine. *BMJ* 2021;372:n28

31. Chew C, Ko D. Medical ethics in the era of COVID-19: Now and the future. *Respirology* https://doi.org/10.1111/resp.13927 (Accessed 5 January 2021)

32. [dataset] Thomson G, Balaam MC, Nowland R, Crossland N, Moncrieff G, Heys S, Sarian A, Cull J, Topalidou A, Downe S. ASPIRE COVID-19 WP2 National Stakeholders Interviews UK. December 2021. https://doi.org/10.17030/uclan.data.00000319

For peer review only - http://bmjopen.bmj.com/site/about/guidelines.xhtml

Supplementary File 1

Interview schedule

Opening question

In your view, what are the most important issues for maternity care provision that have come out of COVID-19?
Probe: *Are these views based on any particularly memorable experiences?*

Changes/adaptions to service delivery

How have [i.e. maternity, midwifery, obstetric, neonatal, ambulance delivery, staffing, any other?] services been adapted during COVID-19 pandemic?
Prompts: *How significant or minor have these changes been?*
Can you describe some of the primary Covid-19 interventions?
Have these adaptions varied by place and/or time? If so, what do you think explains this variation?

Decision making processes

From your perspective, who makes decisions about the changes?

Prompts: *How are these decisions made?*
Were decisions made at a multidisciplinary level or separately?
How did the different agencies – work together - who had overall responsibility?
Who has been consulted about the changes – are all those impacted consulted (e.g. at UK level were MVPs consulted and/or for neonatal were the local PAG group consulted?)
If there is resistance, how is that dealt with?

How have safety principles (for service-users and staff) informed decision-making/practice changes?

How have personalised care principles informed decision-making/practice changes?
Prompts: *What personalised care aspects have been considered?*

To what extent has the needs and care of BAME/vulnerable populations been considered in the decision-making processes?

What other factors have influenced decision-making/changes in care provision?

Communication and implementation

How have the recommended practices and guidance been developed?
Prompts: *How effective have these methods been?*
How was conflict in what to include resolved?

How have they been communicated to practitioners/front line staff?
Prompts: *How effective have these methods been?*
What more is needed?

How is service delivery being monitored over this period?
Prompts: *What feedback loops are in place*?
How is this information used?

Impact

What has been the (positive and negative) impact of the changes/adaptions on:
- Staff
- Parents and families (siblings/grandparents)
- Service delivery
- Data collection and sharing (i.e. Maternity Services Dashboard and Better Births Minimum Data set)

In which data do you think the effects of the pandemic and the adaptations made to service provision can be seen? Can we have access to the data?

Barriers and facilitators

What has worked well in providing safe and personalised maternity care during the pandemic?
Prompts: *Do any particular Trusts/services/lobby groups and etc stand out for you?*
Why is this?

What have been the challenges or barriers in providing safe maternity care?

What have been the challenges or barriers in providing personalised maternity care?

Recommendations and Sustainability

What else is needed to ensure safe and personalised maternity standards and principles are upheld in:
- future routine maternity care
- a future crisis/pandemic

If there was one thing that you would change about how maternity care was managed what that would be?

What innovations been implemented that could be opportunities for how maternity care could be organized in the future?
Prompts: *Can you identify where these innovations and opportunities are happening at the moment?*

How do you think services should prepare for future occurrences/pandemics?

Is there a specific Trust/Health Board you think we should include in our case studies?

For peer review only

Prompts: *If so, can you tell us why you think this is a particularly relevant site?*

Are there other things, that have not been mentioned that you would like to share?

Is there anyone else that you think we should talk to?

For peer review only

Organisation/Code	Name of Document/Link	Date of documen
**AIMS**		
AIMS_1	Coronavirus and the Maternity Services on AIMS website https://www.aims.org.uk/campaigning/item/coronaviru	25/03/2020
AIMS _2	AIMS Response to the Clinical guide for the temporary reorganisation of intrapartum maternity care during the coronavirus pandemic, version 1, published 9/4/20 https://www.aims.org.uk/assets/media/444/aimscomments2020-04-11.pdf	09/04/2020
AIMS_5	Submission from AIMS to the Health and Social Care Committee Inquiry: Delivering Core NHS and Care Services during the Pandemic and Beyond<br>https://www.aims.org.uk/assets/media/447/aimssubmissionhealthandsocialcarecommittee2020-05.pdf	07/05/2020
AIMS_7	AIMS comments on latest guidance on birth partner restrictions https://www.aims.org.uk/campaigning/item/latest-guidance-birth-partner	25/09/2020
AIMS_6	Press Release: Almost Half of NHS Trusts Force Pregnant Women to Attend Vital Maternity Services Alone https://www.aims.org.uk/campaigning/item/trusts-force-pregnant-women-to-attend-alone	12/10/2020
AIMS_9	AIMS calls for clearer guidance from NHS England on lifting partner restrictions in maternity<br>https://www.aims.org.uk/campaigning/item/nhse-letter	21/11/2020
AIMS_8	AIMS asks Society and College of Radiographers to review guidance on partners and supporters at scans https://www.aims.org.uk/campaigning/item/scor-letter	12/11/2020
**Birthrights**		
BR_1	Human rights charity calls for protection of UK women in childbirth during national emergency https://www.birthrights.org.uk/wp-content/uploads/2020/03/Final-Covid-19-Birthrights-31.3.20.pdf	31/03/2020
BR_8	Coronavirus and the impact on people with protected characteristics https://www.birthrights.org.uk/wp-content/uploads/2020/05/Birthrights-Covid-19-Maternity-Briefing-women-and-equalities-committee-final.pdf	01/04/2020
BR_6	Final letter SOR-BMUS 22nd April Birthrights joint letter with National Maternity Voices, Sands, the Twins Trust and ARC https://www.birthrights.org.uk/wp-content/uploads/2020/04/Final-letter-SoR-BMUS-22-April.pdf	22/04/2020
BR_12	Letter to NHSE regarding ongoing visiting restrictions https://www.birthrights.org.uk/wp-content/uploads/2020/08/Letter-to-NHSE-re-ongoing-visiting-restrictions.pdf	20/08/2020

For peer review only

BR_14	Ongoing visitor restrictions in maternity services https://www.birthrights.org.uk/2020/09/14/ongoing-visitor-restrictions-in-maternity-services/	14/09/2020
BR_15	Template letter for ongoing visiting restrictions in maternity services https://www.birthrights.org.uk/template-letters/	14/09/2020
BR_16	Letter on restricting visitors – Sheffield teaching hospitals https://www.birthrights.org.uk/wp-content/uploads/2020/10/Letter-to-Sheffield-15th-October.pdf	15/10/2020
BR_17	"Partners are not visitors to maternity services" says Birthrights and partner organisations [joint letter on visiting restrictions to NHSE&I] https://www.birthrights.org.uk/wp-content/uploads/2020/10/Final-letter-to-NHSE-from-coalition-re-visiting-restrictions.pdf	20/10/2020
BR_19	Human rights charity calls for easing of visiting restrictions in maternity services press release https://www.birthrights.org.uk/wp-content/uploads/2020/08/Easing-of-visitor-restrictions-press-release-20-August-2020.pdf	20/08/202
BR_18	Human rights implications of changes to maternity services during the Covid-19 pandemic Birthrights briefing, July 2020 https://www.birthrights.org.uk/wp-content/uploads/2020/07/Birthrights-Covid-19-Human-Rights-Committee-Briefing-July-2020.pdf	Jul-20
BR_23	Long term impact of visiting restrictions "could be catastrophic" MPs, academics and campaigners warn NHS England CEO... https://www.birthrights.org.uk/2020/11/15/long-term-impact-of-visiting-restrictions-could-be-catastrophic-mps-academics-and-campaigners-warn-nhs-england-ceo/	15/11/2020
**ISUOG**		
ISUOG_6	ISUOG Safety Committee Position Statement: safe performance of obstetric and gynaecological scans and equipment cleaning in the context of COVID-19 https://www.isuog.org/resource/isuog-safety-committee-position-statement-safe-performance-of-obstetric-and-gynecological-scans-and-equipment-cleaning-in-the-context-of-covid-19.html	23/03/2020
ISUOG_5	ISUOG Consensus Statement on organization of routine and specialist obstetric ultrasound services in the context of COVID-19 https://www.isuog.org/resource/wiley-isuog-consensus-statement-on-organization-of-routine-and-specialist-obstetric-ultrasound-services-in-the-context-of-covid-19.html	31/03/2020

For peer review only

ISUOG_3	ISUOG Interim Guidance (COVID-19) during pregnancy & puerperium: Information for healthcare professionals – an update https://obgyn.onlinelibrary.wiley.com/doi/full/10.1002/uog.22061	01/05/2020
**NHS England**		
NHSE_1	Clinical guide for the temporary reorganisation of intrapartum maternity care during the coronavirus pandemic<br>https://madeinheene.hee.nhs.uk/Portals/0/Clinical%20guide%20for%20the%20temporary%20reorganisation<br>%20of%20intrapartum%20maternity%20care.pdf	09/04/2020
NHSE_2	Delivering midwifery intrapartum care where local COVID-19 escalation protocols are required to be enacted<br>https://www.nice.org.uk/media/default/about/covid-19/specialty-guides/delivering-midwifery-intrapartum-care-where-local-covid-19-escalation-protocols-are-required-to-be-enact.pdf	20/06/2020
NHSE_8	Framework to assist NHS trusts to reintroduce access for partners, visitors and other supporters of pregnant women in English maternity services http://allcatsrgrey.org.uk/wp/download/midwifery/par001599-framework-for-the-reintroduction-of-visitors-throughout-maternity-services-sep-2020.pdf	08/09/2020
NHSE_9	Letter to directors of nursing and heads of midwifery<br>(No longer available on website, available on request)	19/09/2020
NHSE_10	Lateral flow antigen test for pregnant women and support people: Frequently asked questions<br>https://www.england.nhs.uk/coronavirus/wp-content/uploads/sites/52/2020/12/C0972-lateral-flow-antigen-tests-for-maternity-faqs-december-2020.pdf	21/12/2020
NHSE_11	Supporting pregnant women using maternity services during the coronavirus pandemic: Actions for NHS providers Version 1, 14 December 2020<br>https://www.england.nhs.uk/coronavirus/wp-content/uploads/sites/52/2020/12/C0961-Supporting-pregnant-women-using-maternity-services-during-the-coronavirus-pandemic-actions-for-NHS-provi.pdf?fbclid=IwAR0w9hbJSbH1gYYPVhGtE8zRAvwd8PidPkPsEaDnhxzlSumFl7t6byshst8	14/12/2020
**RCOG**		
RCOG/RCM_1c	Coronavirus (COVID-19) Infection in Pregnancy Information for healthcare professionals Version 3<br>https://www.rcm.org.uk/media/3799/coronavirus-covid-19-infection-in-pregnancy-v3-20-03-18.pdf	18/03/2020
RCOG/RCM_1d	Coronavirus (COVID-19) Infection in Pregnancy Information for healthcare professionals Version 4<br>https://www.rcm.org.uk/media/3800/2020-03-21-covid19-pregnancy-guidance.pdf	21/03/2020

RCOG/RCM_1e	Coronavirus (COVID-19) Infection in Pregnancy Information for healthcare professionals Version 5<br>https://www.rcm.org.uk/media/3824/2020-03-28-covid19-pregnancy-guidance.pdf	28-03-202
RCOG/RCM_1f	Coronavirus (COVID-19) Infection in Pregnancy Information for healthcare professionals Version 6<br>http://www.e-lactancia.org/media/papers/2020-04-03-coronavirus-covid-19-infection-in-pregnancy.pdf	03/04/2020
RCOG/RCM_1g	Coronavirus (COVID-19) Infection in Pregnancy Information for healthcare professionals Version 7<br>https://www.sbmfc.org.br/wp-content/uploads/2020/04/2020-04-09-coronavirus-covid-19-infection-in-pregnancy.pdf	09/04/2020
RCOG_9	Guidance for rationalising early pregnancy services in the evolving coronavirus (COVID-19) pandemic<br>https://www.rcog.org.uk/globalassets/documents/guidelines/2020-05-15-guidance-for-rationalising-early-pregnancy-services-in-the-evolving-coronavirus-covid-19-pandemic.pdf	15/05/2020
RCOG_11	Framework for staffing of obstetrics and gynaecology units during the COVID-19 pandemic<br>https://www.rcog.org.uk/globalassets/documents/guidelines/2020-05-22-framework-for-staffing-of-obstetrics-and-gynaecology-units-during-the-covid-19-pandemic.pdf	22/05/2020
RCOG_6	Principles for the testing and triage of women seeking maternity care in hospital settings, during the COVID-19 pandemic<br>https://www.rcog.org.uk/globalassets/documents/guidelines/2020-05-29-principles-for-the-testing-and-triage-of-women-seeking-maternity-care-in-hospital-settings-during-the-covid-19-pandemic.pdf	29/05/2020
RCOG/RCM_1j	Coronavirus (COVID-19) Infection in Pregnancy Information for healthcare professionals Version 10 https://www.rcm.org.uk/media/4113/2020-06-04-coronavirus-covid-19-infection-in-pregnancy.pdf	04/06/2020
RCOG_7b	Guidance for antenatal and postnatal services in the evolving coronavirus (COVID-19) pandemic<br>https://www.rcog.org.uk/globalassets/documents/guidelines/2020-07-10-guidance-for-antenatal-and-postnatal.pdf	10/07/2020
RCOG_14	Restoration and Recovery: Priorities for Obstetrics and Gynaecology Version 2.1<br>https://www.rcog.org.uk/globalassets/documents/guidelines/2020-06-26-restoration-and-recovery---priorities-for-obstetrics-and-gynaecology.pdf	26/06/2020
RCOG/RCM_1k	Coronavirus (COVID-19) infection in pregnancy Information for healthcare professionals Version 11 https://www.rcog.org.uk/globalassets/documents/guidelines/2020-07-24-coronavirus-covid-19-infection-in-pregnancy.pdf	24/07/2020

RCOG_2	Guidance for provision of midwife-led settings and home birth in the evolving coronavirus (COVID-19) pandemic Version 1.3<br>https://www.rcog.org.uk/globalassets/documents/guidelines/2020-07-10-guidance-for-provision-of-midwife-led.pdf	10/07/2020
RCOG_4	Guidance for fetal medicine units (FMUs) in the evolving coronavirus (COVID-19) pandemic<br>https://www.rcog.org.uk/globalassets/documents/guidelines/2020-11-23-guidance-for-fetal-medicine-units.pdf	10/07/2020
RCOG_5	Guidance for antenatal screening and ultrasound in pregnancy in the evolving coronavirus (COVID-19) pandemic<br>https://www.rcog.org.uk/globalassets/documents/guidelines/2020-07-10-guidance-for-antenatal-screening.pdf	10/07/2020
RCOG_15	Joint RCOG & RCM Statement - Planning for Winter 2020/21: reducing the impact of COVID-19 on maternity services in the UK<br>https://www.rcog.org.uk/globalassets/documents/guidelines/2020-10-08-rcog_rcm_winter_secondwave_statement.pdf	08/10/2020
RCOG_7	Guidance for antenatal and postnatal services in the evolving coronavirus (COVID-19) pandemic<br>Version 3<br>https://www.rcog.org.uk/globalassets/documents/guidelines/2020-10-21-guidance-for-antenatal-and-postnatal-services-in-the-evolving-coronavirus-covid-19-pandemic-v3.pdf	21/10/2020
RCOG/RCM_1	Coronavirus (COVID-19) infection in pregnancy Information for healthcare professionals Version 12 https://www.rcm.org.uk/media/4383/2020-10-14-coronavirus-covid-19-infection-in-pregnancy-v12.pdf	14/10/2020
RCOG_17	RCOG responds to revised NHS coronavirus guidelines<br>https://www.rcog.org.uk/en/news/pregnant-women-allowed-partner-support-at-all-times-in-updated-nhs-guidelines/	16/12/2020
**RCM**		
RCM_8	Induction of labour in a pandemic: A rapid analytic scoping review<br>https://www.rcm.org.uk/media/3924/professional-clinical-briefing-no-7-intrapartum-care-with-symptomsmrd010520.pdf	04/04/2020
RCM_2	Optimising maternity services and maternal and newborn outcomes in a pandemic. A rapid analytic scoping review<br>https://www.rcm.org.uk/media/3869/rapid-review-optimising-maternity-services-for-rcm-v4-8-april.pdf	08/04/2020

RCM_15	Guidance for midwives, student midwives and maternity support workers providing community-based care during the Covid-19 pandemic https://www.rcm.org.uk/media/3900/home-visit-guidance-for-midwives.pdf	09/04/2020
RCM_11	Rapid Analytic Review: Labour and Birth Companionship in a pandemic https://www.rcm.org.uk/media/3951/birth-companionship-in-a-pandemic-master-27-04-2020-002.pdf	27/04/2020
RCM_12	RCM Clinical Briefing Sheet: 'freebirth' or 'unassisted childbirth' during the COVID-19 pandemic https://www.rcm.org.uk/media/3923/freebirth_draft_30-april-v2.pdf	30/04/2020
RCM_16	Optimising mother-baby contact and infant feeding in a pandemic. Rapid analytic review https://www.rcm.org.uk/media/4096/optimising-infant-feeding-and-contact-rapid-review-19th-may-2020-submitted.pdf	19/05/2020
RCM_9	Face-coverings and care in labour for all women https://www.rcm.org.uk/media/4151/clinical-briefing-face-mask.pdf	01/07/2020
RCM_5	Antenatal Care for women without suspected or confirmed COVID-19 and living in a symptom free household https://www.rcm.org.uk/media/4180/clinical-guidance-briefing-one-antenatal-care-for-women-without-symptomsfinalv4-1.pdf	17/07/2020
RCM_4	RCM Briefing on Re-introduction of visitors to Maternity Units across the UK during the COVID-19 pandemic https://www.rcm.org.uk/media/4161/rcm-briefing-on-reintroduction-of-visitors-to-maternity-units-in-the-covid-pandemic-003.pdf	15/07/2020
RCM_3	Virtual consultations https://www.rcm.org.uk/media/4192/virtual-consultations-v20-24-july-2020-review-24-august-2020-1.pdf	24/07/2020
RCM_7	Guidance for intrapartum care for women with COVID-19 https://www.rcm.org.uk/media/4193/intrapartum-care_with-covid-19_20200724-v9-1.pdf	24/07/2020
RCM_10	RCM Clinical Briefing Sheet - Waterbirth during the COVID-19 Pandemic https://www.rcm.org.uk/media/4188/waterbirth-during-covid-19-july-20.pdf	19/07/2020
RCM_31	RCM & RCOG urge NHS England to publish guidance on partners during pregnancy https://www.rcm.org.uk/media-releases/2020/september/rcm-and-rcog-urge-nhs-england-to-publish-guidance-on-partners-during-pregnancy	08/09/2020

For peer review only

RCM_27	Birth partners https://www.rcm.org.uk/media/4339/birth-partners-v2-220920.pdf	20/09/2020
RCM_30	Leading Royal Colleges urge the NHS to learn lessons and avoid redeploying maternity staff ahead of second wave https://www.rcog.org.uk/en/news/leading-royal-colleges-urge-the-nhs-to-learn-lessons-and-avoid-redeploying-maternity-staff-ahead-of-second-wave/	30/09/2020
RCM_28	RCM letter to The Times responding to Times column on COVID rules and pregnancy https://www.rcm.org.uk/media-releases/2020/september/rcm-letter-to-the-times-responding-to-times-column-on-covid-rules-and-pregnancy/	02/10/2020
RCM_38	Partners and visitors<br>https://www.rcm.org.uk/partners-and-visitors/	Oct-20
RCM_39	Reduce infection risks to keep pregnant women and their babies safe, says RCM https://www.rcm.org.uk/media-releases/2020/october/reduce-infection-risks-to-keep-	02/11/2020
RCM_41	Midwives call for common sense on maternity visiting guidance<br>https://www.rcm.org.uk/media-releases/2020/december/midwives-call-for-common-sense-on-maternity-visiting-guidance/	15/12/2020
**Sands**		
Sands_1	Sands feedback: experiences of pregnant women, bereaved parents and families during the COVID-19 pandemic. No longer on website, available on request	19-05-2020 to<br>27/05/2020
Sands_1	Written evidence from SANDS to parliamentary committee Written evidence submitted by SANDS (DEL0092) https://committees.parliament.uk/writtenevidence/4073/html/	May-20
Sands_4	New NHS guidance on partners of pregnant women in England attending scans https://www.sands.org.uk/about-sands/media-centre/news/2020/09/new-nhs-guidance-partners-pregnant-women-england-attending	09/09/2020
**SoR**		
SoR_11	Obstetric ultrasound examinations during the COVID-19 pandemic.<br>Recording of NHS examinations and the use of private ultrasound clinics: A joint statement from the Society and College of Radiographers (SCoR), the Royal College of Midwives (RCM), the Royal College of Obstetricians and Gynaecologists (RCOG) and the British Medical Ultrasound Society (BMUS). https://archive.sor.org/sites/default/files/document-versions/obstetric_ultrasound_examinations_during_the_covid-19_pandemic_v2_0.pdf	24/04/2020

For peer review only

SoR_9	Ultrasound: Frequently asked questions (FAQs) version 1.7 previously on website – superseded by later versions (SoR & BMUS)	27/04/2020
SoR_10	Ultrasound: Frequently asked questions (FAQs) version 1.8 previously on website – superseded by later versions (SoR & BMUS)	21/05/2020
SoR_12	Obstetric ultrasound examinations during the COVID-19 pandemic.<br>Recording of NHS examinations and the use of private ultrasound clinics: A joint statement from the Society and College of Radiographers (SCoR), the Royal College of Midwives (RCM), the Royal College of Obstetricians and Gynaecologists (RCOG) and the British Medical Ultrasound Society (BMUS). https://archive.sor.org/sites/default/files/document-versions/obstetric_ultrasound_examinations_during_the_covid-19_pandemic_v2_0.pdf	27/05/2020
SoR_6	SoR demands NHS Trusts defend sonographers from populist attacks<br>https://www.sor.org/news/sor-demands-nhs-trusts-defend-sonographers-populist-attacks	04/11/2020
SoR_4	Ultrasound: Frequently asked questions (FAQs) version 1.10 previously on website – superseded by later versions (SoR & BMUS)	04/11/2020
SoR_7	Letter from SoR Resisting populist pressure to minimise the risks to ultrasound staff & patients.<br>Document supplied by SoR	06/11/2020
SoR_8	Webpage: New national guidance on partners attending antenatal scans<br>https://www.sor.org/news/obstetrics-and-gynaecology/new-national-guidance-on-partners-attending-antena	16/12/2020

For peer review only

t

For peer review only

Supplementary File 3: Documentary and interview data sources for the six key themes

Themes	***Documents***	***Stakeholders***
Postcode lottery of care	BR_8, BR_12, BR_17, BR_19, BR_18; RCOG/RCM_1e, RCOG_9, RCOG_15, RCM/RCOG_1l, RCOG_15, RCOG_17; Sands_1; RCM_5, RCM_7, RCM_31, RCM_38, RCM_39, RCM_41; NHSE_8, NHSE_9, NHSE_11; AIMS_2,AIMS_7, AIMS_8; SoR_2, SoR_4, SoR_5, SoR_6, SoR_7, SoR_8, SoR_9, SoR_10, SoR_11, SoR_12	7, 12, 13, 15, 17, 18, 20
Confusion and stress around 'rules'	BR_8, BR_12, BR_14, BR_16, BR_19; RCM_31; AIMS_7	12, 15, 19 20
Unintended Consequences	BR_1, BR_8, BR_12, BR_16, BR_17, BR_23; RCOG/RCM_1e, RCOG/RCM_1j , RCOG/RCM_1k, RCOG/RCM_1l; Sands_1; RCM_2, RCM_7, RCM_8, RCM_11, RCM_16 ; NHSE_1, NHSE_2, , NHSE_11; AIMS_8;	2, 4, 5, 8, 12, 13, 15, 17, 18, 19, 20
Need for flexibility	BR_8, BR_6, BR_15, BR_18; Sands_1; RCM_4, RCM_41; NHSE_8; AIMS_2, AIMS_8; SoR_12	3, 7, 10, 13, 17, 21
Acceptable time for support	BR_1, BR_6, BR_8, BR_16, BR_17, BR_18, BR_23; RCOG_7; Sands_1; RCM_3, RCM_8, RCM_11, RCM_27, RM_28; AIMS_2, AIMS _5, AIMS_8; SoR_2, SoR_4, SoR_5, SoR_9, SoR_10, SoR_11, SoR_12	5, 7, 13, 15, 16, 17, 18, 19, 20, 21, 26
Loss of human rights for gain in infection control	BR_16, BR_17, BR_19, BR_23, BR_8, BR_18; RCOG/RCM_1g , RCOG/RCM_1c, RCOG/RCM_1e, RCOG/RCM_1f , RCOG_9, RCOG_6; RCM_11, RCM_30, RCM_28,RCM_41, RCM_39, RCM-38, RCM_11; NHSE_9, NHSE_10; SoR_2, SoR_6, SoR_5, SoR_8, SoR_7, SoR_4, SoR_9, SoR_10, SoR_11, SoR_12; ISUOG 5, ISUOG_6	3, 5, 11, 14, 17

For peer review only

COREQ (COnsolidated criteria for REporting Qualitative research) Checklist

A checklist of items that should be included in reports of qualitative research. You must report the page number in your manuscript where you consider each of the items listed in this checklist. If you have not included this information, either revise your manuscript accordingly before submitting or note N/A.

Topic	Item No.	Guide Questions/Description	Reported on Page No.
**Domain 1: Research team and reflexivity**			
Personal characteristics			
Interviewer/facilitator	1	Which author/s conducted the interview or focus group?	7
Credentials	2	What were the researcher's credentials? E.g. PhD, MD	Title
Occupation	3	What was their occupation at the time of the study?	Title/7
Gender	4	Was the researcher male or female?	7
Experience and training	5	What experience or training did the researcher have?	Title/7
Relationship with participants			
Relationship established	6	Was a relationship established prior to study commencement?	7
Participant knowledge of the interviewer	7	What did the participants know about the researcher? e.g. personal goals, reasons for doing the research	7
Interviewer characteristics	8	What characteristics were reported about the inter viewer/facilitator? e.g. Bias, assumptions, reasons and interests in the research topic	7
**Domain 2: Study design**			
Theoretical framework			
Methodological orientation and Theory	9	What methodological orientation was stated to underpin the study? e.g. grounded theory, discourse analysis, ethnography, phenomenology, content analysis	5
Participant selection			
Sampling	10	How were participants selected? e.g. purposive, convenience, consecutive, snowball	6
Method of approach	11	How were participants approached? e.g. face-to-face, telephone, mail, email	6
Sample size	12	How many participants were in the study?	7
Non-participation	13	How many people refused to participate or dropped out? Reasons?	7
Setting			
Setting of data collection	14	Where was the data collected? e.g. home, clinic, workplace	6
Presence of non-participants	15	Was anyone else present besides the participants and researchers?	N/A
Description of sample	16	What are the important characteristics of the sample? e.g. demographic data, date	7
Data collection			
Interview guide	17	Were questions, prompts, guides provided by the authors? Was it pilot tested?	6/Supp File
Repeat interviews	18	Were repeat inter views carried out? If yes, how many?	N/A
Audio/visual recording	19	Did the research use audio or visual recording to collect the data?	6
Field notes	20	Were field notes made during and/or after the inter view or focus group?	N/A
Duration	21	What was the duration of the inter views or focus group?	6
Data saturation	22	Was data saturation discussed?	6
Transcripts returned	23	Were transcripts returned to participants for comment and/or	6

For peer review only

Topic	Item No.	Guide Questions/Description	Reported on Page No.
		correction?	
**Domain 3: analysis and findings**			
Data analysis			
Number of data coders	24	How many data coders coded the data?	6
Description of the coding tree	25	Did authors provide a description of the coding tree?	11
Derivation of themes	26	Were themes identified in advance or derived from the data?	6
Software	27	What software, if applicable, was used to manage the data?	Excel/6
Participant checking	28	Did participants provide feedback on the findings?	6
Reporting			
Quotations presented	29	Were participant quotations presented to illustrate the themes/findings? Was each quotation identified? e.g. participant number	12-20
Data and findings consistent	30	Was there consistency between the data presented and the findings?	12-20
Clarity of major themes	31	Were major themes clearly presented in the findings?	12-20
Clarity of minor themes	32	Is there a description of diverse cases or discussion of minor themes?	All data includ

Developed from: Tong A, Sainsbury P, Craig J. Consolidated criteria for reporting qualitative research (COREQ): a 32-item checklist for interviews and focus groups. *International Journal for Quality in Health Care*. 2007. Volume 19, Number 6: pp. 349 – 357

Once you have completed this checklist, please save a copy and upload it as part of your submission. DO NOT include this checklist as part of the main manuscript document. It must be uploaded as a separate file.

For peer review only
